# Molybdenum doping enhances backbonding at neighboring iron centers in iron-sulfur clusters

**DOI:** 10.1016/j.chempr.2026.103134

**Published:** 2026-07-30

**Authors:** Alexandra C. Brown, Samantha N. MacMillan, Alex McSkimming, Denis Leshchev, Eli Stavitski, Kyle M. Lancaster, Daniel L.M. Suess

**Affiliations:** 1 Department of Chemistry, Massachusetts Institute of Technology, Cambridge, MA 02139, USA; 2 Department of Chemistry and Chemical Biology, Baker Laboratory, Cornell University, Ithaca, NY 14853, USA; 3 National Synchrotron Light Source II, Brookhaven National Laboratory, Upton, NY 11973, USA; 4 Department of Chemistry, University of California, Berkeley, Berkeley, CA, USA; 5 Present address: Department of Chemistry, Tulane University, New Orleans, LA, USA; 6 Lead contact

## Abstract

Nitrogenases employ Fe-S co-factors to reduce the weakly π-acidic ligand N_2_ to NH_3_. The Mo nitrogenase is more efficient than the V and Fe-only nitrogenases, but how the Mo ion enhances N_2_ reduction is not fully understood. In this study, we undertake a comparative structural and spectroscopic analysis of synthetic [MoFe_3_S_4_] and [Fe_4_S_4_] clusters that ultimately reveals how the presence of Mo impacts the ability of Fe-S clusters to interact with π-acids. Specifically, we find that compared with the Fe centers in the [Fe_4_S_4_] clusters, those in the [MoFe_3_S_4_] clusters are more adept at backbonding to their weakly π-acidic *N*-heterocyclic carbene ligands. Such differences can be attributed to enhanced metal-sulfide mixing and Mo–Fe delocalization in the [MoFe_3_S_4_] clusters, whereby the diffuse Mo 4d orbitals more efficiently transfer electron density from the sulfides to the Fe ions and, ultimately, to Fe-bound π-acids.

## THE BIGGER PICTURE

The three nitrogenase isozymes—the molybdenum, vanadium, and iron-only nitrogenases—are the only biological catalysts capable of binding and activating dinitrogen. Collectively, they annually produce hundreds of millions of tons of bioavailable ammonia. All three are structurally similar and use bespoke iron-sulfur clusters to carry out the most chemically challenging step: binding and transferring electron density to dinitrogen. Interestingly, the molybdenum nitrogenase is the most efficient. However, dinitrogen is not thought to bind at molybdenum, and this observation therefore raises the question of how a “spectator” molybdenum ion could have such a profound effect on the efficiency of dinitrogen reduction at iron. To investigate this question, we systematically characterized two sets of synthetic iron-sulfur clusters that model the nitrogenase catalytic co-factors: one that contains only iron ([Fe_4_S_4_]) and one in which an iron site is swapped for a molybdenum ion ([MoFe_3_S_4_]). In both series, some or all of the iron centers are bound by *N*-heterocyclic carbene (NHC) ligands, which, like dinitrogen, are weak electron acceptors. Consistent with prior studies, we found that when electrons are loaded into the [Fe_4_S_4_] clusters, the excess electron density resides mostly on the iron and sulfide ions. On the other hand, electron loading into the [MoFe_3_S_4_] clusters results in attenuated changes at the iron, sulfide, and molybdenum ions; instead, the iron ions in the [MoFe_3_S_4_] clusters—but not the [Fe_4_S_4_] clusters—deliver substantial electron density to the weakly accepting NHCs. The chemical basis for this difference is discussed. Insofar as these findings translate to the dinitrogen chemistry of nitrogenase co-factors, they may explain the greater efficiency of the molybdenum nitrogenase and reveal a principle for designing dinitrogen reduction catalysts: large metals such as molybdenum make neighboring iron ions more competent at transferring electron density to weakly accepting ligands.

## In brief

Why is the Mo nitrogenase more adept at N_2_ reduction than its alternative nitrogenase counterparts (V and Fe-only)? To investigate this question, we undertook a comparative structural and spectroscopic analysis of synthetic [MoFe_3_S_4_] and [Fe_4_S_4_] clusters. We found differences in how the clusters distribute electron density, with the iron centers in the [MoFe_3_S_4_] clusters being better at backbonding to weakly π-acidic ligands. Such differences may account for the greater efficiency of the Mo nitrogenase.

## Graphical Abstract

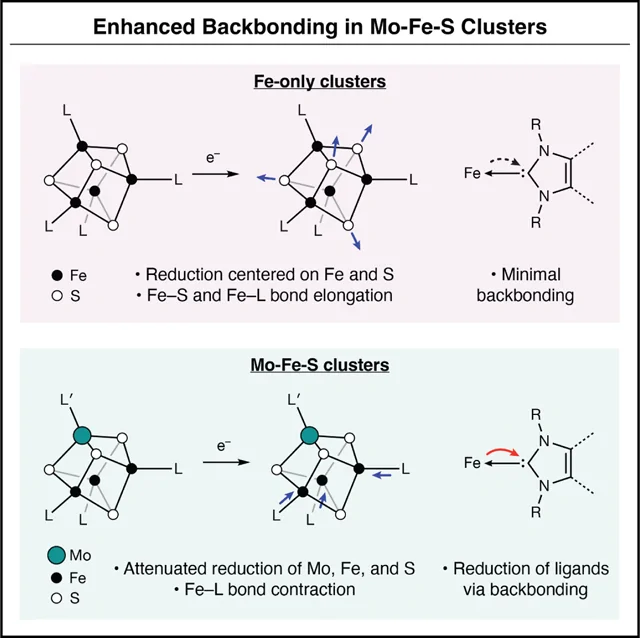

## INTRODUCTION

**Figure 1.**
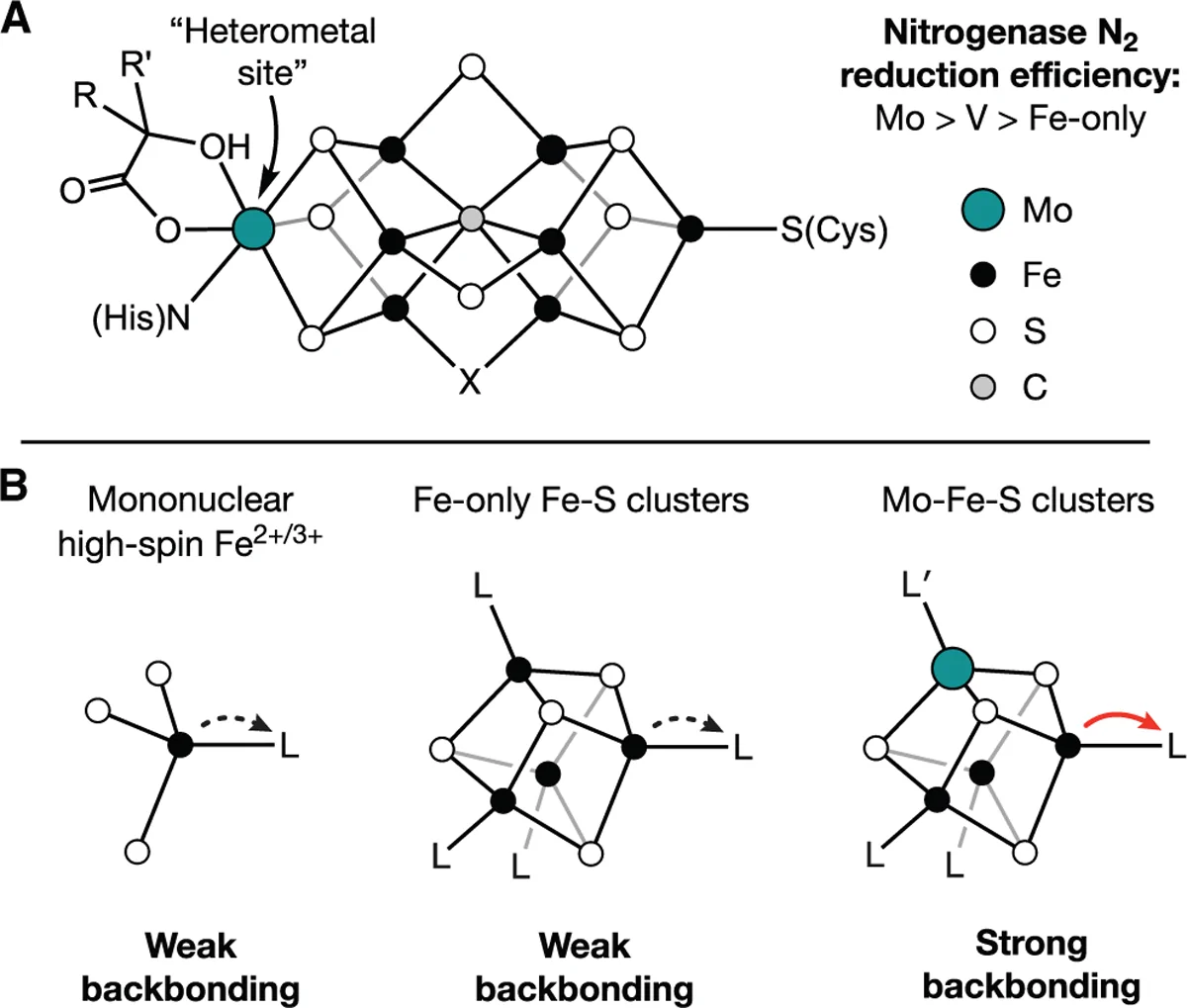
Hypothesis concerning how Mo incorporation impacts nitrogenase catalysis and backbonding at neighboring Fe sites in Fe-S clusters (A) Depiction of the nitrogenase cofactors. X = S^2−^ for FeMo-co and FeFe-co, and CO_3_^2−^ for FeV-co; R = CH_2_CH_2_CO_2_^−^; R^′^ = CH_2_CO_2_^−^. (B) This work: enhanced backbonding to weak π-acids at Fe centers in [MoFe_3_S_4_] clusters compared with [Fe_4_S_4_] clusters.

Nitrogenases are the sole biocatalysts that convert the most abundant form of nitrogen, N_2_, into bioavailable NH_3_.^1^ Of the three nitrogenase isozymes (the Mo, V, and Fe-only nitrogenases),^1^ the Mo nitrogenase directs the greatest portion of its electron flux to N_2_ reduction while minimizing off-path H^+^ reduction.^2–7^ As a result, the Mo nitrogenase is preferentially expressed in Mo-replete environments,^6,8,9^ although all three are important contributors to the global nitrogen cycle.^10–12^ The three isozymes also display divergent reactivity with non-native substrates.^13–18^ There has thus been interest in understanding the molecular-level basis for these differences in reactivity, particularly how the catalytic outcomes are influenced by the composition of the nitrogenase catalytic co-factors: FeMo-co, FeV-co, and FeFe-co, which differ primarily in the identity of the homocitrate-bound metal center (Figure 1).^1,4,19,20^

Of the three isozymes, the Mo nitrogenase was the first discovered^21,22^ and remains the most thoroughly studied.^23,24^ The proliferation of Mo-based N_2_ complexes and N_2_ reduction catalysts^25–29^ led to early hypotheses that Mo is the site of N_2_ binding.^30,31^ However, it is now generally thought that N_2_ coordination occurs at one or more Fe sites. Support for the latter hypothesis includes findings that (1) mutagenesis of residues proximal to the Fe sites perturbs substrate reduction,^6,18,32–37^ (2) CO coordinates to one or more Fe sites,^15,38,39^ (3) the belt sulfur (S) atoms are exchangeable,^40^ and (4) several states with potential non-S ligands bound to the belt Fe sites have been crystallographically characterized.^41–45^ The plausibility of N_2_ binding at one or more Fe sites in the nitrogenase co-factors has been additionally bolstered by model chemistry, including studies of Fe-N_2_ complexes,^46–49^ Fe-based catalysts for N_2_ reduction,^50,51^ and well-defined Mo-Fe-S clusters that bind and activate N_2_ at Fe.^52,53^

The emerging consensus that N_2_ binds at Fe has raised questions concerning the roles of Mo and V in nitrogen fixation: how do these “heterometals” change the electronic structure of an Fe-S cluster, and how do such changes manifest as differences in reactivity and catalytic outcomes? Comparative studies of FeMo-co and FeV-co, as well as synthetic analogs, have pointed to differences in the electron density distribution and degree of delocalization, with the diffuse nature of the Mo 4d orbitals leading to stronger Mo-Fe interactions compared to V-Fe interactions.^54–56^ The increased Mo-Fe orbital mixing in FeMo-co has also been proposed to lead to greater stability in turnover states with open coordination sites and/or weakly coordinating ligands.^54,57^ However, a complete picture of how the presence of Mo (or V) in nitrogenase catalytic co-factors contributes to differences in reactivity—particularly with respect to binding and activating π-acidic substrates—remains lacking.

We herein address this question through the synthesis and characterization of multiple redox states of cuboidal [MoFe_3_S_4_] and [Fe_4_S_4_] clusters. Our analysis reveals that the structural and spectroscopic trends observed for the [Fe_4_S_4_] clusters—wherein electron loading occurs mostly at Fe and S, as is typically seen for Fe-S clusters—are not observed for the [MoFe_3_S_4_] clusters, and we attribute these differences to the [MoFe_3_S_4_] clusters being more adept at backbonding at their Fe sites. The consequences of these findings for N_2_ reduction in nitrogenases are discussed.

## RESULTS

### Synthesis and infrared spectroscopic characterization

The clusters studied herein can be sorted into four groups, each containing members in at least two molecular charges:

- [MoFe_3_S_4_] clusters of the form [Cp*MoFe_3_S_4_(NHC)_2_CO] in which Mo is bound by Cp* (Cp* = pentamethylcyclopentadienyl), two Fe centers are bound by an NHC (NHC = *N*-heterocyclic carbene), and the third Fe center is bound by CO: [**1**]^−/0/+^;
- [MoFe_3_S_4_] clusters that are structurally identical to those mentioned above, except all three Fe centers are bound by NHCs ([Cp*MoFe_3_S_4_(NHC)_3_]): [**2**]^0/+^;
- [Fe_4_S_4_] clusters of the form [Fe_4_S_4_(NHC)_3_CO] in which three Fe sites are bound by NHCs and the fourth is bound by CO: [**3**]^0/+^; and
- [Fe_4_S_4_(NHC)_4_] clusters that are fully ligated by NHCs: [**4**]^0/+^.

The [Fe_4_S_4_] clusters were prepared according to reported protocols,^58–60^ and the syntheses of the [MoFe_3_S_4_] clusters are described below. The structures of all clusters were analyzed by single-crystal X-ray diffraction (SC-XRD) analysis, discussion of which is provided in the structural analysis section.

**Figure 2.**
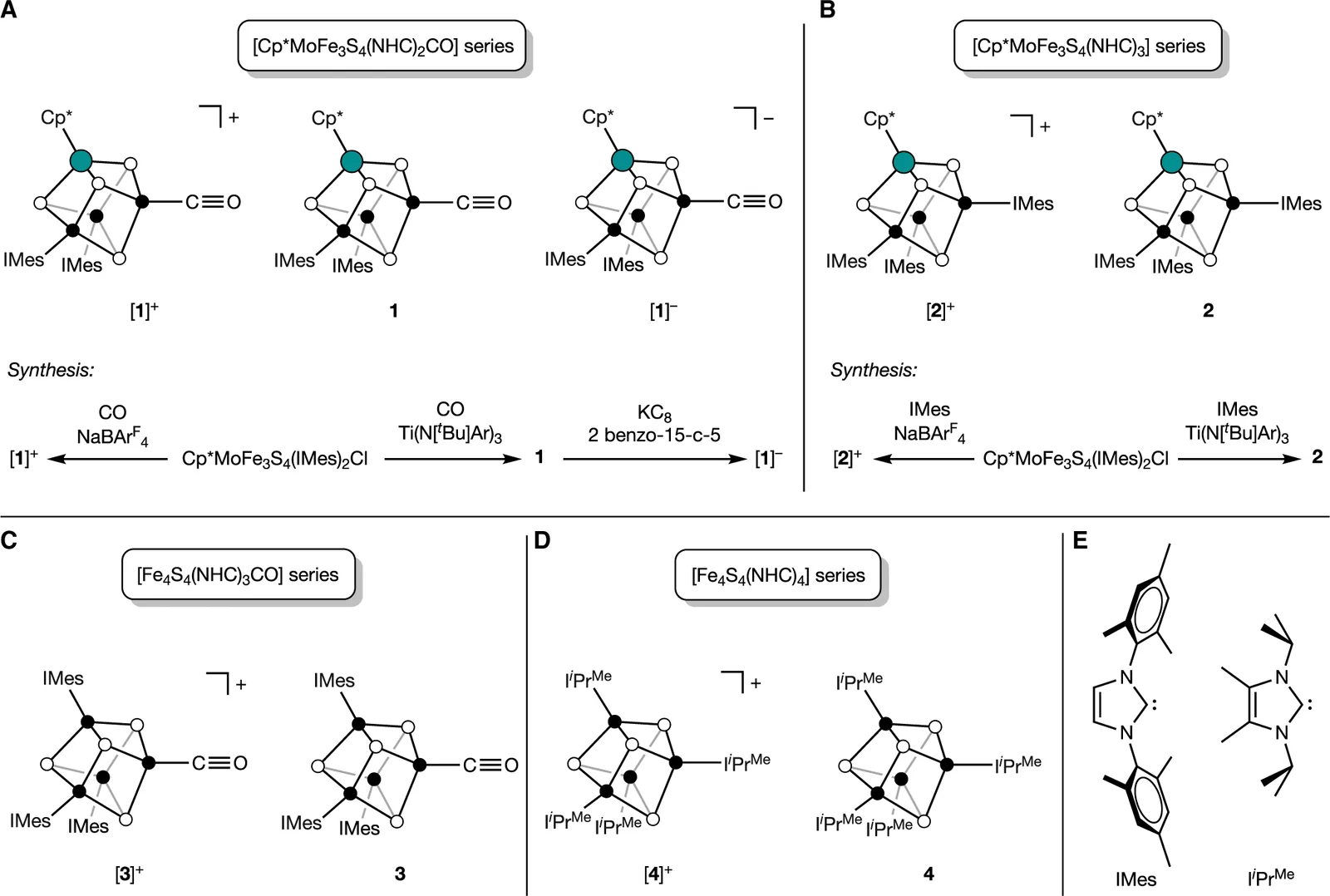
[MoFe_3_S_4_] and [Fe_4_S_4_] clusters employed in this study (A) Synthesis of CO-ligated [MoFe_3_S_4_] clusters. (B) Synthesis of fully NHC-ligated [MoFe_3_S_4_] clusters. (C) Previously reported CO-ligated [Fe_4_S_4_] clusters. (D) Previously reported fully NHC-ligated [Fe_4_S_4_] clusters. (E) NHCs utilized in this study. IMes, 1,3-bis(2,4,6-trimethylphenyl)imidazol-2-ylidene; I^*i*^Pr^Me^, 1,3-diisopropyl-4,5-dimethylimidazol-2-ylidene; Ar^F^_4_, 3,5-bis(trifluoromethyl)phenyl; Ar, 3,5-dimethylphenyl. Teal, black, and white balls represent Mo, Fe, and S atoms, respectively.

The [MoFe_3_S_4_]–CO clusters were synthesized by first imparting 2:1 site differentiation to the three Fe sites of an [MoFe_3_S_4_] cluster, followed by binding CO to the unique Fe site. Specifically, we prepared Cp*MoFe_3_S_4_(IMes)_2_Cl (IMes = 1,3-bis(2,4,6-trimethylphenyl)imidazol-2-ylidene) by treatment of [PPh_4_][Cp*MoFe_3_S_4_Cl_3_]^61^ with 2.2 equiv IMes and 1.1 equiv Cp_2_Co in tetrahydrofuran (THF); this protocol is similar to the previously reported preparation of Cp*MoFe_3_S_4_(IPr)_2_Cl (IPr = 1,3-bis(2,6-diisopropylphenyl)imidazol-2-ylidene).^52^ Abstraction of Cl^−^ from Cp*MoFe_3_S_4_(IMes)_2_Cl using Na[BAr^F^_4_] (Ar^F^ = 3,5-bis(trifluoromethyl)phenyl) in Et_2_O followed by treatment with CO yielded the cationic [MoFe_3_S_4_]^2+^ cluster [Cp*MoFe_3_S_4_(IMes)_2_CO][BAr^F^_4_] ([**1**][BAr^F^_4_], henceforth referred to as [**1**]^+^) (Figure 2A). The one-electron reduced cluster, Cp*MoFe_3_S_4_(IMes)_2_CO (**1**), was generated by Cl· abstraction from Cp*MoFe_3_S_4_(IMes)_2_Cl with Ti(N[^*t*^Bu]Ar)_3_ (Ar = 3,5-dimethylphenyl)^62^ in the presence of CO (Figure 2A). Cluster [**1**]^+^ has an *S* = 0 ground spin state as established by superconducting quantum interference device (SQUID) magnetometry (Figure S21), and **1** has an *S* =^1^/_2_ ground spin state as established by electron paramagnetic resonance (EPR) spectroscopy (Figure S14).

**Figure 3.**
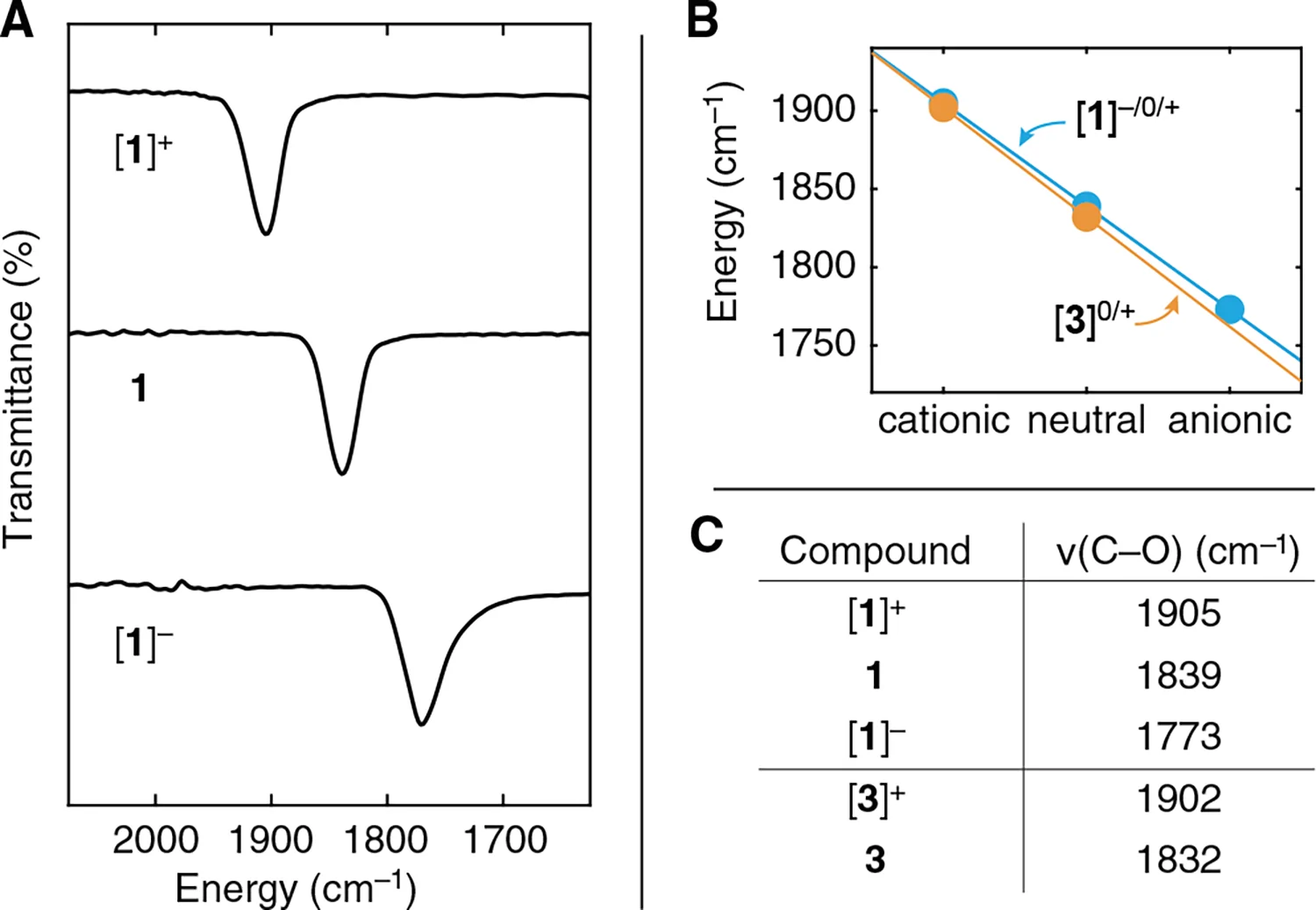
IR spectroscopic characterization of CO-bound clusters (A) IR spectra of [**1**]^−/0/+^. (B) Comparisons of C–O activation at [**1**]^−/0/+^and [**3**]^0/+^, showing the similarity between the two series. Values for [**3**]^+^ and **3** were reported in Brown et al.^58^ (C) Table of *ν*(C–O) values for [**1**]^−/0/+^and [**3**]^0/+^.

The infrared (IR) C–O stretching frequencies of **1** and [**1**]^+^ are 1,839 and 1,905 cm^−1^ (Figures 3, S8, and S9), respectively, and are strikingly similar to those of their [Fe_4_S_4_]^0^ and [Fe_4_S_4_]^+^ analogs: **3** and [**3**]^+^ (1,832 and 1,902 cm^−1^, respectively; Figure 3).^58^ Apparently, swapping a remote IMes-ligated Fe center for a Cp*-ligated Mo center in these clusters has little effect on the ability of the tetrahedral Fe_CO_ site to activate its CO ligand. For **3** and [**3**]^+^, it was shown that the high degree of C–O bond weakening is due to their Fe_CO_ sites having Fe^1+^ and/or intermediate-spin Fe^2+^ character, which enables full occupation of the Fe–CO backbonding orbitals^58,63^; similar conclusions were reached in [WFe_3_] and [MoFe_3_] clusters featuring carbonylated Fe sites.^64,65^ The comparable ν(C–O) values for **1** and **3** as well as for [**1**]^+^ and [**3**]^+^ suggest that the Fe_CO_ sites in these [MoFe_3_S_4_] clusters likewise feature fully occupied backbonding orbitals and some Fe^1+^ and/or intermediate-spin Fe^2+^ character.

The cyclic voltammogram of **1** shows a reversible wave centered at −1.39 V (vs. Fc/Fc^+^, compared with −1.54 V for **3**) corresponding to the **1**/[**1**]^+^ couple and, in contrast to **3**, a reversible reduction event (at −2.35 V; Figure S29). Treatment of **1** with 1.5 equiv KC_8_ and 2 equiv benzo-15-crown-5 in THF yielded the anionic [MoFe_3_S_4_]^0^ cluster [K(benzo-15-c-5)_2_][Cp*MoFe_3_S_4_(IMes)_2_CO] ([**1**]^−^; Figure 2A), which has a C–O stretching frequency of 1,773 cm^−1^, lower than that of **1** by 66 cm^−1^ (Figures 3 and S10). The ground spin state of [**1**]^−^ is *S* = 0, as determined by SQUID magnetometry (Figure S22). For comparison, the pair of clusters—neutral **1** and anionic [**1**]^−^ —displays slightly greater degrees of C–O activation than a pair of neutral/anionic [WFe_3_] clusters with terminal CO ligands (ν(C–O) = 1,851 and 1,782 cm^−1^, respectively).^64^

As discussed below, we observed unexpected differences in the structural and spectroscopic properties of the [MoFe_3_S_4_]–CO clusters compared with the [Fe_4_S_4_]–CO clusters, so we also prepared and studied [MoFe_3_S_4_] and [Fe_4_S_4_] clusters in which all Fe sites are ligated by NHCs, which, compared with CO, are much poorer π-acceptors.^66^ The more reduced [MoFe_3_S_4_] cluster Cp*MoFe_3_S_4_(IMes)_3_ (**2**) was synthesized by the addition of Ti(N[^*t*^Bu]Ar)_3_ and IMes to Cp*MoFe_3_S_4_(IMes)_2_Cl in benzene (Figure 2B). Its one-electron oxidized congener, [Cp*MoFe_3_S_4_(IMes)_3_][BAr^F^_4_] ([**2**][BAr^F^_4_]), was synthesized by treatment of Cp*MoFe_3_S_4_(IMes)_2_Cl with Na[BAr^F^_4_] and IMes in THF (Figure 2B). Cluster **2** has an *S* = ^1^/_2_ ground spin state, established by EPR spectroscopy (Figure S13), and [**2**]^+^ has an *S* = 2 ground spin state (typical for [MoFe_3_S_4_]^2+^ clusters),^67,68^ established by SQUID magnetometry (Figure S23). The analogous all-NHC-ligated [Fe_4_S_4_] clusters, [(I^*i*^Pr^Me^)_4_Fe_4_S_4_]^0/+^ ([**4**]^0/+^ [I^*i*^Pr^Me^ = 1,3-diisopropyl-4,5-dimethylimidazol-2-ylidene]), were prepared according to reported protocols.^59,60^

With the [**1**]^−/0/+^, [**2**]^0/+^, [**3**]^0/+^, and [**4**]^0/+^ series in hand, we next investigated how the spectroscopic and structural properties of the clusters are affected by the cluster composition (i.e., [MoFe_3_S_4_] for the [**1**]/[**2**] series vs. [Fe_4_S_4_] for the [**3**]/[**4**] series) and the coordination sphere (i.e., the heteroleptic coordination spheres of the [**1**] and [**3**] series vs. the all-NHC ligation in the [**2**] and [**4**] series).

### Mössbauer analysis

**Figure 4.**
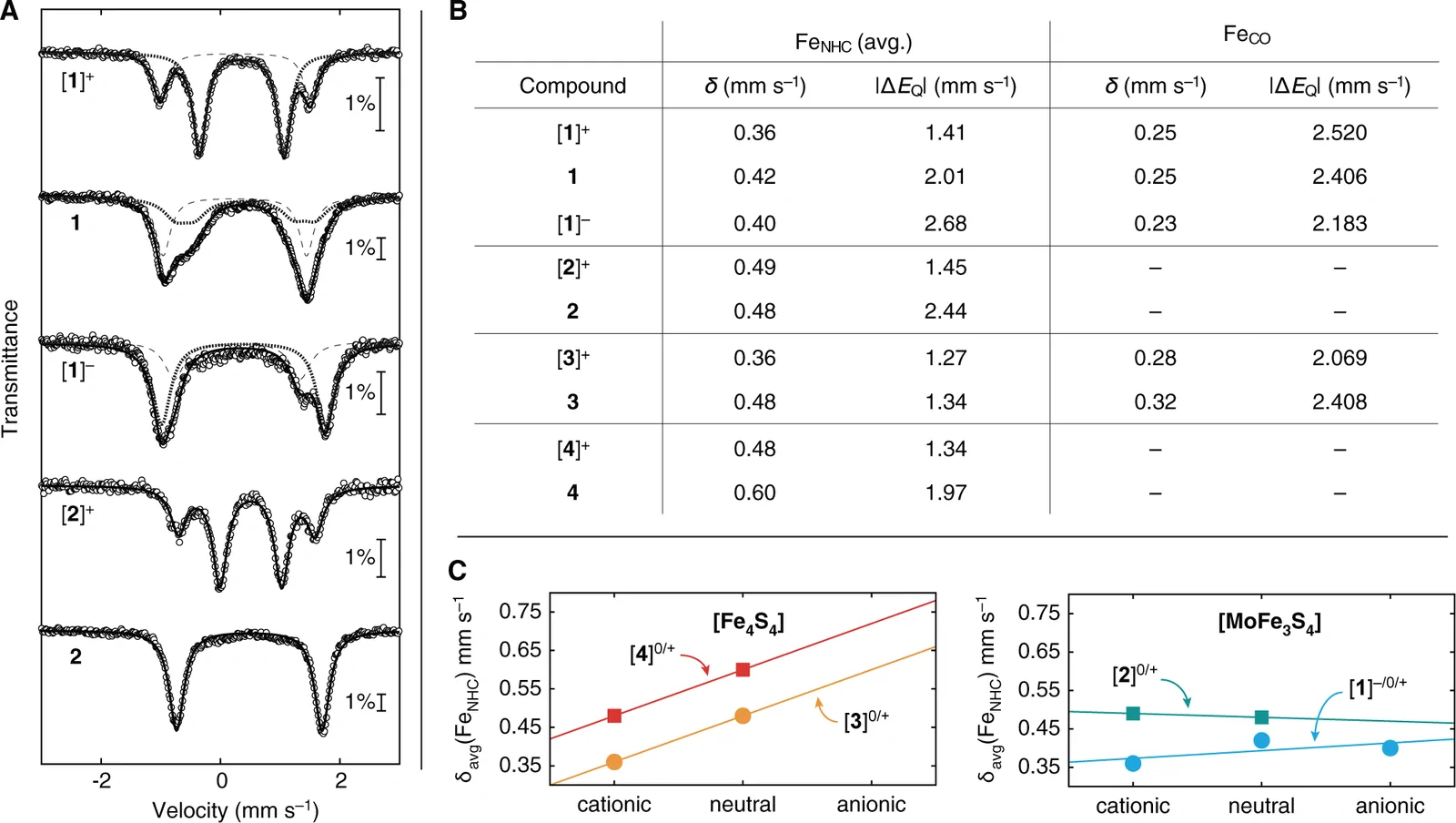
^57^Fe Mössbauer spectroscopic trends among [Fe_4_S_4_] and [MoFe_3_S_4_] clusters (A) Mössbauer spectra of [**1**]^+^ (top), **1** (second from top), [**1**]^−^ (middle), [**2**]^+^ (second from bottom), and **2** (bottom). Thick dashed black: the sum of the quadrupole doublets corresponding to the Fe_NHC_ sites; thin dashed black: the quadrupole doublets of the Fe_CO_ sites; solid black: the total simulation (equivalent to the thick dashed black traces for [**2**]^+^ and [**2**]). (B) Table of simulated Mössbauer parameters. Values for [**3**], [**3**]^+^, [**4**], and [**4**]^+^ were obtained from Brown et al.^58^ and Deng and Holm.^59^ (C) Plot of *δ*_avg_(Fe_NHC_) for the [Fe_4_S_4_] clusters (left), showing a typical relationship between isomer shift and molecular charge (and, by extension, average Fe oxidation state). Plot of *δ*_avg_(Fe_NHC_) for the [MoFe_3_S_4_] clusters (right), showing the unusual invariance of isomer shift with respect to molecular charge.

The 80 K ^57^Fe Mössbauer spectra of the [MoFe_3_S_4_] clusters are shown in Figure 4A; those of [**3**], [**3**]^+^, [**4**], and [**4**]^+^ were reported previously,^58,59^ and the relevant simulated parameters are supplied in Figure 4B. For most isostructural series of Fe-S clusters—and for [Fe_4_S_4_] clusters in particular—it is well established that the average Mössbauer isomer shift (*δ*_avg_) correlates approximately linearly with the average Fe oxidation state.^69^ For [Fe_4_S_4_] clusters, a ~0.1 mm s^−1^ change is expected for a one-electron redox event averaged over four sites.^69,70^ In analyzing the previously reported Mössbauer spectra of **3**/[**3**]^+^ and **4**/[**4**]^+^, we found that such trends are obeyed for the NHC-bound Fe sites (Fe_NHC_); upon oxidation of **3** to [**3**]^+^, a difference of −0.12 mm s^−1^ was observed for *δ*_avg_(Fe_NHC_) (with additional charge depletion at the CO-bound site), and the same magnitude change was observed for the four NHC-bound sites in **4**/[**4**]^+^ (Δ*δ*_avg_(Fe_NHC_) = −0.12 mm s^−1^). Thus, the changes in *δ*_avg_ for the two [Fe_4_S_4_] series follow the established trends.

The Mössbauer spectrum of [**1**]^+^ was simulated with two quadrupole doublets in a 2:1 ratio corresponding to the two Fe_NHC_ sites and the Fe_CO_ site, respectively. In the Mössbauer spectrum of **1**, the overlapping signals were simulated with three quadrupole doublets in a 1:1:1 ratio. The quadrupole doublet assigned to Fe_CO_ can be identified on the basis of its larger |Δ*Е*_Q_| and lower *δ*, as have been previously found for Fe_CO_ and related sites in Fe-S clusters.^58,63^ The Mössbauer spectrum of [**1**]^−^ was simulated using three quadrupole doublets in a 1:1:1 ratio. Several simulations for the spectrum were considered, each of which features one quadrupole doublet with a low isomer shift (~0.2 mm s^−1^) and two quadrupole doublets with higher isomer shifts (~0.4 mm s^−1^). The quadrupole doublet with the lowest isomer shift was assigned to the CO-bound site because such a low isomer shift (*δ* = 0.23 mm s^−1^ in our preferred simulation) is unreasonable for a site in a relatively reduced Fe-S cluster that is not bound to a strongly π-accepting ligand. With these simulations, as well as the Mössbauer data for **2** and [**2**]^+^, we could evaluate how *δ*_avg_(Fe_NHC_) varies across two [MoFe_3_S_4_] redox series.

Interestingly—and in contrast with the [Fe_4_S_4_] clusters—there was little change in *δ*_avg_(Fe_NHC_) as a function of cluster charge state for the [MoFe_3_S_4_] clusters (Figure 4C). A nearly identical *δ*_avg_(Fe_NHC_) value was observed for **2** and [**2**]^+^ (Figure 4B), and *δ*_avg_(Fe_NHC_) similarly varied little across the [**1**]^−/0/+^ series. To explain the minimal change in *δ*_avg_(Fe_NHC_) for the two series of [MoFe_3_S_4_] clusters, we initially considered the possibilities that either (1) the Mo center is the primary locus of redox chemistry, or (2) in the [**1**]^−/0/+^ series, the redox events are centered at the Fe_CO_ site. We rule out (1) in the next section. We also rule out (2) because the changes in ν(C–O) across the redox series of CO-bound clusters ([**1**]^−/0/+^ and [**3**]^0/+^) are comparable (ca. 70 cm^−1^ per electron oxidation), which is inconsistent with redox events being more localized at the Fe_CO_ site in the [**1**]^−/0/+^ series than in the [**3**]^0/+^ series. As discussed below, we ultimately attribute the small changes in *δ*_avg_(Fe_NHC_) to differences in π-backbonding with the NHC ligands in the [MoFe_3_S_4_] clusters, compared with that in the [Fe_4_S_4_] clusters.

Despite the apparent lack of change in *δ*_avg_(Fe_NHC_) within the two redox series of [MoFe_3_S_4_] clusters, |Δ*Е*_Q_|_avg_(Fe_NHC_) is somewhat more responsive to the change in oxidation state, decreasing by 1.27 mm s^−1^ over the series [**1**] ^−/0/+^ (avg. decrease of 0.64 mm s^−1^ per oxidation event) and 0.99 mm s^−1^ from **2** to [**2**]^+^. In comparison, the changes in |Δ*Е*_Q_|_avg_(Fe_NHC_) for the [Fe_4_S_4_] clusters are smaller: it decreases by 0.63 mm s^−1^ from **4** to [**4**]^+^ but only by 0.07 mm s^−1^ from **3** to [**3**]^+^. This, too, is consistent with more dramatic changes in π-backbonding for the [MoFe_3_S_4_] clusters because of the strong effect of π-bonding on the local electric field gradient and, therefore, on |Δ*Е*_Q_|_avg_(Fe_NHC_).

### X-ray absorption spectroscopy

The lack of change in *δ*_avg_(Fe_NHC_) for the [MoFe_3_S_4_] clusters could be interpreted as redox events not being localized to the Fe sites. Therefore, we turned to X-ray absorption spectroscopy (XAS), specifically Mo K-edge HERFD-XAS (high-energy-resolution fluorescence-detected XAS) as well as Fe and S K-edge XAS, to assess if the redox events in the series [**1**]^−/0/+^ and [**2**]^0/+^ could be occurring primarily on the Mo or S centers. We first discuss the Fe and S K-edge XAS spectra for the [Fe_4_S_4_] complexes **3** and [**4**]^0/+^. Note that collection of XAS spectra for [**3**]^+^ was precluded by its instability under vacuum and thus our inability to prepare sufficient quantities of pure sample for XAS analysis.

In Fe K-edge XAS, the Fe pre-edge (1s → 3d) and rising edge (1s → 4p/continuum) energies are often used to evaluate Fe oxidation and spin state, although for Fe-S clusters conflicting trends have been reported. For example, a recent analysis of a five-member thiolate-ligated series of [Fe_4_S_4_]^n+^ clusters spanning n = 0–4 identified a ca. 0.6 eV blueshift in the first inflection point of the Fe K rising edge attending each oxidation of the cluster^71^; similar changes have been reported for ferredoxins.^72^ These results satisfy the convention of K-edge blueshifts following metal-centered oxidation. In other cases, such shifts are attenuated or even not observed.^73,74^ For instance, studies of benzimidazole-supported [Fe_2_S_2_]^n−^ clusters with n = 1–3 indicated that the rising edges can be influenced by the spin coupling and, in mixed-valent clusters, can be dominated by the edge of the most reduced Fe ion.^75^

**Figure 5.**
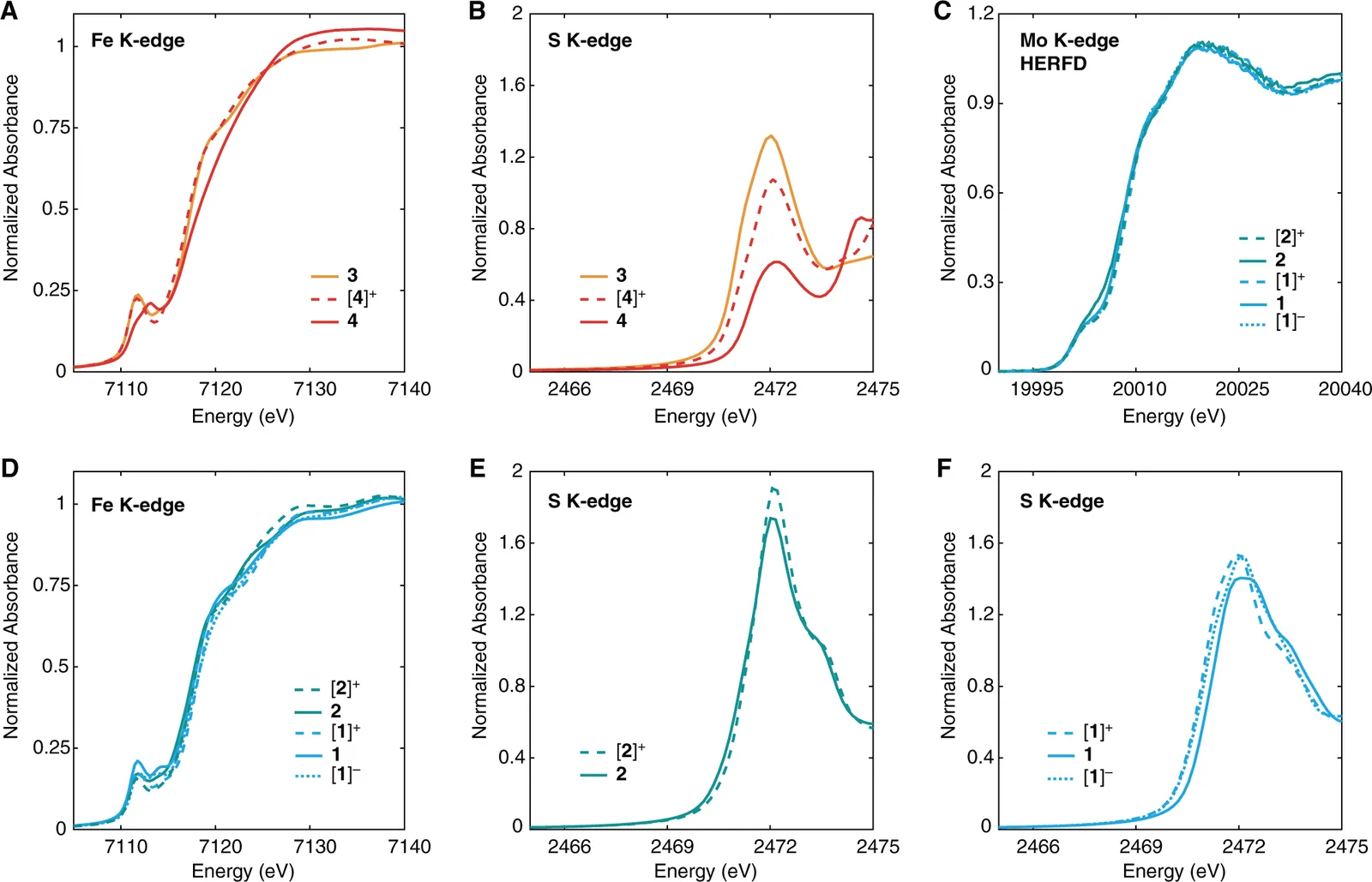
XAS data (A) Fe K-edge spectra of **3**, [**4**]^+^, and **4**. (B) S K-edge spectra of **3**, [**4**]^+^, and **4**. (C) Mo K-edge HERFD spectra of [**2**]^+^, **2**, [**1**]^+^, **1**, and [**1**]^−^. (D) Fe K-edge spectra of [**2**]^+^, **2**, [**1**]^+^, **1**, and [**1**]^−^. (E) S K-edge spectra of [**2**]^+^ and **2**. (F) S K-edge spectra of [**1**]^+^, **1**, and [**1**]^−^.

Given the observations described above, directly relating Fe K-edge energies to oxidation state in multimetallic clusters (including but not limited to Fe-S clusters) can be challenging due to both the overlapping edges from multiple metal sites as well as from potential metal-metal interactions that perturb the energy levels of unoccupied orbitals.^72–75^ Comparisons of the three [Fe_4_S_4_] clusters studied herein (**3**, **4**, and [**4**]^+^) further showcase the complexities of interpreting the XAS spectra across cluster charge states. The Fe K-edge XAS spectrum of **4** shows two pre-edge features at 7,111.4 and 7,113.2 eV and a rising edge at 7,116.8 eV (Figure 5A; Table 1). Oxidation of **4** to [**4**]^+^ results in a change in the pre-edge to a single feature at 7,111.4 eV; the rising edge remains constant at 7,116.8 eV. That there is no change in the rising edge energy contradicts simple trends in rising edge energies with oxidation state^76^ but would be consistent with the contributions from Fe^2+^ dominating the rising edge position. The white line intensity in Fe K-edge XAS at 7,125 eV has previously been observed to decrease upon oxidation of Fe-S clusters; the origin of this trend is not yet understood.^72,73,75^ We note, however, that there is no difference in the white line intensity for **4** and [**4**]^+^ at 7,125 eV. Cluster **3** has a nearly identical Fe K-edge spectrum to [**4**]^+^, with only a single pre-edge feature at 7,111.5 eV (Table 1). Overall, because of the presence of multiple Fe ions in multiple oxidation states, it is difficult to develop a simple, molecular-level model that accounts for the rich features in the Fe K-edge spectra of these [Fe_4_S_4_] clusters.

S K-edge XAS spectroscopy has been previously demonstrated to be an excellent marker of cluster core charge state and Fe-S mixing in Fe-S clusters.^77^ In particular, the intensity of the pre-edge (1s → 3p) feature can be directly related to the S 3p character in the d-manifold and therefore to the covalency of the M–S bonds.^78,79^ It has been shown that oxidation is accompanied by an increase in M–S mixing, leading to an increase in the integrated area of the pre-edge feature in the S K-edge spectra.^78^ In the case of Fe-S clusters, S K-edge XAS studies have shown that thiolate-supported [Fe_4_S_4_] clusters exhibit changes in Fe–S mixing with oxidation, which can be related to changes in the percentage S 3p contributions to frontier unoccupied orbitals.^80^

As expected, substantial changes in the S K-edge XAS data are observed across the range of [Fe_4_S_4_] clusters studied herein (Figure 5B). The intensity of the pre-edge feature in the S K-edge spectrum of **4** is relatively weak (1.00(1) integrated area), corresponding to 15% S 3p character per Fe–S bond. Upon oxidation to [**4**]^+^, the intensity of the pre-edge feature increases dramatically to 1.62(1) integrated area, corresponding to 25% S 3p. Both **4** and [**4**]^+^ have lower Fe-S mixing than reported for [Fe_4_S_4_Cl_4_]^2−^ ([Fe_4_S_4_]^2+^ cluster, 39% S 3p),^78^ consistent with their more reduced core charges ([Fe_4_S_4_]^0^ and [Fe_4_S_4_]^1+^). In **3**, the intensity of the pre-edge feature is even greater at 2.10(1) integrated area (32% S 3p), reflecting the electron-deficiency of the Fe sites (manifested as increased Fe-S mixing) due to the highly electron-withdrawing CO ligand. This observation is consistent with the Mössbauer data (*vide supra*) as well as the X-ray crystallographic structure of **3** (*vide infra*), and highlights how Fe–CO backbonding is ultimately supported by S-to-Fe donation throughout the cluster.

Having demonstrated that either increasing the oxidation state or coordinating an electron-withdrawing CO ligand to the [Fe_4_S_4_] clusters leads to the expected changes in the S K-edge XAS spectra, we next analyzed the [MoFe_3_S_4_] clusters. Examination of the HERFD Mo K-edge data reveals that all five clusters ([**1**]^−/0/+^ and [**2**]^0/+^) have nearly superimposable spectra (Figure 5C; Table 1), characterized by a weak pre-edge feature between 20,002.0 and 20,002.3 eV (the weakness being consistent with a pseudo-octahedral coordination environment) and a rising edge from 20,008.0 to 20,009.0 eV. These features are highly similar to those observed for [TpMoFe_3_S_4_Cl_3_]^−^, for which the Mo HERFD-XAS shows a rising edge at 20,009.1 eV and a weak pre-edge feature at 20,002.1 eV.^81^ The nearly identical Mo K-edge HERFD-XAS spectra for [MoFe_3_S_4_] clusters across four charge states—from [MoFe_3_S_4_]^3+^ in [TpMoFe_3_S_4_Cl_3_]^−^ to [MoFe_3_S_4_]^0^ in [**1**]^−^ —suggest that Mo is in approximately the same oxidation state in each [MoFe_3_S_4_] cluster.

There are minor differences in the Fe K-edge XAS spectra between the five [MoFe_3_S_4_] clusters: the rising edge energies are very similar across redox states, and the energy of the rising edge increases slightly with increasing oxidation state (Table 1; Figure 5D). Similarly to **4** and [**4**]^+^, the white line intensity at 7,125 eV does not appear to trend with oxidation state for either the [**1**]^−/0/+^ series or the [**2**]^0/+^ series. Due to the difficulty in interpreting the Fe XAS spectra of multimetallic Fe complexes (*vide supra*), we do not further comment on the Fe XAS spectra.

The S K-edge spectra of **2** and [**2**]^+^ are, perhaps surprisingly, nearly superimposable (Figure 5E), indicating essentially no change in M–S (M = Fe and Mo) mixing upon oxidation of **2** to [**2**]^+^. In contrast, oxidation of the all-NHC-ligated [Fe_4_S_4_] cluster **4** to [**4**]^+^ results in an increase in Fe–S mixing of 10% (*vide supra*). The S K-edge pre-edge intensities (3.5(1) and 3.56(4) integrated area, respectively) are significantly higher than seen for the [Fe_4_S_4_] compounds (Table 1; Figure 5E), although direct comparisons are difficult since the pre-edge reflects both Mo–S and Fe–S covalency. Turning to the [MoFe_3_S_4_]–CO clusters [**1**]^−/0/+^, the S K-edge spectra are again nearly superimposable, and all three complexes have similar pre-edge intensities to one another and to **2** and [**2**]^+^ (integrated areas of 3.59(1) for [**1**]^−^, 3.79(4) for **1**, and 3.6(1) for [**1**]^+^; Table 1; Figure 5F). This is again in contrast to the spectra of the [Fe_4_S_4_] clusters, which exhibit more variable pre-edge intensities as a function of both charge state and CO ligation (*vide supra*, Table 1).

At this point, we can synthesize the information gleaned from the Mössbauer and Mo/Fe/S XAS spectra presented herein. Within an isostructural redox series, the [MoFe_3_S_4_] clusters show greatly attenuated spectroscopic changes compared with the [Fe_4_S_4_] clusters: upon oxidation of [**1**]^−^ to **1** to [**1**]^+^ or of **2** to [**2**]^+^, there is little change in *δ*_avg_(Fe_NHC_), and essentially no change in the S K-edge or Mo K-edge spectra. Overall, it appears that reduction of the [MoFe_3_S_4_] clusters does not lead to the major spectroscopic changes that are otherwise well documented for [Fe_4_S_4_] clusters. We next analyze the structures of the clusters to shed additional light on the differences in redox loading of [MoFe_3_S_4_] and [Fe_4_S_4_] clusters.

### Structural analysis

**Figure 6.**
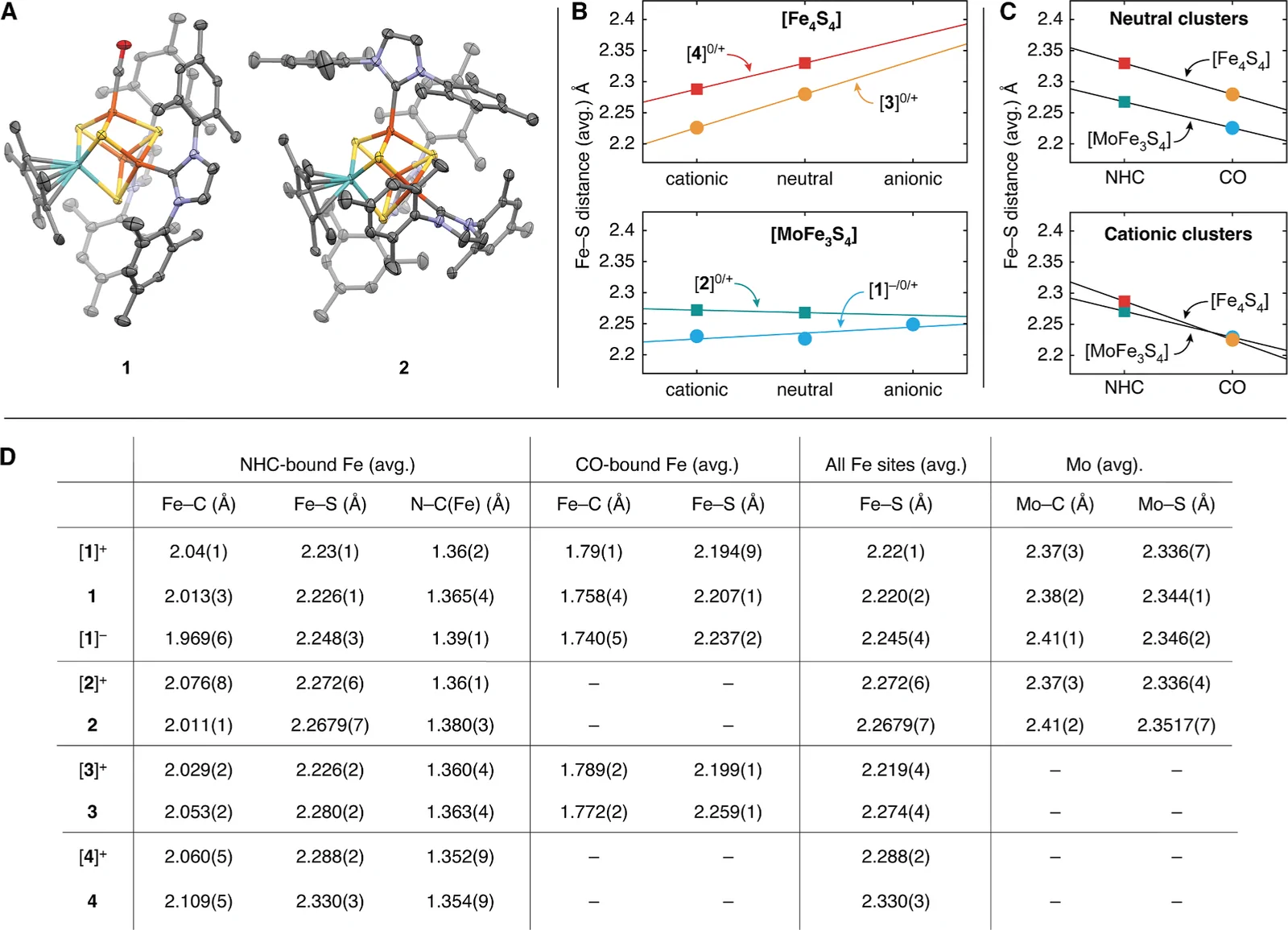
Structural comparisons of [Fe_4_S_4_] and [MoFe_3_S_4_] clusters (A) Crystallographic structures of **1** (left) and **2** (right) with ellipsoids at the 50% probability level. C (gray), N (blue), Fe (orange), S (yellow), Mo (teal), and O (red). The structures of [**1**]^+^, [**1**]^−^, and [**2**]^+^ are qualitatively similar and are shown in the Figures S31–S35. (B) Plots showing how the average Fe–S distances change upon oxidation for the [Fe_4_S_4_] clusters ([**3**]^0/+^ and [**4**]^0/+^, top) but are relatively invariant as a function of charge for the [MoFe_3_S_4_] clusters ([**1**]^−/0/+^ and [**2**]^0/+^, bottom). (C) Plots showing how replacing an NHC for CO results in a similar decrease in the average Fe–S distances for both the [Fe_4_S_4_] and [MoFe_3_S_4_] clusters in two charges. (D) Table of structural parameters. Values for **3**, [**3**]^+^, **4**, and [**4**]^+^ were obtained from Brown et al.^58^ and Deng and Holm.^59^

Structures of the five new compounds, [**1**]^−/0/+^ and [**2**]^0/+^, were obtained by SC-XRD (representative structures are shown in Figure 6A, and the remainder are shown in Figures S31–S35). What follows in this section is a comparative analysis of these structures as well as those of [**3**]^0/+^ and [**4**]^0/+^, which were previously reported.^58,59^

We first note that the structural differences within the series of [Fe_4_S_4_] clusters ([**3**]^0/+^ and [**4**]^0/+^) are as expected^69^: reduction leads to longer average Fe–S distances (Figures 6B–6D). For example, the reduced cluster **3** has longer Fe–S distances by an average of 0.054 Å (2.274(4) vs. 2.219(4) Å), compared with [**3**]^+^, and the average Fe–S distance in **4** (2.330(3) Å) is longer by a similar magnitude (0.042 Å), compared with [**4**]^+^ (2.288(2) Å). Such trends are broadly consistent with previous studies of Fe-S clusters^69,82^ and are consistent with the Mössbauer and XAS data that show a decrease in Fe–S mixing upon reduction.

Strikingly, within each [MoFe_3_S_4_] cluster series, [**1**]^−/0/+^ and **2**^0/+^, there is essentially no change in the average Fe–S distance (Figures 6B–6D). In fact, the average Fe–S distances for **2** and [**2**]^+^ are indistinguishable (2.2679(7) and 2.272(6) Å, respectively). Additionally, the Mo–S and Mo–C bonds change very little (Figure 6D), save for a modest contraction of the average Mo–C distance upon oxidation. The minimal structural changes at Mo are consistent with the Mo HERFD-XAS data and with what has been observed in structural comparisons of the few other examples of isostructural redox series of [MoFe_3_S_4_] clusters.^83,84^ Regarding the latter, it appears that changes in structural metrics for isostructural [MoFe_3_S_4_] clusters are generally smaller in magnitude than reported for typical [Fe_4_S_4_] clusters (Table S3). For example, comparing [TpMoFe_3_S_4_Cl_3_]^2−^ and [TpMoFe_3_S_4_Cl_3_]^−^, the average Fe–S distance decreases by only 0.018 Å upon oxidation (from 2.282(2) to 2.264(2) Å).^83^The magnitude of such changes is even smaller in our [MoFe_3_S_4_] clusters.

Given the typical structural and spectroscopic behavior for the series of the [Fe_4_S_4_] clusters and the general lack of change within the series of the [MoFe_3_S_4_] clusters, we considered the possibility that the two classes of clusters have different proclivities for backbonding with their NHC ligands. If there is indeed stronger Fe–NHC backbonding in the [MoFe_3_S_4_] clusters, this could explain, at least in part, the apparent lack of redox changes at the cluster; stronger Fe–NHC backbonding would enable the NHCs to buffer redox changes, reducing overall changes in the charges at the metal sites within the cluster.

**Figure 7.**
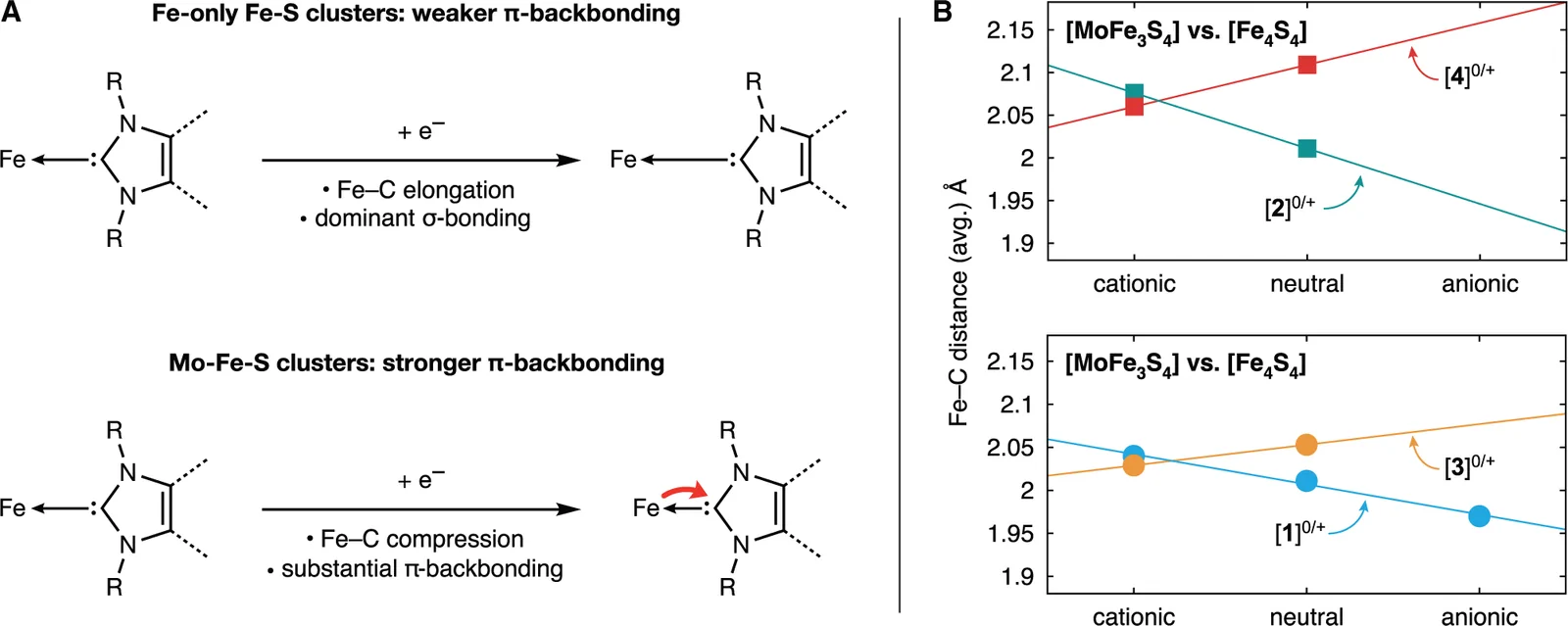
Differences in Fe–NHC bonding between Fe-only Fe-S clusters and Mo-Fe-S clusters (A) For Fe-NHC complexes featuring dominant σ-bonding Fe-S clusters, reduction leads to Fe–C elongation. In contrast, when π-backbonding is substantial, as in Mo-Fe-S clusters, reduction can lead to Fe–C compression, which allows for stronger π(Fe(3d)–C(2p)) overlap. (B) Plots of average Fe–C distance as a function of molecular charge for both the CO-ligated (top) and fully NHC-ligated (bottom) demonstrate substantial Fe–NHC π-backbonding for the [MoFe_3_S_4_] clusters and, in contrast, dominant σ-bonding for the [Fe_4_S_4_] clusters.

To assess whether the Fe_NHC_ sites in the [Fe_4_S_4_] and [MoFe_3_S_4_] clusters engage in different degrees of backbonding with their NHC ligands, we analyzed how the Fe–C(NHC) distances change upon electron loading. In the limit of weak/negligible π-backbonding, the Fe–C(NHC) distance is expected to increase upon reduction because the NHC acts primarily as a donor ligand (Figure 7A, top). On the other hand, in the limit of strong π-backbonding, reduction is expected to shorten the Fe–C(NHC) distance to enhance π-overlap (Figure 7A, bottom), as has been observed in Fe complexes featuring π-accepting phosphine ligands.^85–87^

In the [Fe_4_S_4_] redox series, the average Fe–C(NHC) distance lengthens upon reduction (Figures 6D and 7B): by 0.049 Å from [**4**]^+^ to **4** and by 0.024 Å from [**3**]^+^ to **3**. The latter difference is somewhat attenuated owing to redox changes occurring at the Fe_CO_ site. Both trends indicate that for the two series of [Fe_4_S_4_] clusters, the NHC ligands act primarily as σ-donors, and this finding is consistent with NHCs being weak π-acceptors and mid-valent Fe being poor at π-backbonding (Figure 7A, top).

In contrast, the Fe–C(NHC) distances substantially *shorten* upon reduction of the [MoFe_3_S_4_] clusters. For cluster **2**, the average Fe–C(NHC) distance is shorter than that in [**2**]^+^ by 0.065 Å (Figures 6D and 7B). Similar trends are observed within the [**1**]^−/0/+^ series, although again the magnitude is lower because some redox changes occur at the Fe_CO_ site (Figure 7B). The shortening of the Fe–C(NHC) distances in the [MoFe_3_S_4_] clusters upon reduction indicates stronger bonding—driven by stronger π-backbonding between the Fe centers and the NHC ligands than in the [Fe_4_S_4_] clusters (Figure 7A, bottom). Importantly, the stronger Fe–C(NHC) backbonding upon reduction is further supported by analysis of the average N–C(Fe) distances in the imidazole-2-ylidene ring. Specifically, stronger backbonding lessens contributions from resonance structures with N–C(Fe) double-bond character, which is manifested in longer N–C(Fe) distances. Indeed, the average N–C(Fe) distance in **2** is 0.02 Å greater than in [**2**]^+^, whereas the average N–C(Fe) distances in the corresponding [Fe_4_S_4_] clusters (**4** and [**4**]^+^) are identical (Figure 6D).

Overall, the divergent properties of the [Fe_4_S_4_] and [MoFe_3_S_4_] clusters studied herein can be attributed to differences in their ability to backbond to their weakly π-acidic NHC ligands. For the [MoFe_3_S_4_] clusters, the observations of increased backbonding accompanied by attenuated changes in Mössbauer isomer shifts are reminiscent of low-valent, mononuclear Fe chemistry, where such phenomena have been attributed to stronger Fe–L π-backbonding upon reduction.^86–90^

## DISCUSSION

Taken together, the results presented above indicate that compared with Fe-only Fe-S clusters, Mo-Fe-S clusters are more adept at backbonding with weak π-acids such as NHCs. The electron density employed in this backbonding ultimately derives from the sulfide ions, which accounts for the consistently short M–S (M = Mo/Fe) bonds in even the most reduced forms of the Mo-Fe-S clusters. But why are Mo-Fe-S clusters particularly adept at transferring electron density from sulfide ions to Fe centers and, ultimately, to acceptor orbitals on substrates?

Our explanation centers on the stronger M–Fe and M–S interactions in the [MoFe_3_S_4_] clusters, which are a result of the larger and more diffuse d-orbitals of Mo, compared with those of Fe. Typically, [Fe_4_S_4_] clusters containing tetrahedral Fe sites are described using an exchange-coupled framework with the metal ions interacting primarily via sulfide-mediated superexchange coupling (as well as double exchange within mixed-valent Fe^2+^/Fe^3+^ pairs). At another extreme, Mo-S clusters containing only Mo have been shown to exhibit substantial M–M bonding.^91^ Substantial Fe–Fe bonding has also been observed for clusters of high-spin Fe ions that feature very short Fe–Fe distances (ca. 2.3 Å).^92^ In line with other proposals,^54,64,81,93^ we suggest that [MoFe_3_S_4_] and related clusters may straddle these two regimes, with stronger M–Fe delocalization than is typical for Fe-only Fe-S clusters. Regardless of whether such enhanced delocalization is mediated primarily via the sulfides or direct M–Fe interactions, the functional consequence of Mo doping into Fe-S clusters could be to enable every M/S ion to donate electron density to the Fe center(s) bound to a π-acidic ligand.

The enhanced π-backbonding abilities of the Fe centers in Mo-Fe-S clusters are likely a confluence of multiple orbital interactions. One such interaction can be described as a filled Mo-based orbital donating into the filled Fe-based orbital that engages in backbonding with the NHC acceptor orbital. Such M–Fe–L(π*) mixing was observed computationally in [Fe_4_S_4_] clusters bound to CO and isocyanide ligands.^63^ Additionally or alternatively, an unfilled Mo-based orbital could accept electron density from a filled Fe–NHC σ* orbital, thereby strengthening Fe–NHC σ-bonding, which would lead to shorter Fe–C distances and in turn enhance Fe–NHC π-backbonding. Such multicenter σ-type interactions were observed computationally in a W-Fe-S cluster with CO bound at Fe.^64^ Although the localized orbital analysis in these prior studies point to the possibility of direct M–Fe–L orbital mixing, such interactions could be mediated primarily via the sulfides. Regardless, mixing of filled and/or unfilled Mo-based orbitals into π(Fe–NHC) and/or σ*(Fe–NHC) orbitals may synergistically make the Fe centers more adept at backbonding with π-acidic ligands.

It should be emphasized that the [MoFe_3_S_4_] clusters are differentiated from their Fe-only counterparts in their ability to backbond to *weak* π-acids. Indeed, when comparing the ability of these clusters to backbond to the strong π-acid CO, we observe a similar degree of C–O bond weakening for [MoFe_3_S_4_] and [Fe_4_S_4_] clusters with the same molecular charge. This can be attributed to the exceptionally strong π-accepting properties of CO, which lead to full occupation of the Fe_CO_ π-backbonding orbitals and maximal Fe_CO_–CO mixing. The fact that the Fe centers in the [MoFe_3_S_4_] clusters, but not in the [Fe_4_S_4_] clusters, engage in meaningful backbonding with the more weakly π-accepting NHC ligands suggests that the presence of Mo in Fe-S clusters might enable neighboring Fe centers to π-backbond and therefore bind more strongly to a wider range of π acids: here, NHCs, and perhaps N_2_ for FeMo-co. Such a proposal may in part account for the higher N_2_/H^+^ selectivity of the Mo nitrogenase compared with the alternative V and Fe-only nitrogenases,^2,4,7,19^ as well as the greater affinity for synthetic [MoFe_3_S_4_] clusters for N_2_ compared with isostructural [VFe_3_S_4_] and [Fe_4_S_4_] clusters.^56^

In summary, we reported two series of [MoFe_3_S_4_] clusters that, in comparison with analogous [Fe_4_S_4_] clusters, show higher proclivity for backbonding to the Fe centers’ weakly π-acidic NHC ligands. For the more reduced [MoFe_3_S_4_] clusters, the accumulation of electron density in the Fe–NHC backbonding orbitals attenuates the buildup of electron density elsewhere in the cluster, and this results in unusually muted spectroscopic and structural changes within each [MoFe_3_S_4_] redox series. The stronger backbonding by the Fe centers in the [MoFe_3_S_4_] clusters can be attributed to the enhanced M–S mixing and M–Fe delocalization imparted by the Mo ion. Such electronic effects may explain why, of the three nitrogenase isozymes, the Mo nitrogenase shows the highest efficiency for N_2_ reduction.

## METHODS

All reactions were performed using standard Schlenk techniques or in an LC Technologies inert-atmosphere glovebox under an N_2_ atmosphere. Chemicals were purchased from commercial suppliers and used as received. Details of routine preparation of materials and solvents are provided in the supplemental information. (I^*i*^Pr^Me^)_4_Fe_4_S_4_,^59^ [(I^*i*^Pr^Me^)_4_Fe_4_S_4_][BPh_4_],^60^ (IMes)_3_Fe_4_S_4_CO,^58^ IMes,^94,95^ Ti(N(^*t*^Bu)Ar)_3_,^62^ Na[BAr^F^_4_]^96^, K(napthalenide)·(THF)_0.5_,^97^ and [PPh_4_][Cp*MoFe_3_S_4_Cl_3_]^61^ were prepared according to literature procedures. Supplemental methods and other characterization data (NMR, IR, EPR and Mössbauer spectroscopy, XRD; Figures S1–S35; Tables S1–S3) are provided in the supplemental information.

### Synthetic methods

#### Cp*MoFe_3_S_4_(IMes)_2_Cl

Cp*MoFe_3_S_4_(IMes)_2_Cl was prepared using a procedure adapted from that employed for the synthesis of Cp*MoFe_3_S_4_(IPr)_2_Cl.^52^ A solution of IMes (687 mg, 2.26 mmol, 2.2 equiv) in THF (10 mL) was added to a suspension of [PPh_4_][Cp*MoFe_3_S_4_Cl_3_] (1.00 g, 1.03 mmol) in THF (10 mL). The solution was stirred for 2 min and a solution of Cp_2_Co (214 mg, 1.13 mmol, 1.1 equiv) in THF (2 mL) was added dropwise. The solution was stirred for 30 min, then filtered through Celite, and concentrated to 10 mL. Et_2_O (100 mL) was added, and the mixture was stirred for 5 min. The dark precipitates were collected on a frit and washed with additional Et_2_O (3 × 10 mL). The solids were dried *in vacuo*. Yield: 999 mg (83%). Crystals suitable for XRD were grown by diffusion of *n*-pentane into a THF solution of Cp*MoFe_3_S_4_(IMes)_2_Cl at room temperature. ^1^H NMR (400 MHz, CD_2_Cl_2_, 293 K) δ 7.55 (s, 4H IMes), 7.41 (s, 4H, IMes), 5.03 (s, 4H, IMes), 3.07 (s, 12H, Mes C*H*_3_), 2.72 (s, 12H, Mes C*H*_3_), 2.27 (s, 12H, Mes C*H*_3_), −6.47 (s, 15H, Cp*).

#### Cp*MoFe_3_S_4_(IMes)_2_CO (1)

Cp*MoFe_3_S_4_(IMes)_2_Cl (250. mg, 0.213 mmol) was dissolved in THF (5 mL) and frozen in a Schlenk flask in liquid N_2_. A solution of Ti(N(^*t*^Bu)Ar)_3_ (185 mg, 0.320 mmol, 1.5 equiv) in THF (5 mL) was layered on top of this solution and frozen. The flask was capped with a septum and removed from the glovebox, maintaining the temperature at −196°C. The flask was evacuated, and CO (8 mL) was added via syringe. The flask was warmed to room temperature and the solution stirred vigorously for 5 min. The solution was then concentrated to 2 mL, and pentane (20 mL) was added. The black precipitates were collected on a frit and washed with pentane (3 × 5 mL). Yield: 223 mg (90%). Crystals suitable for XRD were grown by vapor diffusion of *n*-pentane into a solution of **1** in THF at room temperature. ^1^H NMR (400 MHz, C_6_D_6_, 293 K) δ 9.50 (s, 4H, IMes), 7.95 (s, 4H, IMes), 7.32 (s, 4H, IMes), 3.05 (s, 12H, Mes C*H*_3_), 2.88 (s, 12H, Mes C*H*_3_), 2.38 (s, 15H, Cp*), 2.02 (s, 12H, Mes C*H*_3_).

#### [Cp*MoFe_3_S_4_(IMes)_2_CO][BAr^F^_4_] ([1]^+^)

A solution of Na[BAr^F^_4_] (38 mg, 0.043 mmol) in Et_2_O (1 mL) was added dropwise to a stirred suspension of Cp*MoFe_3_S_4_(IMes)_2_Cl (50 mg, 0.043 mmol) in Et_2_O (2 mL). The solution was stirred for 10 min, then filtered through Celite, concentrated to 1 mL, and placed in a septum capped vial. To this vial, CO (6 mL) was added via syringe. The solution was stirred for 5 min, then pentane (10 mL) was added to induce crystallization of the product. The mixture was allowed to stand for 15 min, then the solvent was decanted from the crystals, and the crystals were washed with additional pentane (2 × 1 mL). The crystals were dried under a stream of N_2_ because compound [**1**]^+^ is unstable to dynamic vacuum. Yield: 59 mg (67%). Crystals suitable for XRD were grown by layering *n*-pentane onto an Et_2_O solution of [**1**]^+^ and storing the solution at −35 °C overnight. ^1^H NMR (400 MHz, *ortho*-difluorobenzene [*o*-DFB], 293 K) δ 8.30 (s, 8H, [BAr^F^_4_]^−^), 7.69 (s, 4H, IMes), 7.65 (s, 4H, [BAr^F^_4_]^−^), 5.05 (s, 4H, IMes), 3.09 (s, 15H, Cp*), 2.87 (s, 12H, Mes C*H*_3_), 2.74 (s, 12H, Mes C*H*_3_), 1.77 (s, 12H, Mes C*H*_3_). One IMes aryl resonance is obscured by the suppressed *o*-DFB resonances.

#### [K(Benzo-15-crown-5)_2_][Cp*MoFe_3_S_4_(IMes)_2_CO] ([1]^−^)

A solution of Cp*MoFe_3_S_4_(IMes)_2_CO (50.2 mg, 0.0429 mmol) and benzo-15-crown-5 (25.3 mg, 0.0943 mmol, 2.2 equiv) in THF (1 mL) was added to solid KC_8_ (9.0 mg, 0.067 mmol, 1.5 equiv). The solution was stirred for 10 min, then filtered through Celite, and concentrated to 0.2 mL. Pentane (3 mL) was added to precipitate the product. The solvent was decanted and the black solids washed with additional pentane (2 × 1 mL). Yield: 69.1 mg (93%). Crystals suitable for XRD were grown by layering *n*-pentane onto a THF solution of [**1**]^−^ and storing the solution at −35°C overnight. ^1^H NMR (400 MHz, d^8^-THF, 293 K) δ 8.72 (s, 4H, IMes), 6.83 (s, 8H, benzo-15-c-5), 6.69 (s, 4H, IMes), 6.58 (s, 4H, IMes), 3.99 (s, 8H, benzo-15-c-5), 3.77 (s, 8H, benzo-15-c-5), 3.69 (s, 8H, benzo-15-c-5), 3.64 (s, 8H, benzo-15-c-5), 2.28 (s, 12H, Mes C*H*_3_), 2.02 (s, 12H, Mes C*H*_3_), 1.74 (s, 12H, Mes C*H*_3_), 1.40 (s, 15H, Cp*).

#### Cp*MoFe_3_S_4_(IMes)_3_ (2)

A solution of Ti(N[^*t*^Bu]Ar)_3_ (73.6 mg, 0.128 mmol, 1.5 equiv) in THF (1 mL) was added to a solution of Cp*MoFe_3_S_4_(IMes)_2_Cl (97.6 mg, 0.0833 mmol) and IMes (31.0 mg, 0.102 mmol, 1.2 equiv) in THF (2 mL). The solution was stirred for 5 min, then filtered through Celite, and concentrated to 0.5 mL. Pentane (10 mL) was added to precipitate the product. The solvent was decanted and the black solids washed with additional pentane (3 × 5 mL). Yield: 85.2 mg (71%). Crystals suitable for XRD were grown by diffusion of pentane into a benzene solution of **2** at room temperature. ^1^H NMR (400 MHz, C_6_D_6_, 293 K) δ 13.14 (s, 6H, IMes backbone), 6.50 (s, 12H, IMes *m*-C*H*), 2.40 (s, 18H, IMes *p*-C*H*_3_), 1.72 (s, 36H, Mes *o*-C*H*_3_), −0.28 (s, 15H, Cp*).

#### [Cp*MoFe_3_S_4_(IMes)_3_][BAr^F^_4_] ([2]^+^)

A solution of Na[BAr^F^_4_] (38 mg, 0.043 mmol) in THF (2 mL) was added dropwise to a solution of Cp*MoFe_3_S_4_(IMes)_2_Cl (50. mg, 0.043 mmol) and IMes (17 mg, 0.056 mmol) in THF (2 mL). The solution was stirred for 5 min, then filtered through Celite, and concentrated to 0.5 mL. Pentane (10 mL) was added to precipitate the product. The solvent was decanted and the black solids washed with additional pentane (3 × 2 mL). Yield: 69 mg (70%). Crystals suitable for XRD were grown by layering hexamethyldisiloxane onto an *o*-DFB solution of [**2**]^+^ and storing the solution at −35°C overnight. ^1^H NMR (400 MHz, *o*-DFB 293 K) δ 8.32 (s, 8H, [BAr^F^_4_]^−^), 7.68 (s, 4H, [BAr^F^_4_]^−^), 7.66 (s, 12H, IMes *m*-C*H*), 6.49 (s, 6H, IMes backbone C*H*), 2.85 (s, 18H, Mes *p*-C*H*_3_), 2.72 (s, 36H, Mes *o*-C*H*_3_), 1.40 (s, 15H, Cp*).

### XAS methods

#### Fe K-edge XAS

Samples were prepared in an inert-atmosphere glovebox and were measured as solids. Samples were ground with boron nitride to a final concentration of 5 wt % Fe, pressed into 1-mm aluminum spacers, and sealed with 37-μm Kapton tape. Fe K-edge XAS data were obtained at the Stanford Synchrotron Radiation Lightsource (SSRL) at the 16-pole, 2 T wiggler beamline 9–3 under ring conditions of 3 GeV and 500 mA. Incident X-ray radiation was monochromated using a double Si(220) crystal monochromator. Samples were maintained at 10 K in a liquid He cryostat during data collection. Spectra were collected in fluorescence mode, with X-rays detected by a passivated implanted planar silicon (PIPS) detector placed at a 90° angle to the sample. Inelastic scatter was attenuated using a Soller slit with an upstream Mn filter. An Fe foil and a third ionization chamber downstream of the sample were used for internal energy calibration, setting the first inflection point of the Fe foil spectrum to 7,111.2 eV. Data were collected from 6,785 to 7,510 eV. Three scans were averaged for each sample. No evidence of photodamage was encountered.

Data were processed using the Athena software package^98^ and Igor 7. Raw data were calibrated, averaged, and the background was removed by applying a linear normalization to the pre-edge region below 7,090 eV. The post-edge region was normalized to unit intensity by applying a quadratic normalization to the post-edge region about 7,130 eV. Pre-edge peak positions were obtained from plots of the second derivatives.

#### S K-edge XAS

Samples were prepared in an inert-atmosphere glovebox and were measured as solids. Samples were prepared by grinding to a fine powder and spreading thinly onto 38-μm low-S Mylar tape. S K-edge measurements were carried out at SSRL Beamline 4–3, which is equipped with a 20-pole, 2-Tesla wiggler source. Data were measured under ring conditions of 3 GeV and 500 mA. All samples were measured in a He atmosphere at room temperature in fluorescence mode using a PIPS detector. Intensity was normalized with respect to the incident beam using a He-filled ion chamber upstream of the sample. Data were collected from 2,400 to 2,800 eV. Two to four scans were measured and averaged for each compound. No evidence of photodamage was encountered. S K-edge energies were calibrated to the lowest energy peak in the S K-edge XAS spectrum of potassium thiosulfate at 2,472.0 eV. Spectra were then averaged and the background was removed by applying a linear normalization to the pre-edge region below 2,450 eV. The edge-jump was normalized to unit intensity by applying a quadratic normalization to the post-edge region above 2,490 eV. Pre-edge peak areas were obtained via multi-peak fitting as implemented in Igor 7.

The pre-edge area of the S K-edge was used to calculate the per-bond S 3p character in the unoccupied orbitals of each cluster using an adaptation of Equation 1:^99^

(Equation 1) $$D_{o}\left(S\;1s\to \psi^{*}\right)=\alpha^{2}hl_{s}/3n$$

where $D_{o}$ is the pre-edge area, $\alpha^{2}$ reflects the S 3p character, h is the number of holes, $I_{s}$ is the radial dipole integral, and n is the number of absorbers. A value of 6.54 was used for $I_{s}$ corresponding to a μ^3^-S^2−^ ligand.^100^ A value of 4 was used for n. Rather than accounting for the number of holes “h,” the total S 3p hole character was divided by 12 to account for all of the M–S bonds.

#### HERFD Mo K-edge XAS

Samples for HERFD Mo K-edge XAS were prepared in an inert-atmosphere glovebox and were measured as solids. Samples were ground with boron nitride to a final concentration of 5 wt % Mo, packed into 1- mm diameter quartz capillaries, plugged with vacuum grease, and then sealed with epoxy. Data were measured at the Inner Shell Spectroscopy (ISS) Beamline 8-ID at the National Synchrotron Lightsource-II at Brookhaven National Laboratory. Data were measured under ring conditions of 3 GeV and 500 mA. Incident X-ray energy was selected using a Si(111) monochromator. The [999] reflection of a single Ge(111) mounted in the crystal analyzer spectrometer installed at 8-ID was used to select the Mo Kα emission line. Emitted X-rays were measured using a Pilatus 100k detector. Samples were measured at room temperature. Data were measured from 19,900 to 20,240 eV. Data were obtained by rastering over 10 unique spots on each sample, with 3 scans measured at each spot. No evidence of photodamage was encountered, so all scans were averaged. An Mo foil placed between ionization chambers downstream of the sample was used for internal energy calibration. The white line of the Mo foil spectrum was assigned to 20,016.4 eV.

Data were processed using the Athena software package^98^ and Igor 7. Raw data were calibrated, averaged, and the background was removed by applying a linear normalization to the pre-edge region below 19,975 eV. The post-edge region was normalized to unit intensity by applying a spline to the post-edge region above 20,075 eV. Rising edge energies were determined by finding maxima in first derivative plots of each spectrum. Pre-edge peak energies were determined by finding minima in second derivative plots of each spectrum.

## RESOURCE AVAILABILITY

### Lead contact

Requests for further information and resources should be directed to the lead contact, Daniel L.M. Suess (suess@mit.edu).

### Materials availability

All compounds generated in this study are available from the lead contact with a completed materials transfer agreement.

### Data and code availability

All data reported in this study are available from the lead contact. Crystallographic information files can be obtained from the Cambridge Structural Database (Cambridge Crystallographic Database [CCDC]): [**1**]^+^ (CSD: 2520144), [**1**] (CSD: 2520140), [**1**]^−^ (CSD: 2520414), [**2**]^+^ (CSD: 2520143), [**2**] (CSD: 2520142), and Cp*MoFe_3_S_4_(IMes)_2_Cl (CSD: 2520139).

## Supplementary Material

MMC1

SUPPLEMENTAL INFORMATION

Supplemental information can be found online at https://doi.org/10.1016/j.chempr.2026.103134.

## Figures and Tables

**Table 1.** Tabulated features of the XAS spectra

	Mo K-edge HERFD		Fe K-edge		S K-edge	
Compound	Pre-edge energy (eV)	Edge energy (eV)	Pre-edge energy (eV)	Edge energy (eV)	Pre-edge energy (eV)	Area

**[1]^+^**	20,002.3	20,009.0	7,111.5 7,114.3	7,118.2	2,472.3	3.6(1)
**1**	20,002.3	20,008.0	7,111.7 7,113.9	7,117.8	2,472.6	3.79(4)
**[1]^−^**	20,002.2	20,008.0	7,111.6 7,113.5	7,117.2	2,472.3	3.59(1)
**[2]^+^**	20,002.0	20,008.0	7,111.6	7,117.9	2,472.3	3.56(4)
**2**	20,002.0	20,008.0	7,111.5	7,117.8	2,472.6	3.5(1)
**3**	–	–	7,111.5	7,116.9	2,472.0	2.10(1)
**[4]^+^**	–	–	7,111.4	7,116.8	2,472.1	1.62(1)
**4**	–	–	7,111.4 7,113.2	7,116.8	2,472.2	1.00(1)

## Highlights

- In-depth characterization of [MoFe_3_S_4_] and [Fe_4_S_4_] clusters in multiple charge states
- The Fe centers in both series are adept at backbonding to the strong π-acid CO
- Backbonding to weak π-acids is strong only for the [MoFe_3_S_4_] clusters
- Mo ions electronically facilitate backbonding at neighboring Fe ions
