# Supplementary material for: Molybdenum doping enhances backbonding at neighboring iron centers in iron-sulfur clusters
Source: Chem. Author manuscript; Available in PMC 2026 Sep 29. (PMC13619170; doi:10.1016/j.chempr.2026.103134)
Supplement: Supplementary file 1 [file NIHMS2199901-supplement-MMC1.pdf]

Chem, Volume *13*

Supplemental information

Molybdenum doping enhances backbonding at neighboring iron centers in iron-sulfur clusters

Alexandra C. Brown, Samantha N. MacMillan, Alex McSkimming, Denis Leshchev, Eli Stavitski, Kyle M. Lancaster, and Daniel L.M. Suess

A. Supplemental methods

Additional General Considerations

Glassware was dried in an oven at 160 °C prior to use. Molecular sieves (3 Å), and Celite® were activated by heating to 300 °C overnight under vacuum prior to storage under an atmosphere of N_2. *O*-difluorobenzene (DFB) was distilled from CaH_2, C_6D_6 was degassed by three freeze–pump–thaw cycles, and other solvents were degassed by sparging with argon and dried by passing through a column of activated alumina. All solvents were stored under an N_2 atmosphere over 3 Å molecular sieves.

NMR spectra were recorded on Bruker 400 and 500 MHz spectrometers. ^1H chemical shifts are given relative to residual solvent peaks; spectra in DFB and Et_2O are referenced to the triplet of residual *n*-pentane at 0.89 ppm. Solvent suppression for NMR in protonated solvents was carried out using WET solvent suppression.[1] FT-IR samples were taken as thin films using a Bruker Alpha Platinum ATR spectrometer with OPUS software in a glovebox under an N_2 atmosphere. EPR spectra were recorded on a Bruker EMX spectrometer at 9.37 GHz (perpendicular mode) as frozen glasses. Simulations were performed using EasySpin[2] (5.2.21) in Matlab (R2017b). UV-vis spectra were recorded on a Cary 50 spectrometer. Zero-field ^{57}Fe Mössbauer spectra were measured with a constant-acceleration spectrometer at 80 K using a SEE co. W302 constant-acceleration spectrometer. Isomer shifts are quoted relative to α-Fe foil at room temperature; Mössbauer spectra were simulated with WMOSS v.4.[3] Elemental analyses were performed at Midwest Microlab. X-ray structural determinations were performed at the MIT diffraction facility using a Bruker X8 diffractometer with an APEX II CCD detector or a Bruker D8 Venture diffractometer with a Photon2 CPAD detector. Diffraction data was collected, integrated, and corrected for absorption using Bruker APEX3 software and its associated modules (SAINT, SADABS, TWINABS).

Structural solutions and refinements (on F^2) were carried out using SHELXT and SHELXL-2018 in ShelXle.[4] Ellipsoid plots and figures were made using Mercury 2021.2.0.

Statement on Compound Purity

The purity of all compounds was assessed by a variety of spectroscopic and analytical methods as detailed below. All compounds are air-sensitive and [**1**]$^+$ is unstable towards dynamic vacuum, but all compounds can be isolated as crystalline solids in high purity as determined by NMR, EPR, and Mössbauer spectroscopic analysis, SQUID magnetometry, and H and N content from elemental analysis. Low C content was obtained by elemental analysis as has been observed for other members of this class of molecules[5–7] and in other contexts.[8] Elemental analysis results are as follows:

Cp*$MoFe_3S_4(IMes)_2Cl$ (**1-Cl**): Anal. Found: C, 51.54; H, 5.25; N, 4.40. Calcd. for $C_{52}H_{63}ClN_4Fe_3S_4Mo$: C, 53.32; H, 5.42; N, 4.78

Cp*$MoFe_3S_4(IMes)_2CO$ (**1**): Anal. Found: C, 49.85; H, 5.22; N, 4.35. Calcd. for $C_{53}H_{63}N_4Fe_3S_4MoO$: C, 54.70; H, 5.46; N, 4.81

[Cp*$MoFe_3S_4(IMes)_2CO$][BAr^F_4] ([**1**]$^+$): Anal. Found: C, 49.72; H, 3.73; N, 2.79. Calcd. for $C_{85}H_{75}BF_{24}N_4Fe_3S_4MoO$: C, 50.73; H, 3.73; N, 2.76

[K(Benzo-15-crown-5)$_2$][Cp*$MoFe_3S_4(IMes)_2CO$] ([**1**]$^-$): Anal. Found: C, 50.78; H, 5.55; N, 2.89. Calcd. for $C_{81}H_{103}N_4Fe_3S_4MoO_{11}K$: C, 55.93; H, 5.97; N, 3.22

Cp*$MoFe_3S_4(IMes)_3$ (**2**): Anal. Found: C, 57.82; H, 6.43; N, 5.50. Calcd. for $C_{73}H_{87}N_6Fe_3S_4Mo\cdot C_5H_{12}$: C, 61.94; H, 6.60; N, 5.56

[Cp*$MoFe_3S_4(IMes)_3$][BAr^F_{24}] ([**2**]$^+$): Anal. Found: C, 54.37; H, 4.30; N, 3.67. Calcd. for $C_{105}H_{99}BF_{24}N_6Fe_3S_4Mo$: C, 54.75; H, 4.33; N, 3.65

B. NMR Spectra

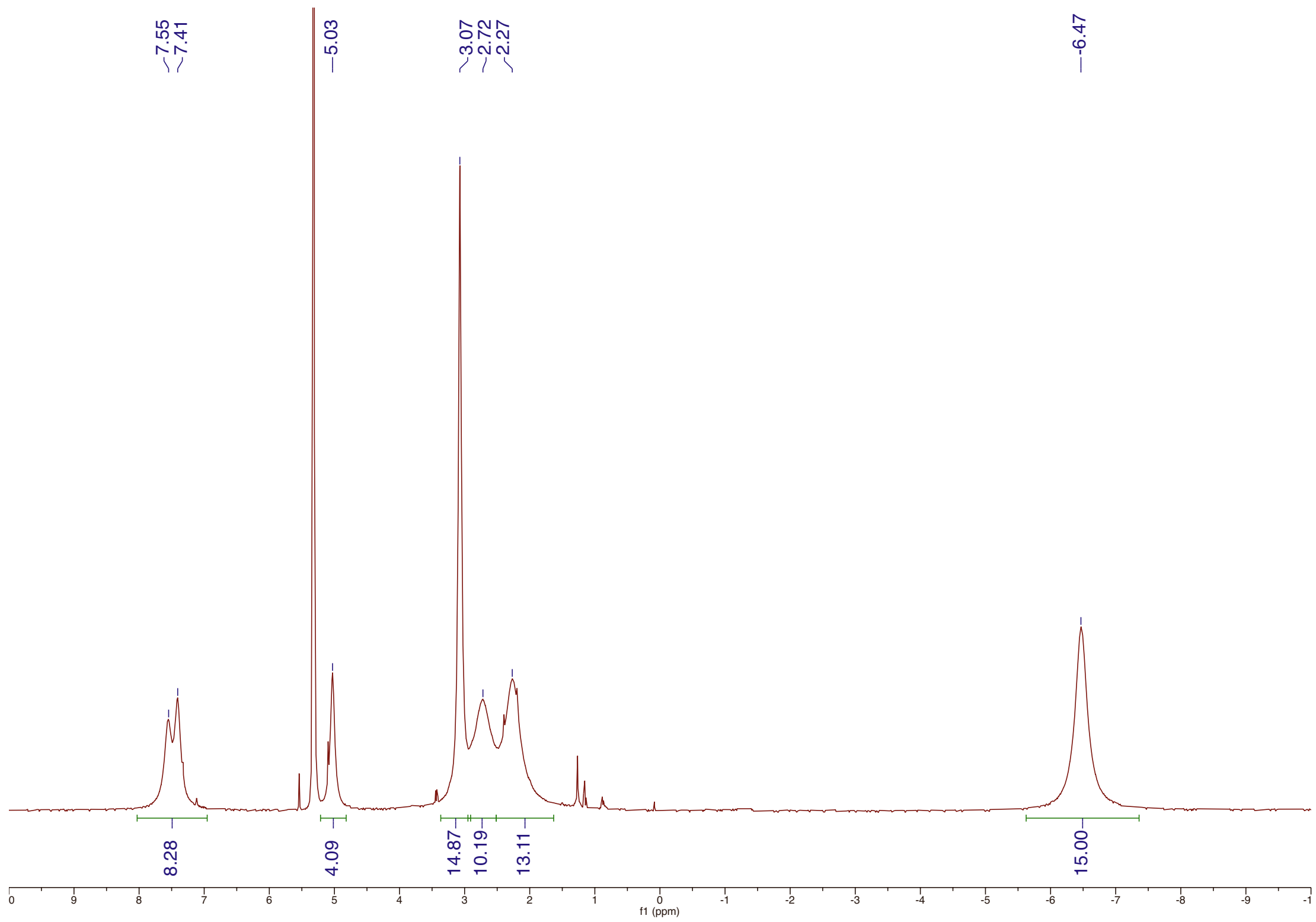

Figure S1: ^{1}H NMR spectrum of **1-Cl** in CD_2Cl_2 at 293 K

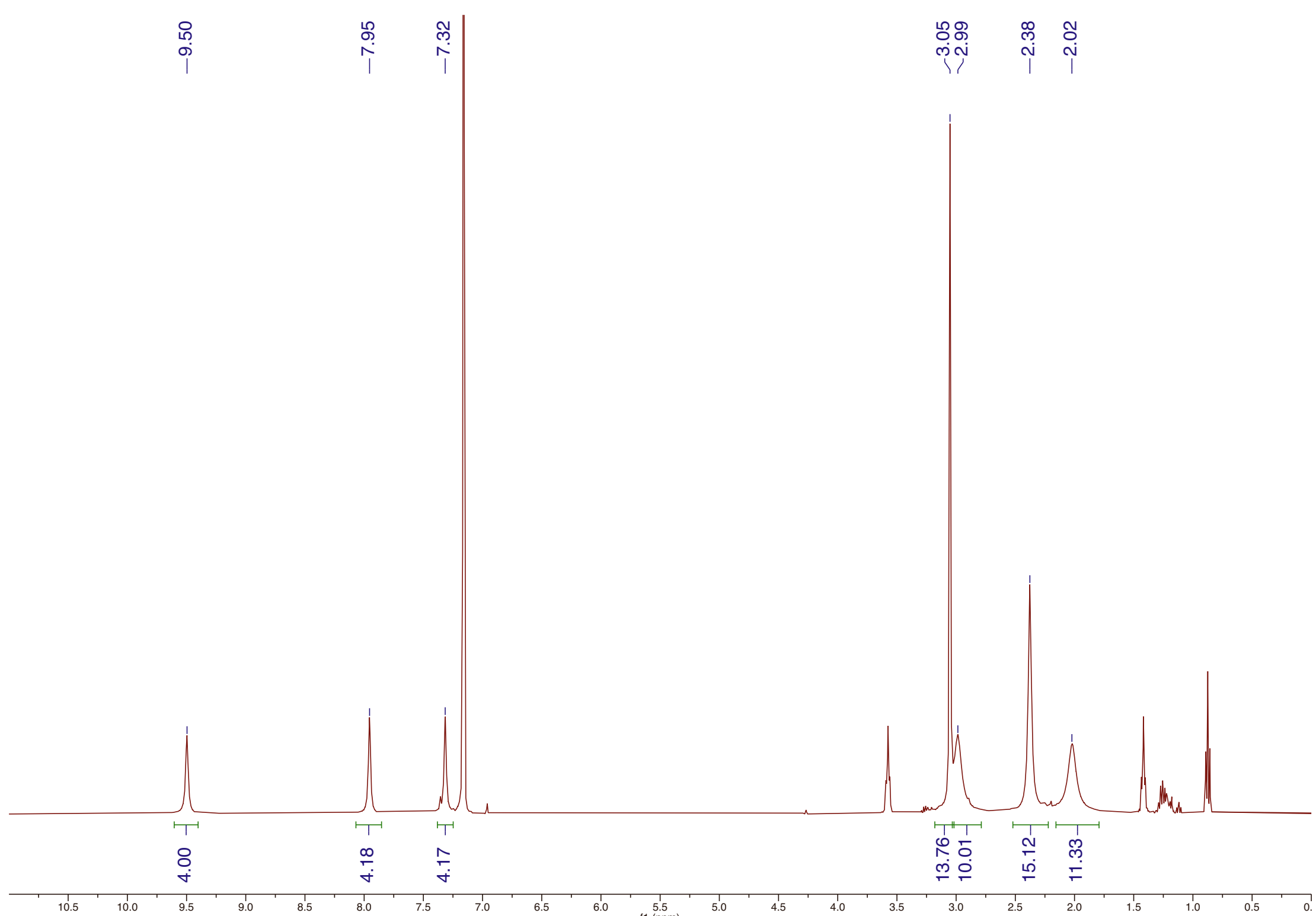

Figure S2: ^{1}H NMR spectrum of **1** in C_6D_6 at 293 K

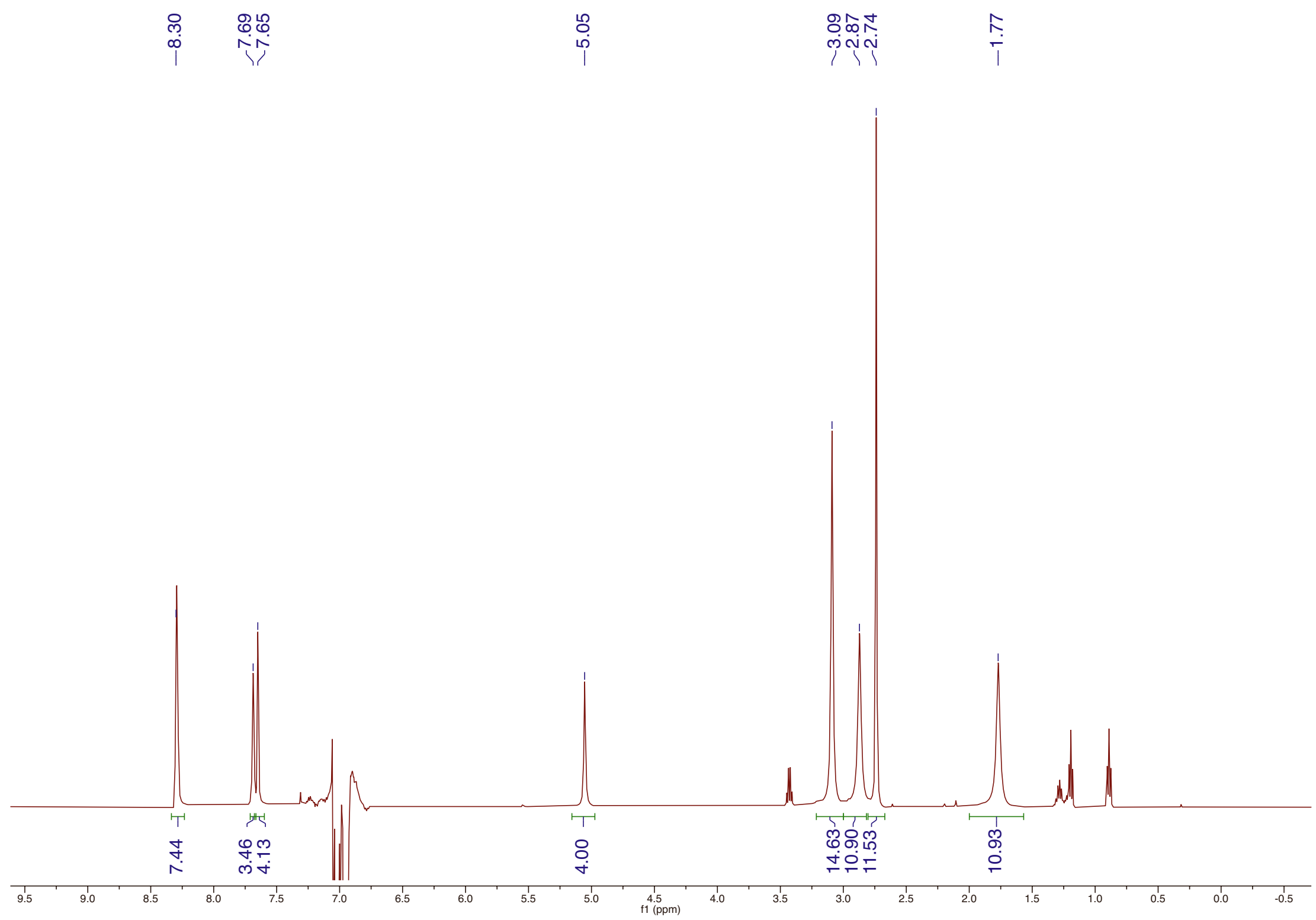

Figure S3: ^{1}H NMR spectrum of [**1**][BAr^{F}_{4}] in DFB at 293 K

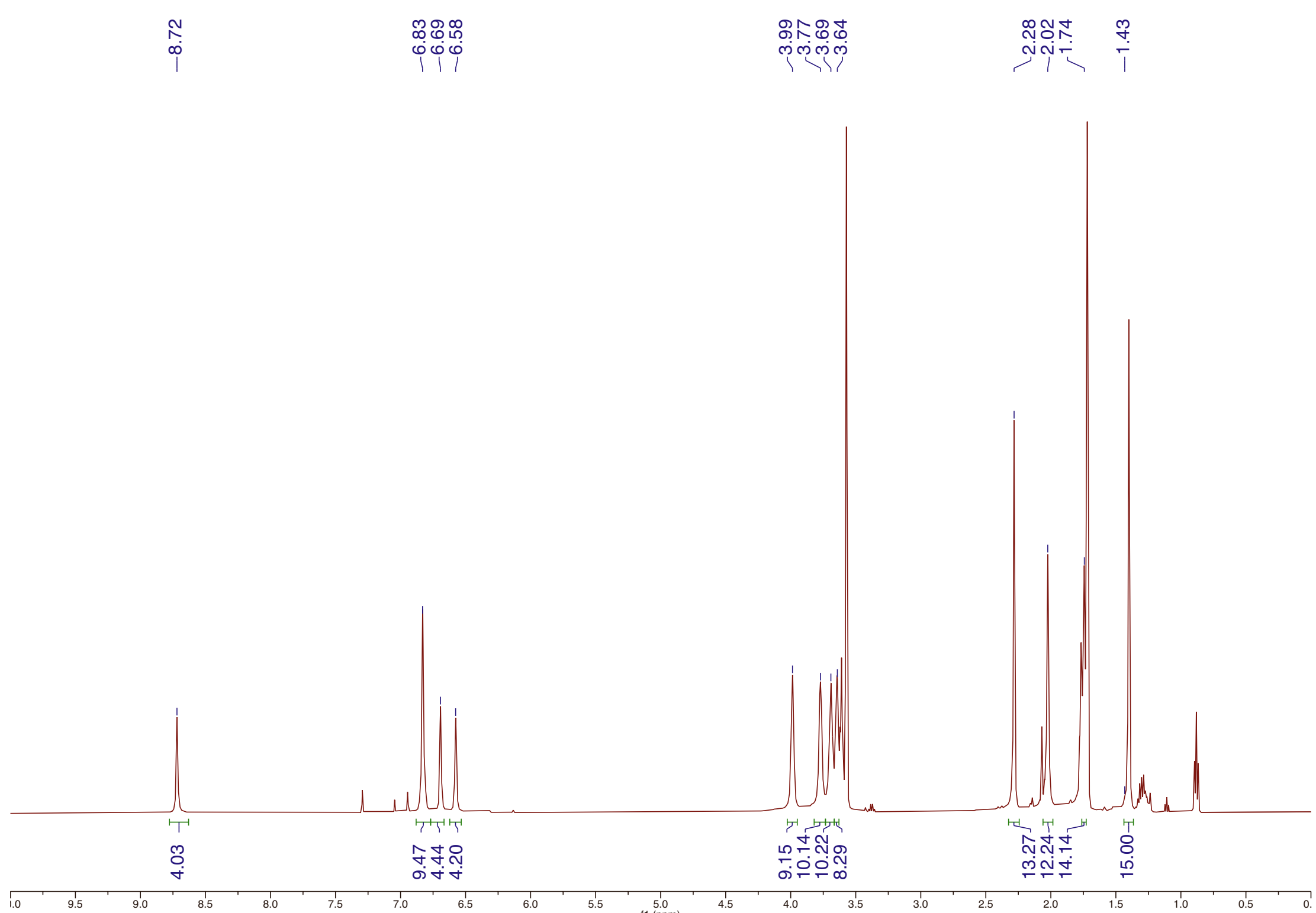

Figure S4: ^{1}H NMR spectrum of [K(Benzo-15-crown-5)$_2$][**1**] in d^8-THF at 293 K

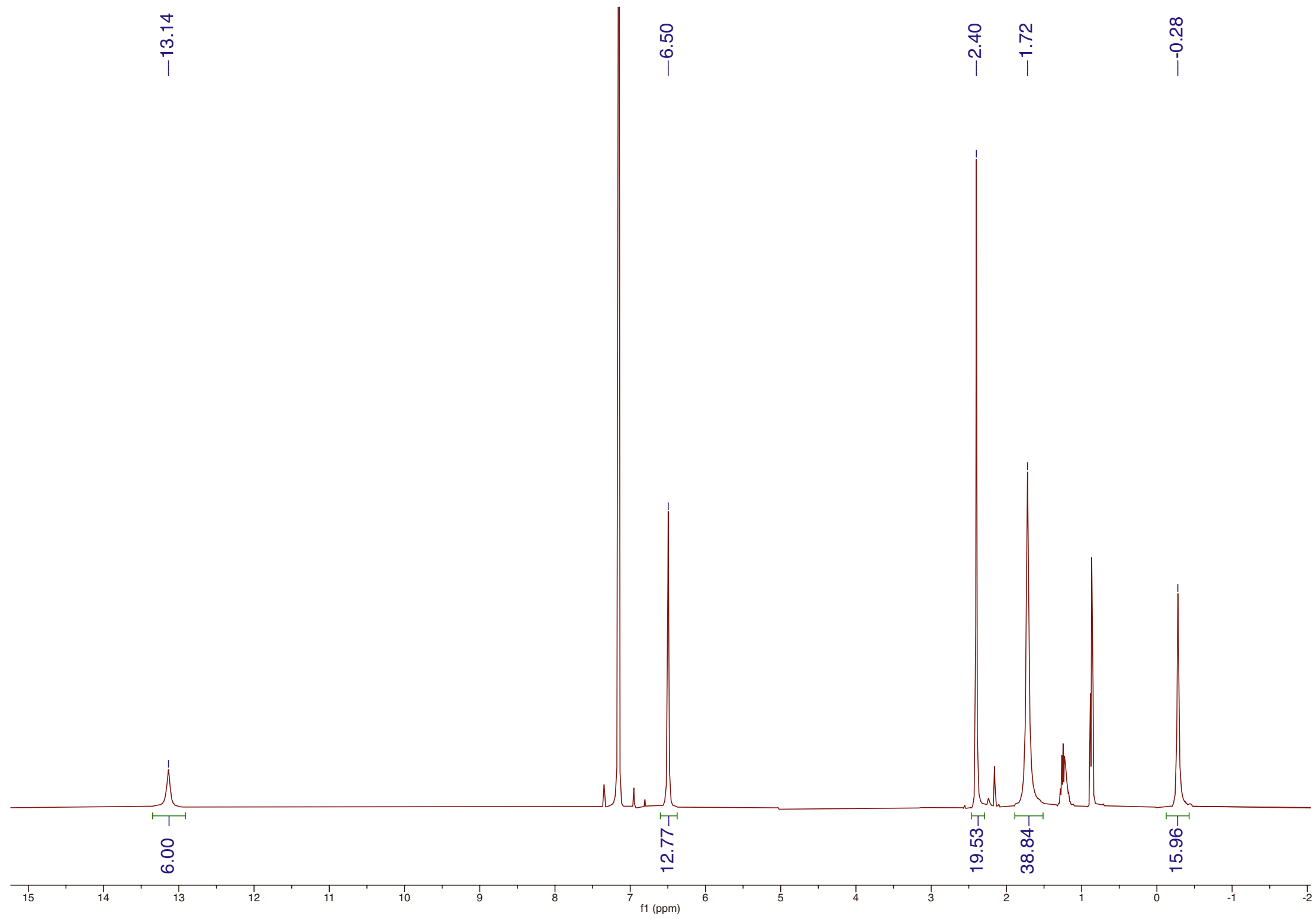

Figure S5: ^{1}H NMR spectrum of **2** in C_6D_6 at 293 K

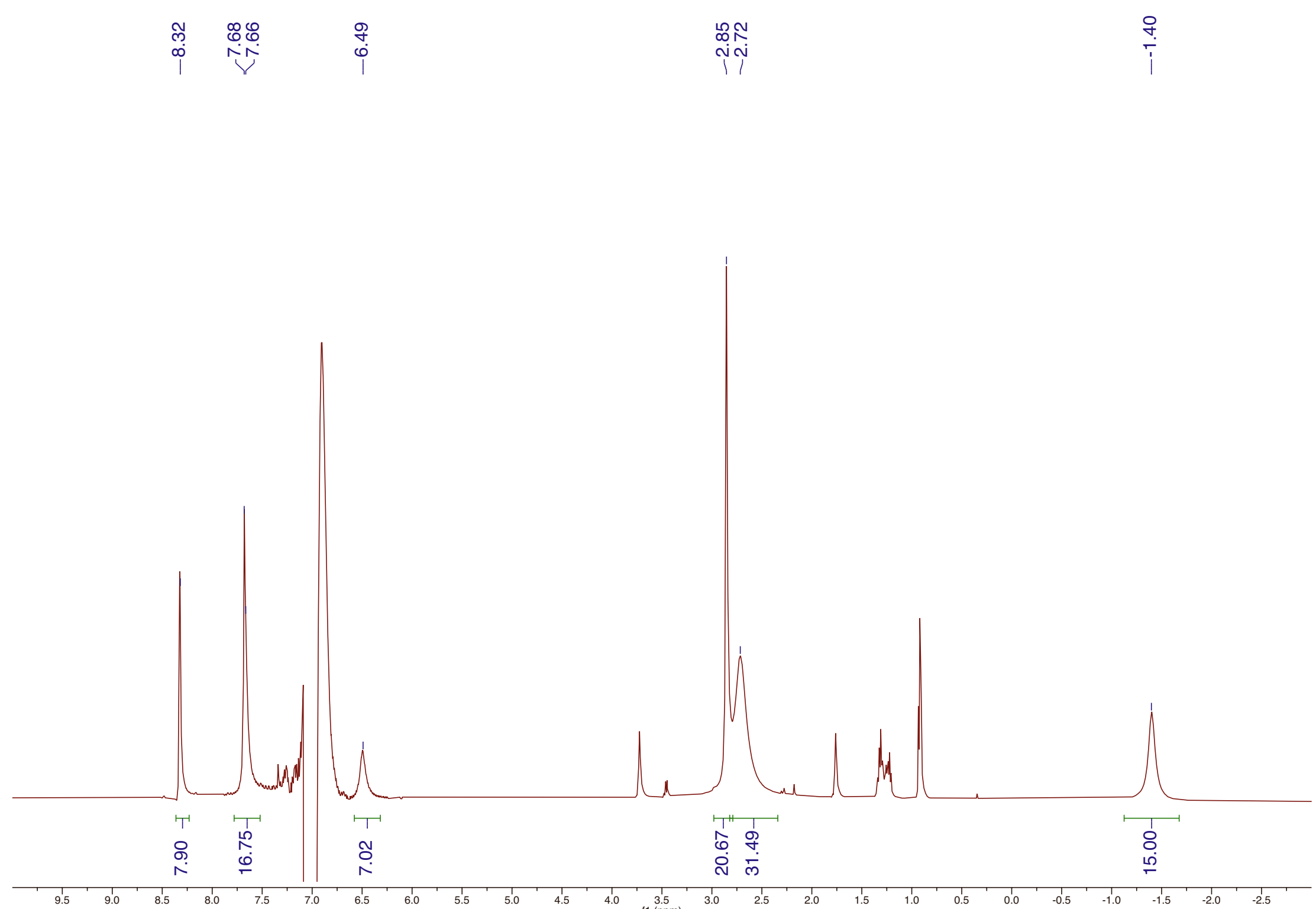

Figure S6: ^{1}H NMR spectrum of [**2**][BAr$^{F}_{24}$] in DFB at 293 K

C. IR Spectra

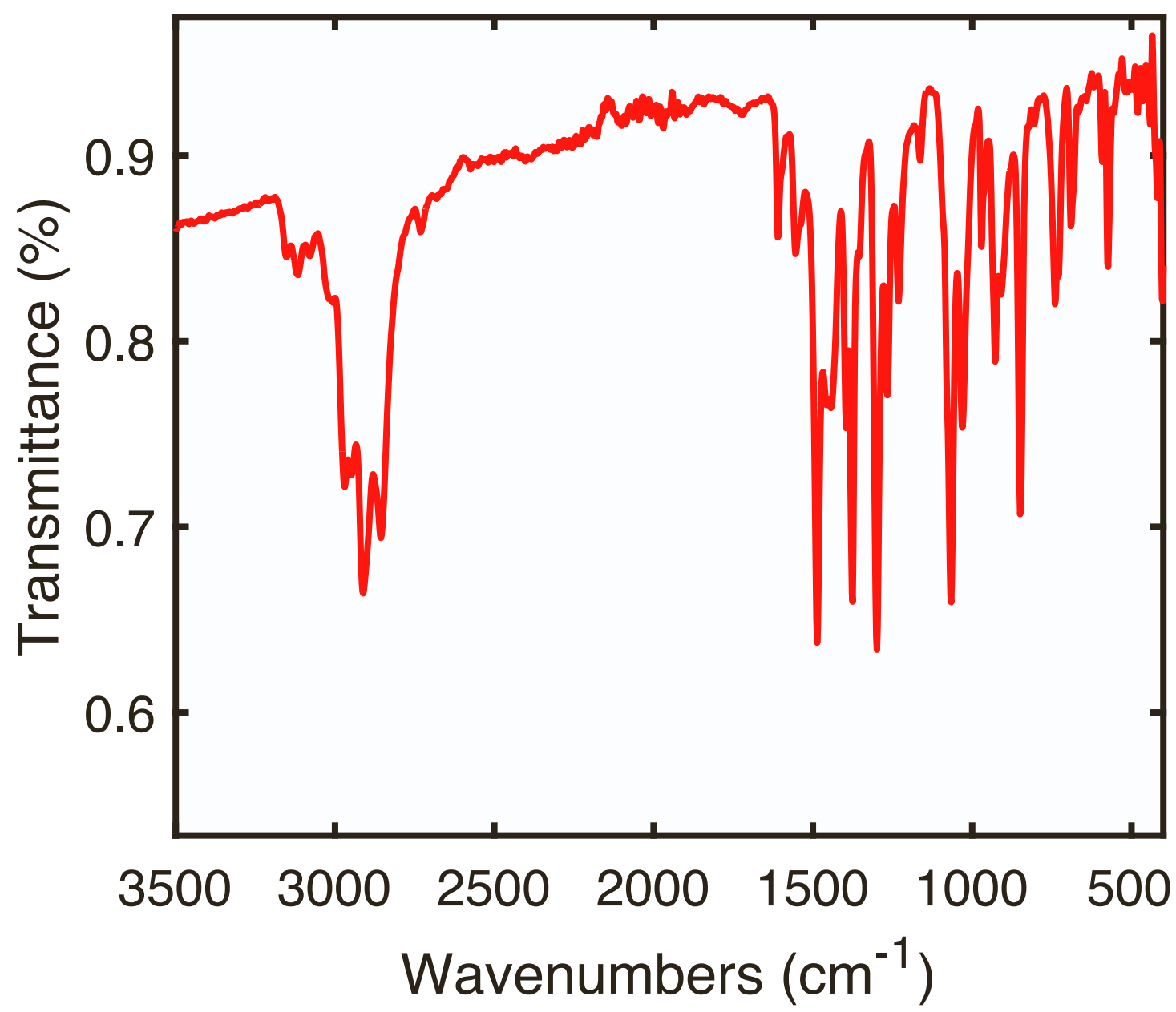

Figure S7: IR spectrum of **1-Cl**.

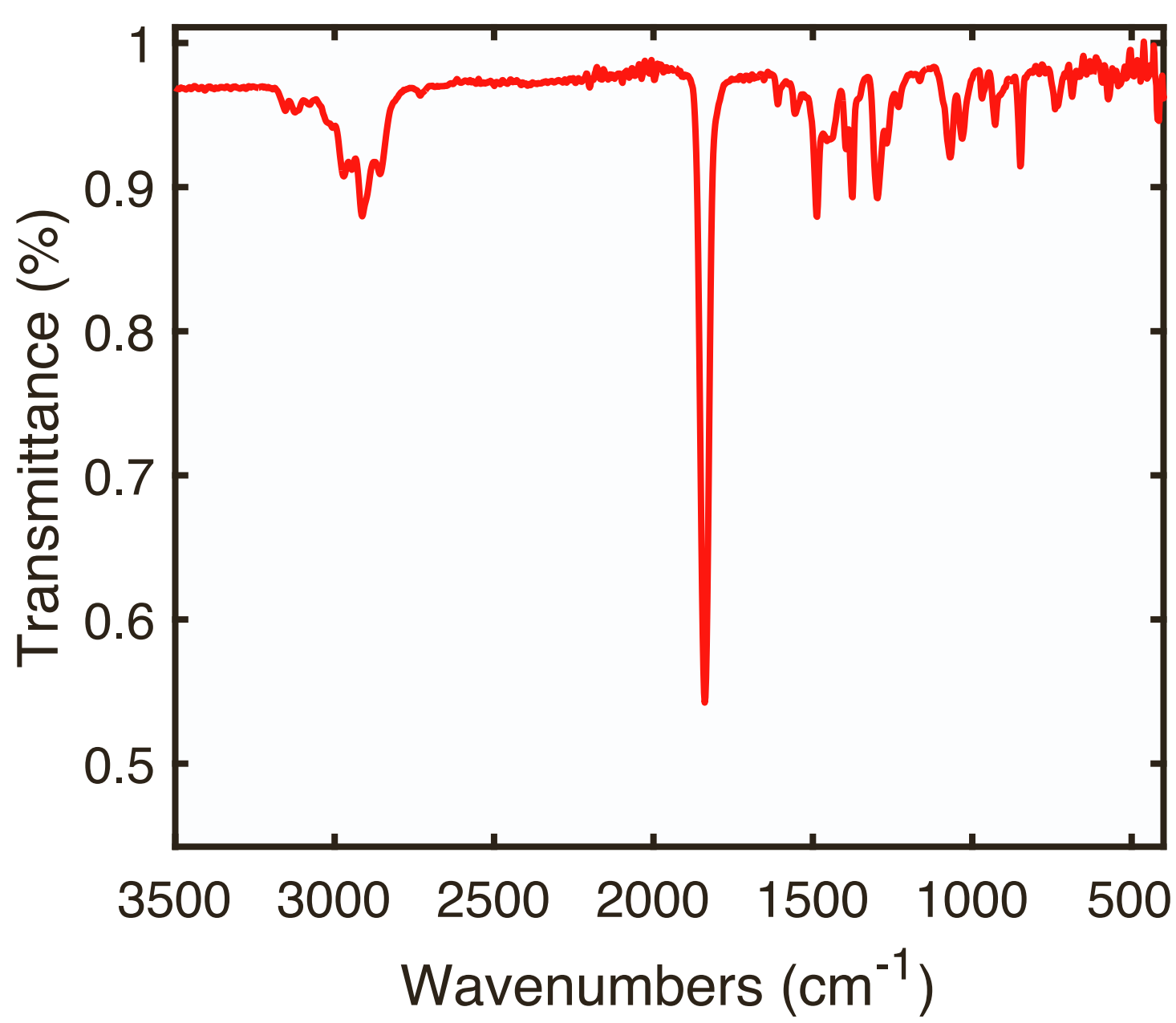

Figure S8: IR spectrum of **1**. ν(C–O) = 1839 cm^{-1}

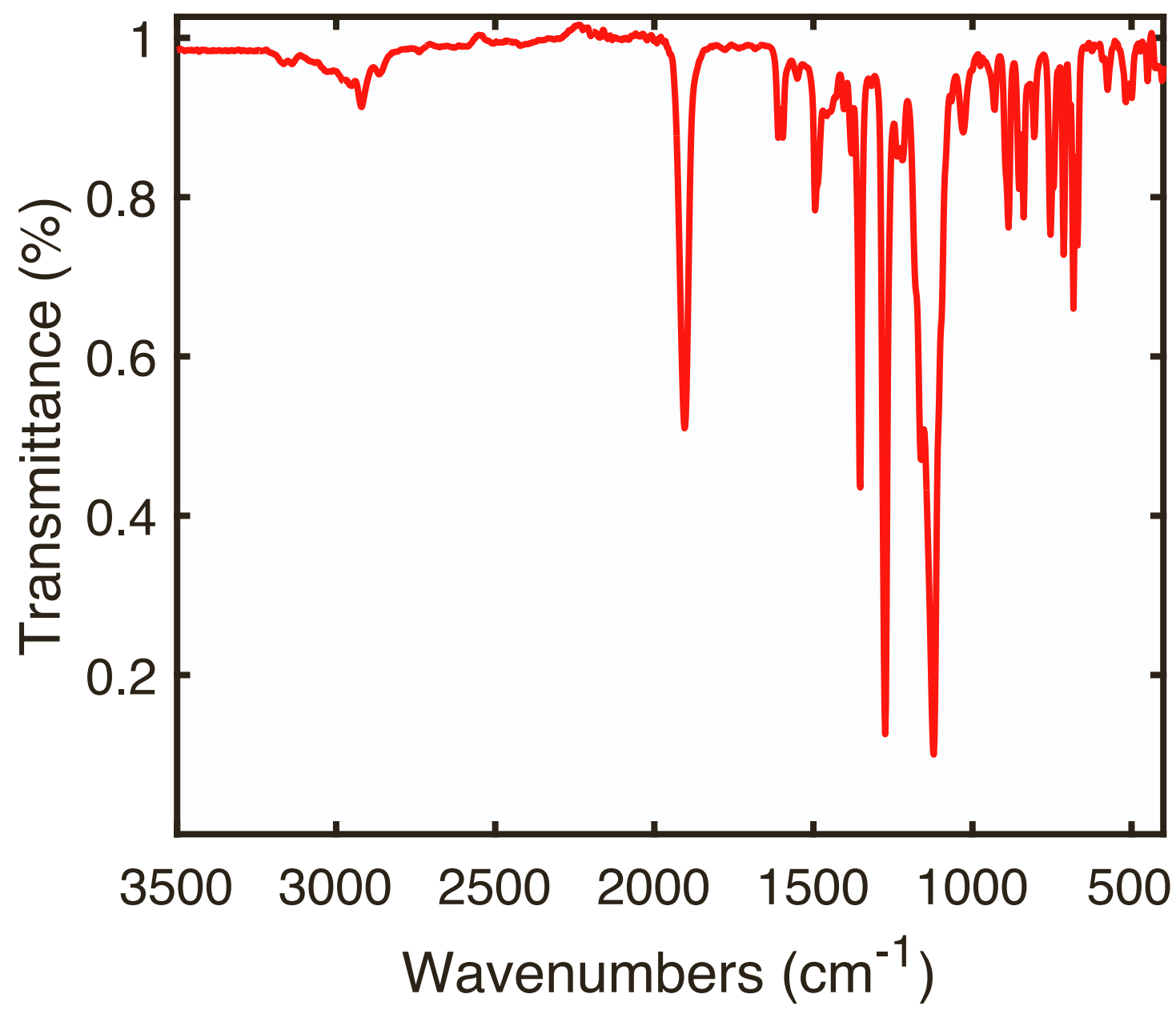

Figure S9: IR spectrum of [**1**][$BAr^F{}_4$]. ν(C–O) = 1905 cm^{-1}

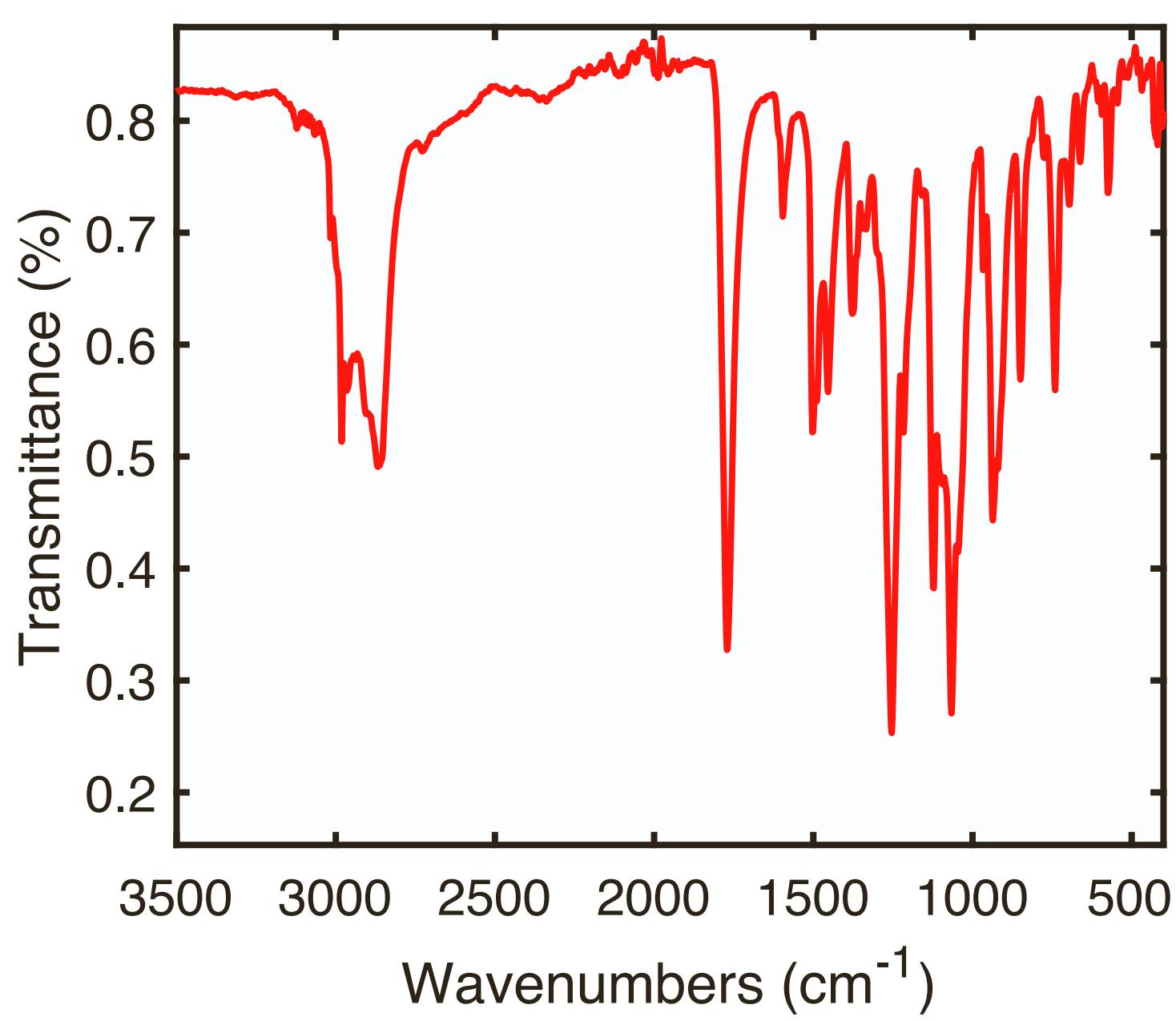

Figure S10: IR spectrum of [K(Benzo-15-crown-5)$_2$][**1**]. ν(C–O) = 1773 cm^{-1}

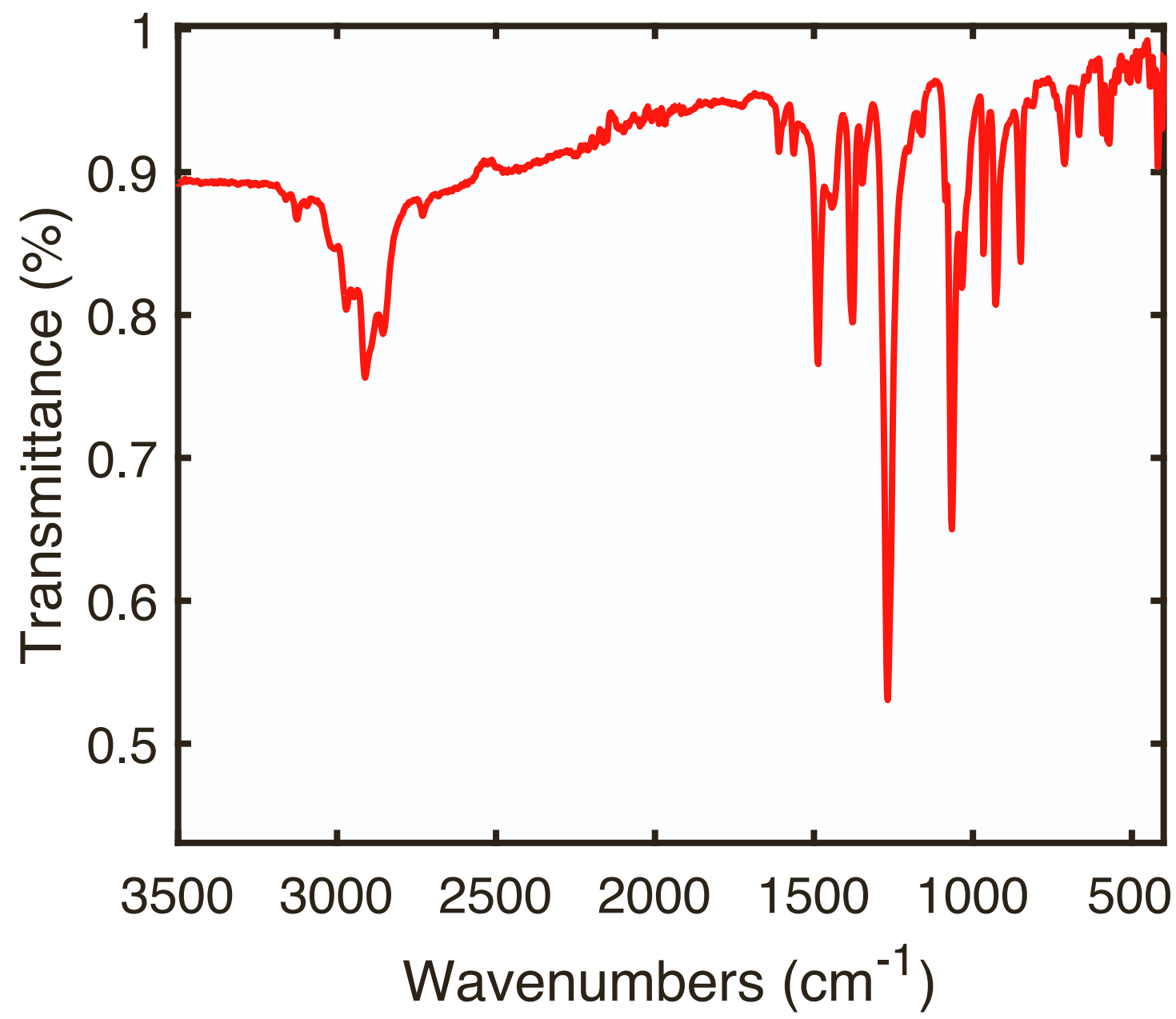

Figure S11: IR spectrum of **2**.

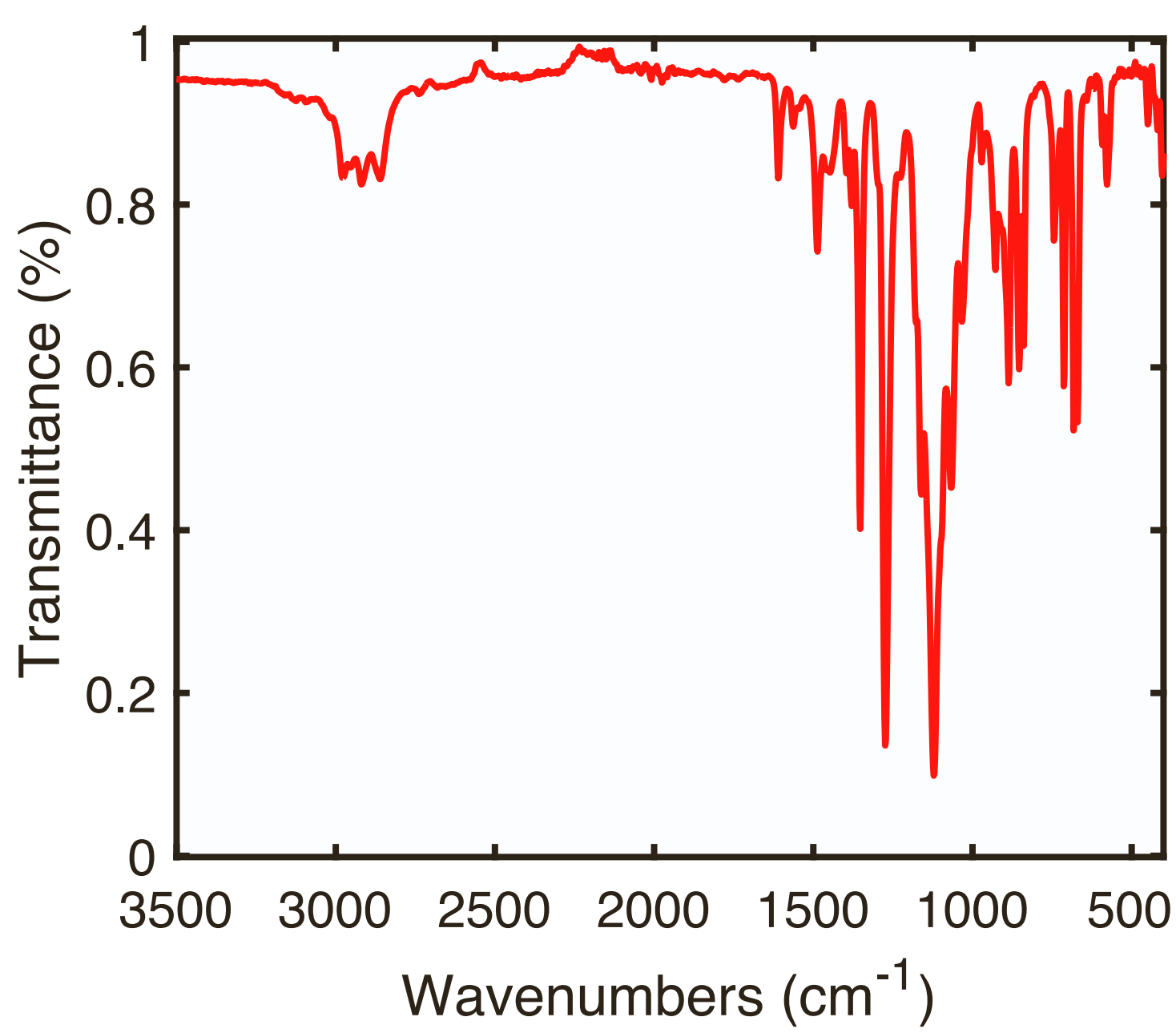

Figure S12: IR spectrum of [**2**][$BAr^F{}_4$].

D. EPR Spectra

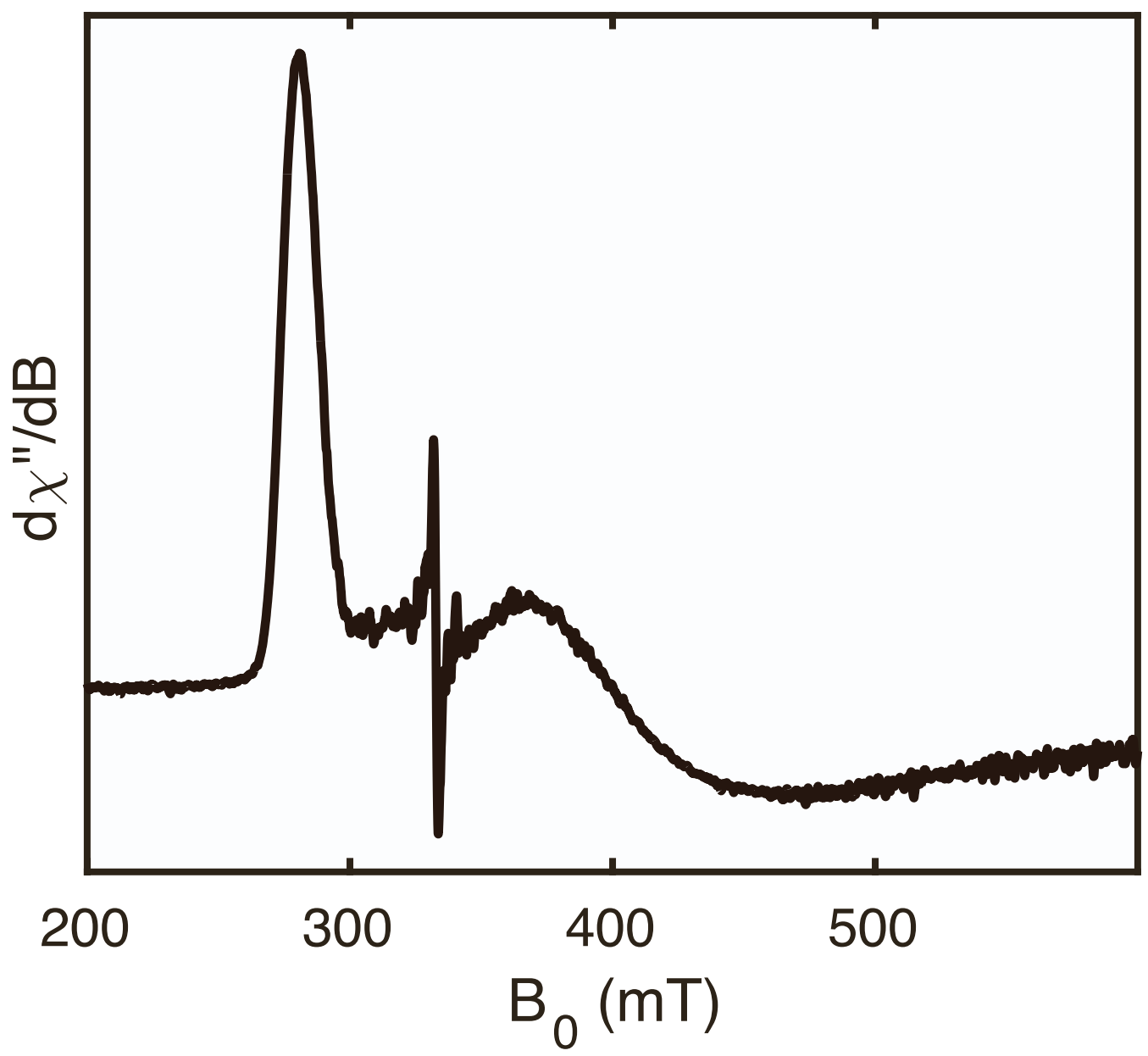

Figure S13: EPR spectrum of **2** in toluene at 5 K (perpendicular mode, black). Microwave power: 250 μW; microwave frequency: 9.3732 GHz. $g_1 \sim 2.39$, $g_2 \sim 1.65$.

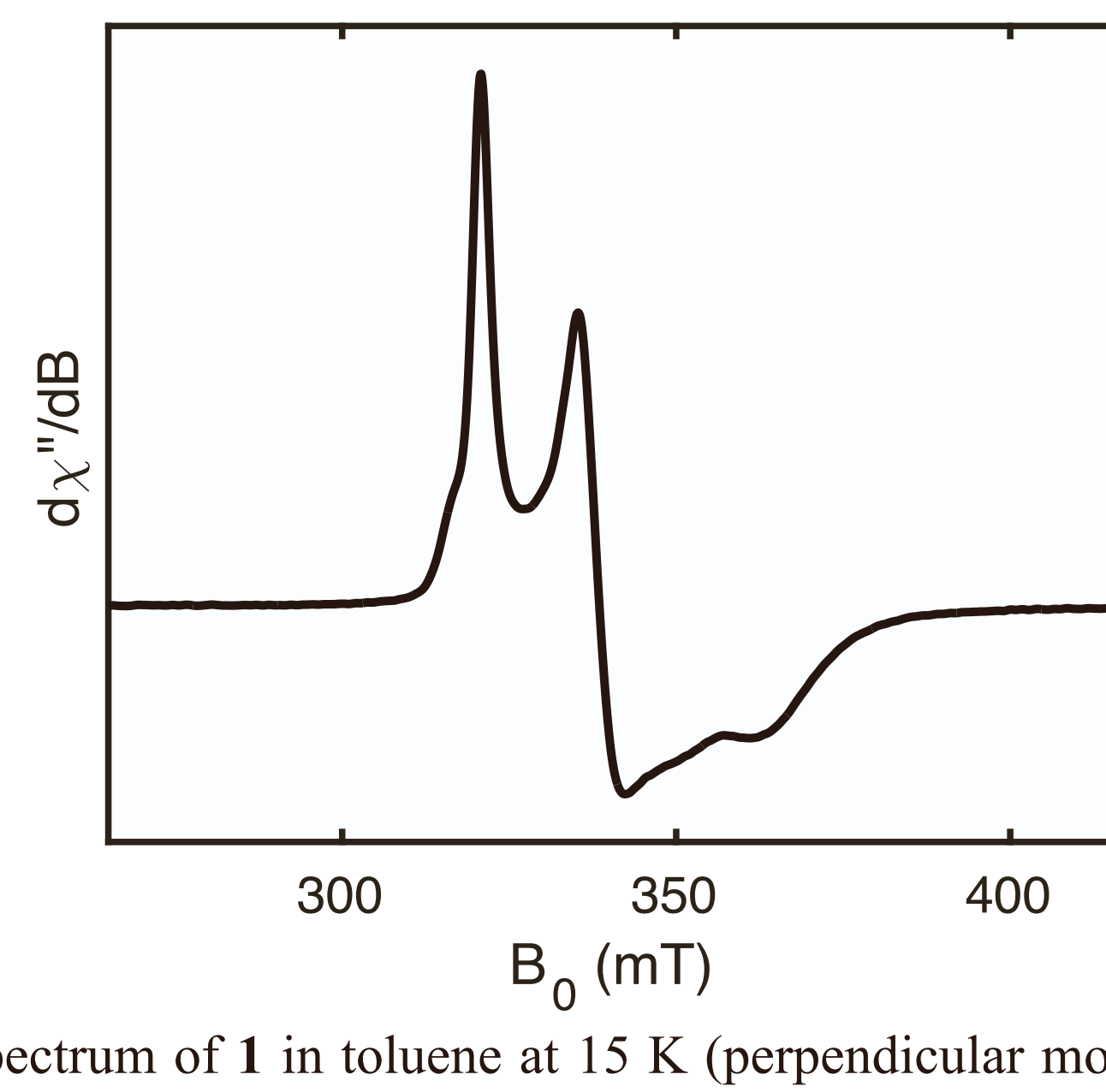

Figure S14: EPR spectrum of **1** in toluene at 15 K (perpendicular mode, black). Microwave power: 63 μW; microwave frequency: 9.3678 GHz. $g_1 = 2.08$, $g_2 = 1.98$, $g_3 = 1.85$.

E. UV-vis spectra

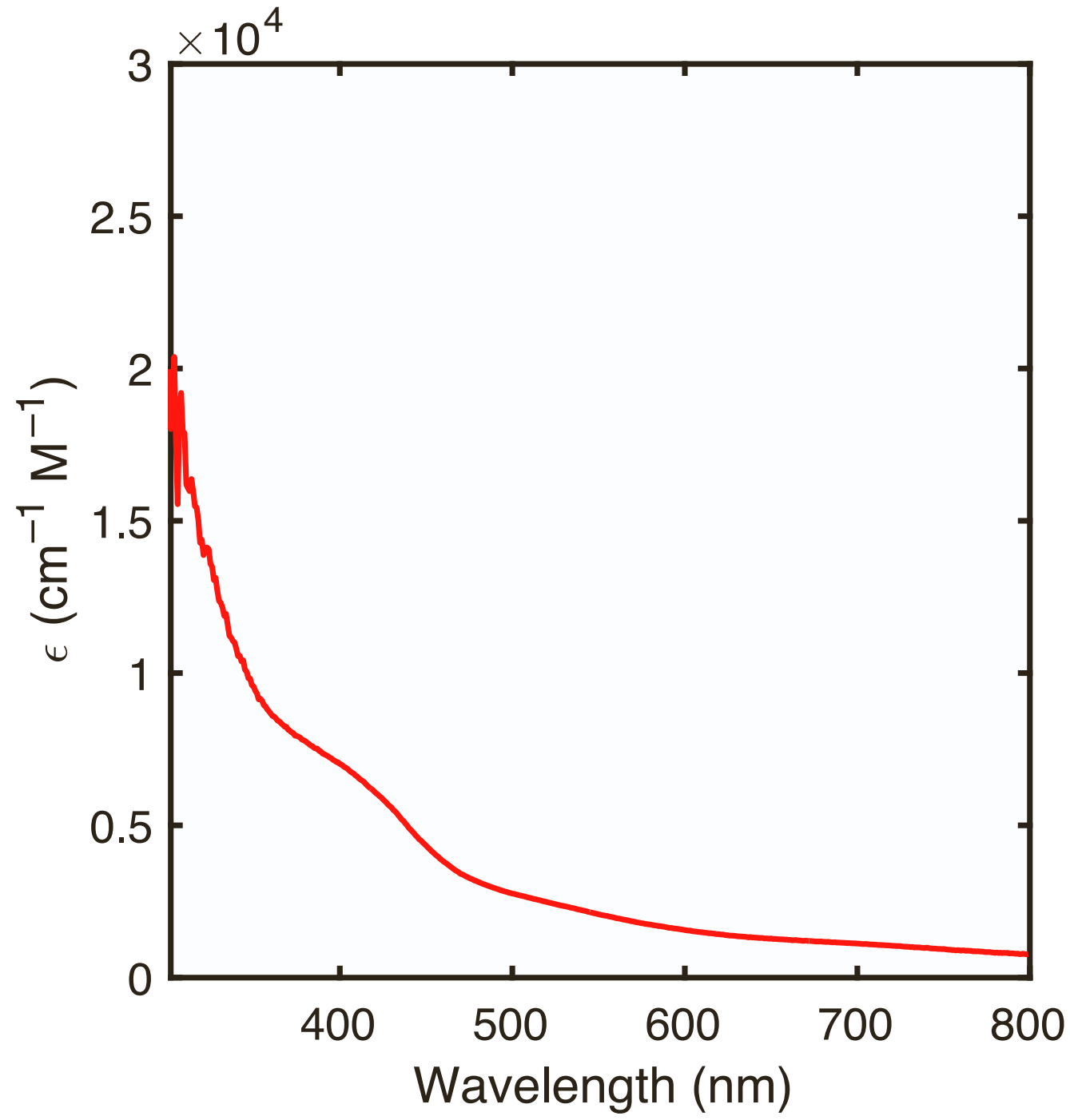

Figure S15: UV-vis spectrum of **1-Cl** in THF

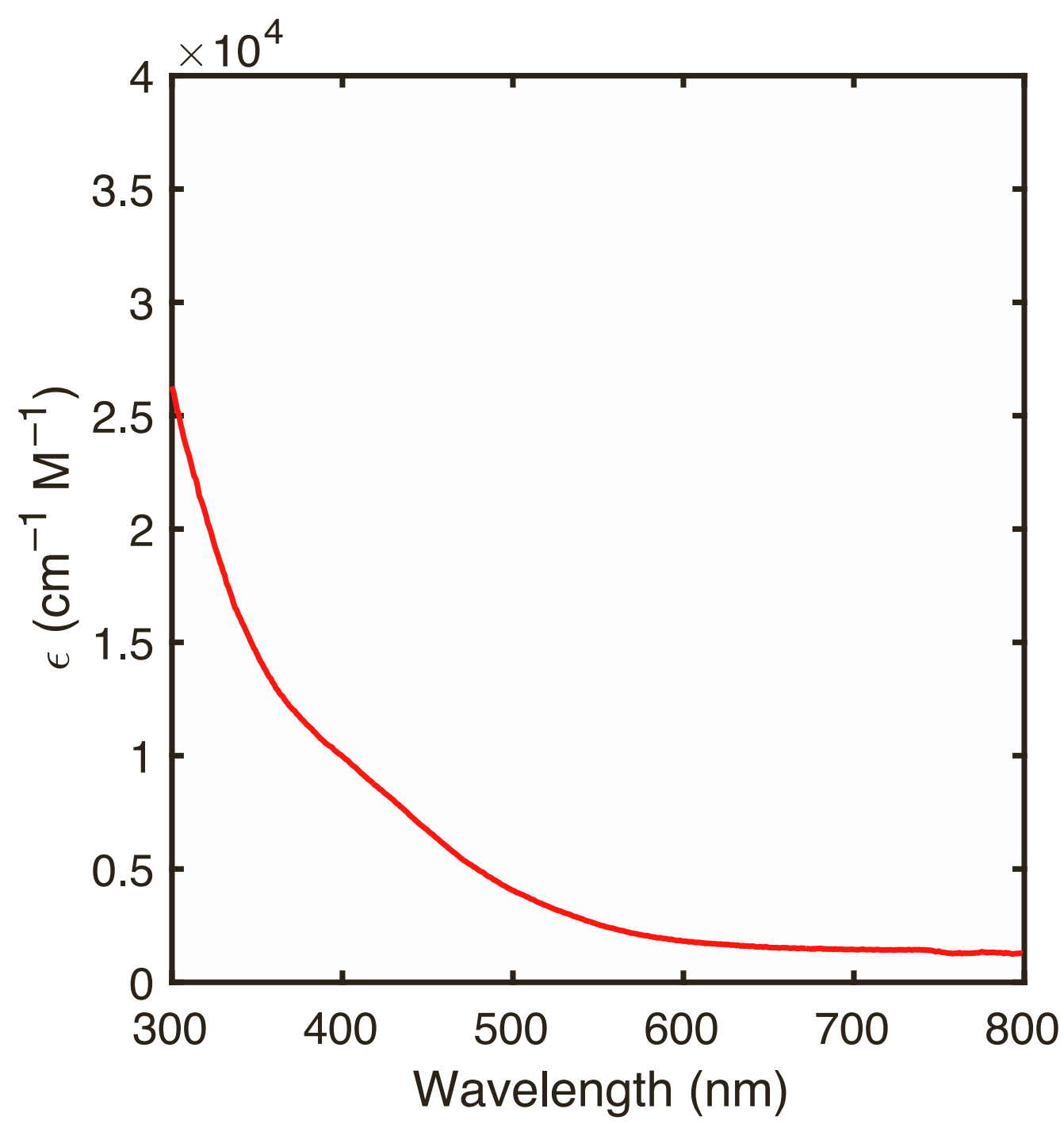

Figure S16: UV-vis spectrum of **1** in THF

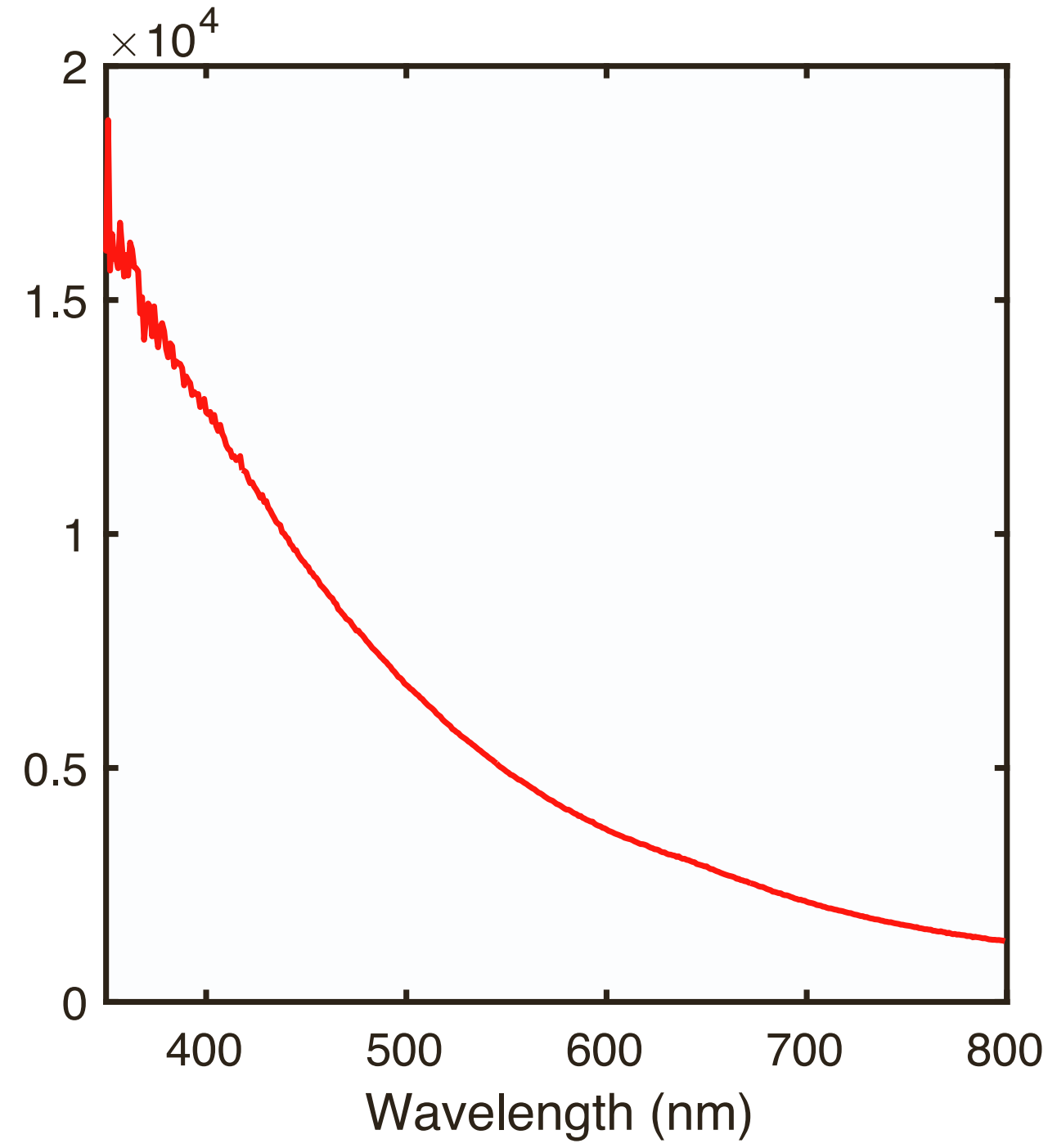

Figure S17: UV-vis spectrum of [**1**]$^{+}$ in DFB

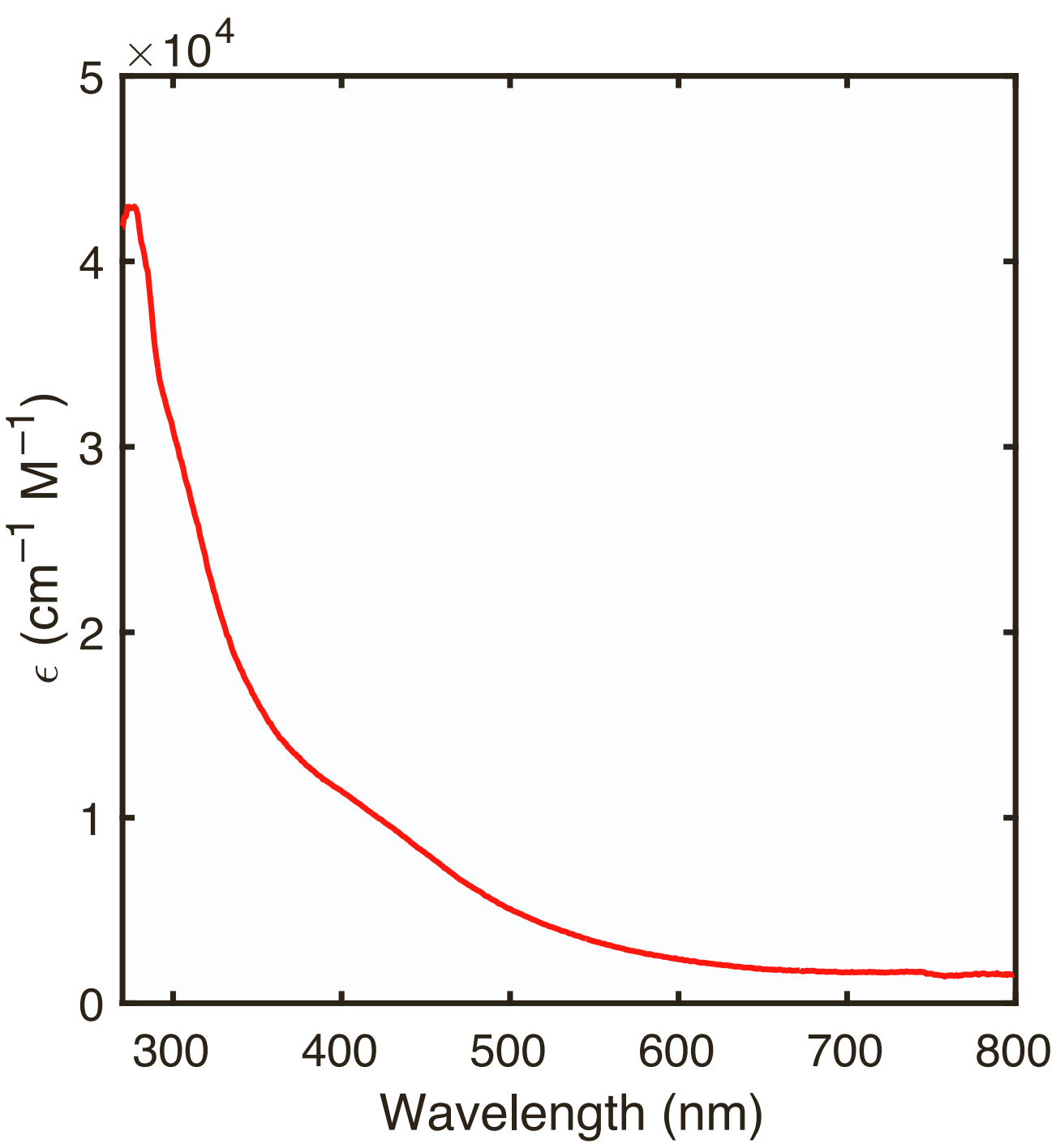

Figure S18: UV-vis spectrum of [**1**]$^{-}$ in THF

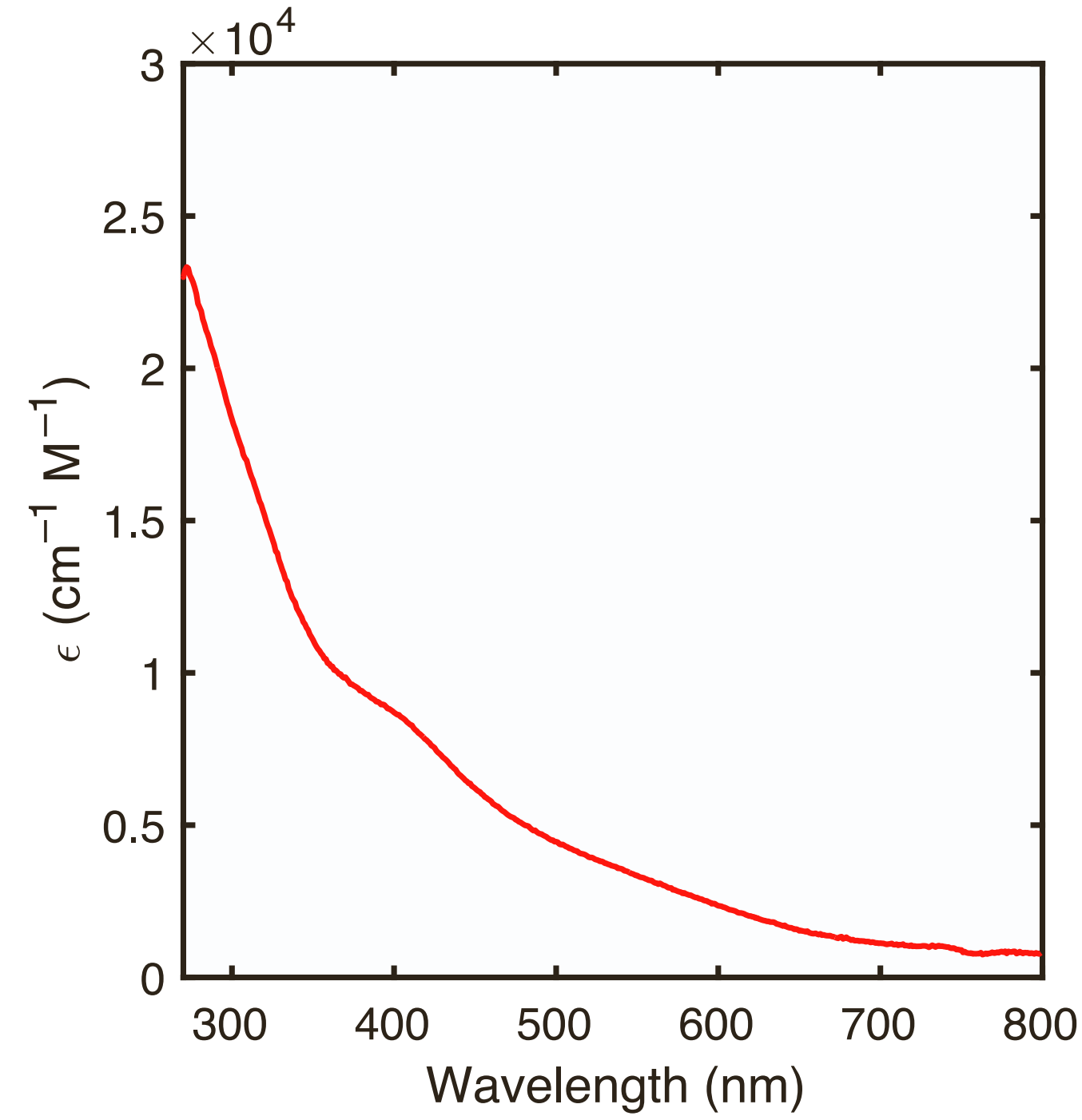

Figure S19: UV-vis spectrum of **2** in THF

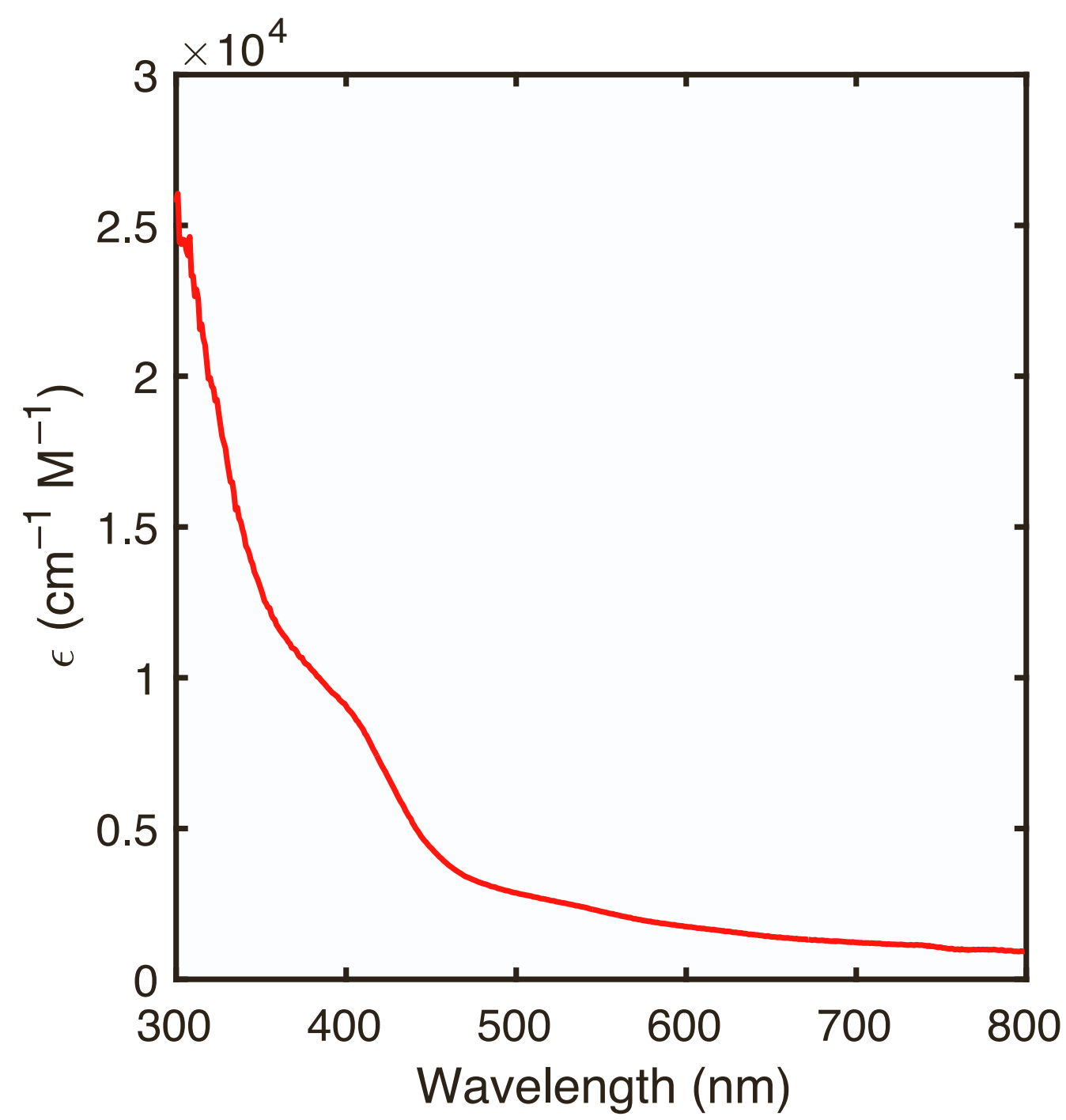

Figure S20: UV-vis spectrum of [**2**]$^{+}$ in THF

F. SQUID magnetometry

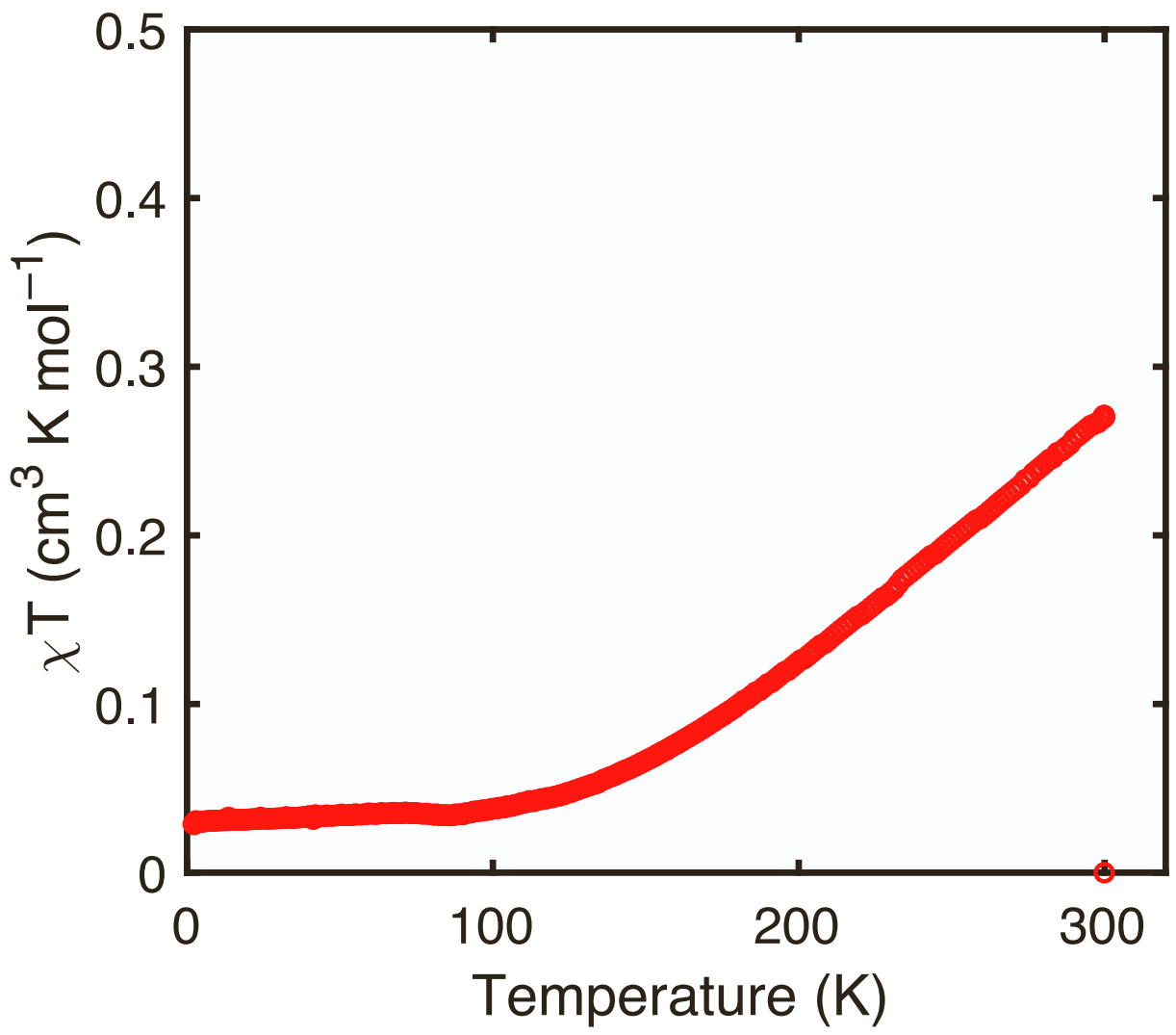

Figure S21: SQUID magnetometry data (χT vs. T) for [**1**][$BArF_4$] collected at a field of 0.5 T. Data are corrected for diamagnetic contributions using Pascal's constants. The values of χT at low temperature (below 100 K, ca. 0.02 cm^3 K mol^{-1}) are close to the expected value for an $S = 0$ system (0 cm^3 K mol^{-1}). The increase in χT with increasing temperature may be attributed to temperature independent paramagnetism (TIP) or population of higher spin excited states.

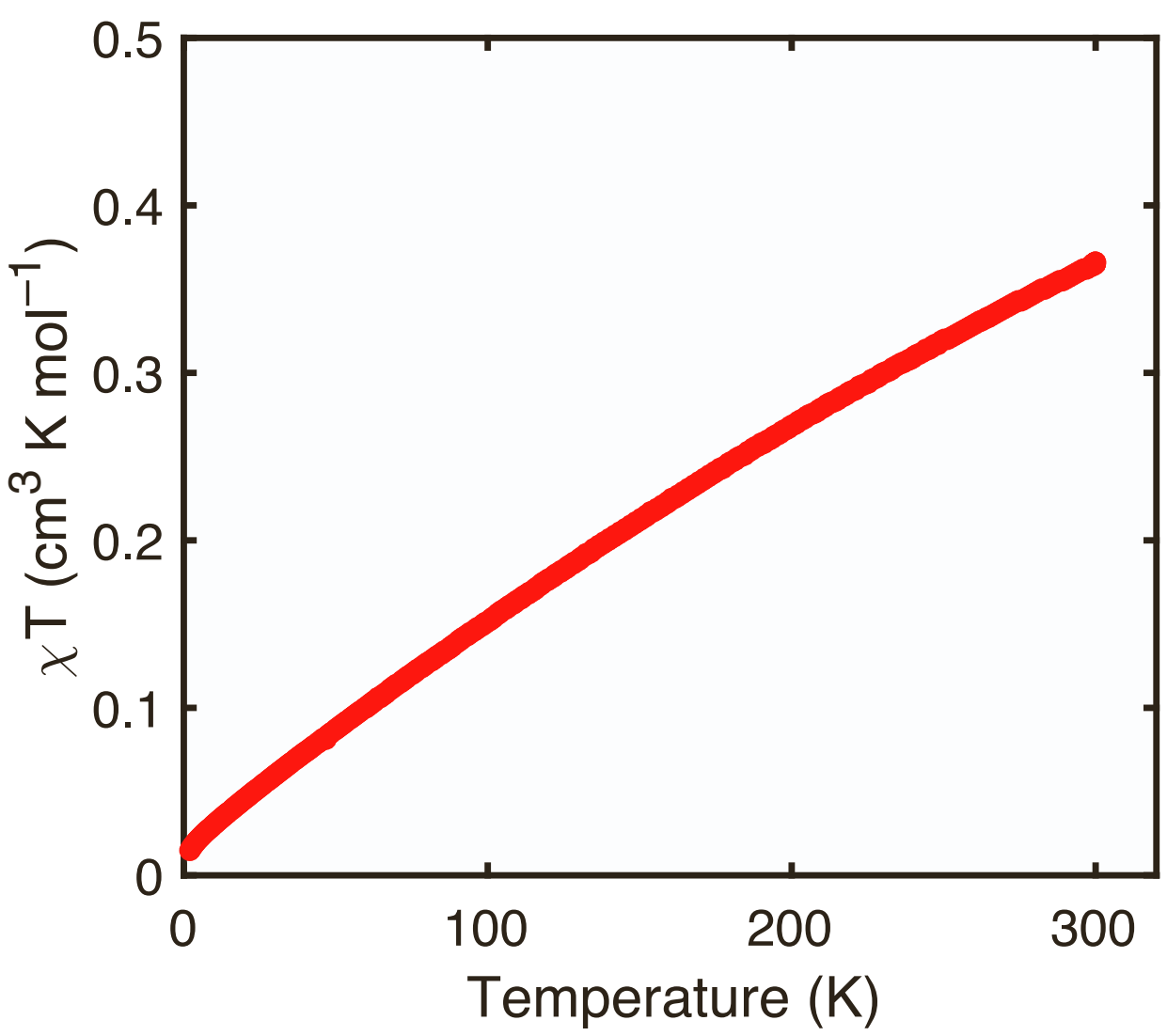

Figure S22: SQUID magnetometry data (χT vs. T) for [K(benzo-15-c-5)$_2$][**1**] collected at a field of 0.5 T. Data are corrected for diamagnetic contributions using Pascal's constants. The values of χT at low temperature (below 100 K, ca. 0.1 cm^3 K mol^{-1}) are close to the expected value for an $S = 0$ system (0 cm^3 K mol^{-1}). The increase in χT with increasing temperature may be attributed to temperature independent paramagnetism (TIP) or population of higher spin excited states.

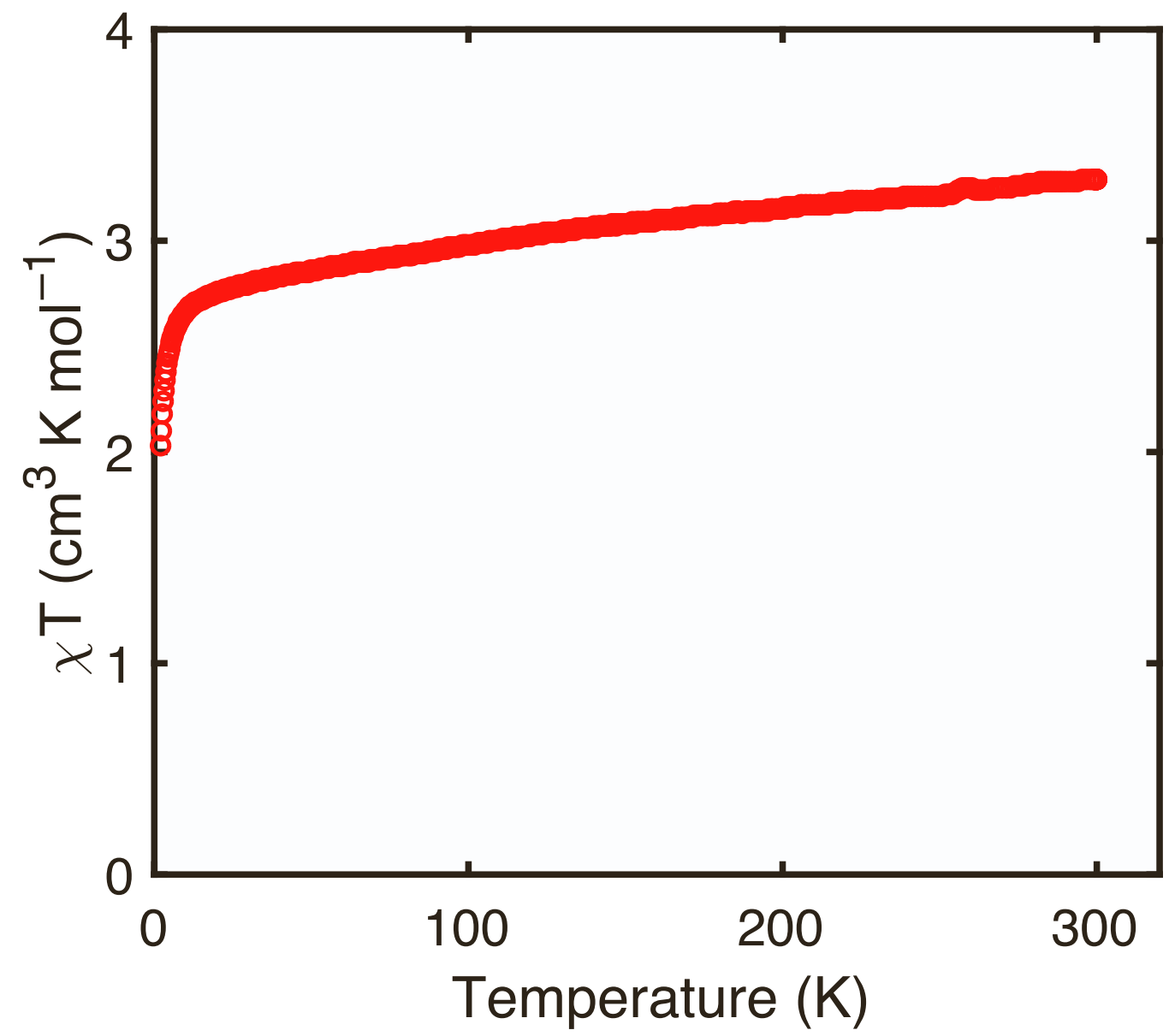

Figure S23: SQUID magnetometry data (χT vs. T) for [**2**][BAr$^F{}_4$] collected at a field of 0.5 T. Data are corrected for diamagnetic contributions using Pascal's constants. The values of χT (ca. 3 cm^3 K mol^{-1}) are close to the expected value for an $S = 2$ system (3.0 cm^3 K mol^{-1}).

G. Mössbauer spectra

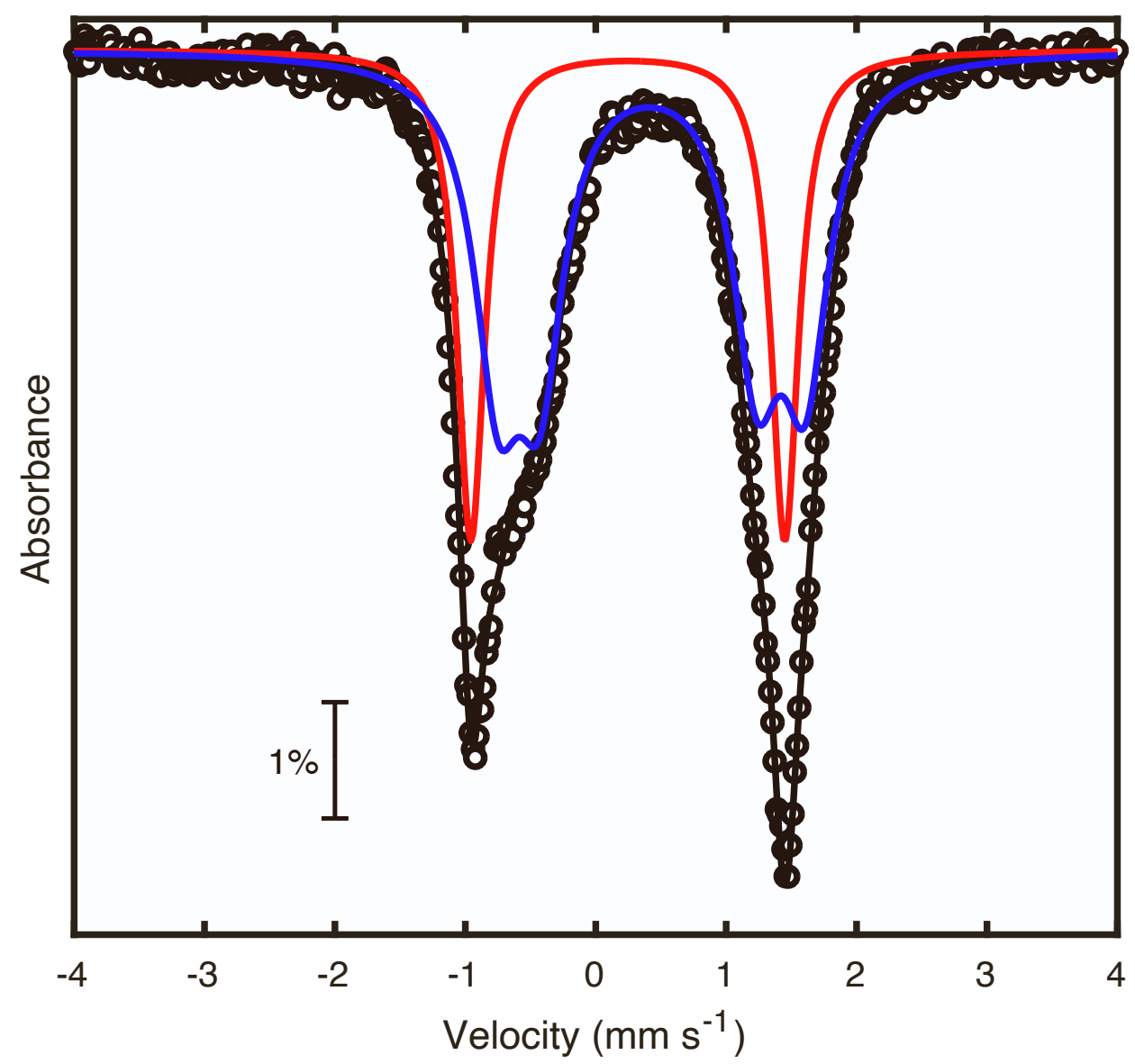

Figure S24: Mössbauer spectrum of **1** at 80 K (dots) and total simulation using the parameters in table S1 (black). The Fe–CO site is shown in red and the sum of the two Fe–NHC sites is shown in blue.

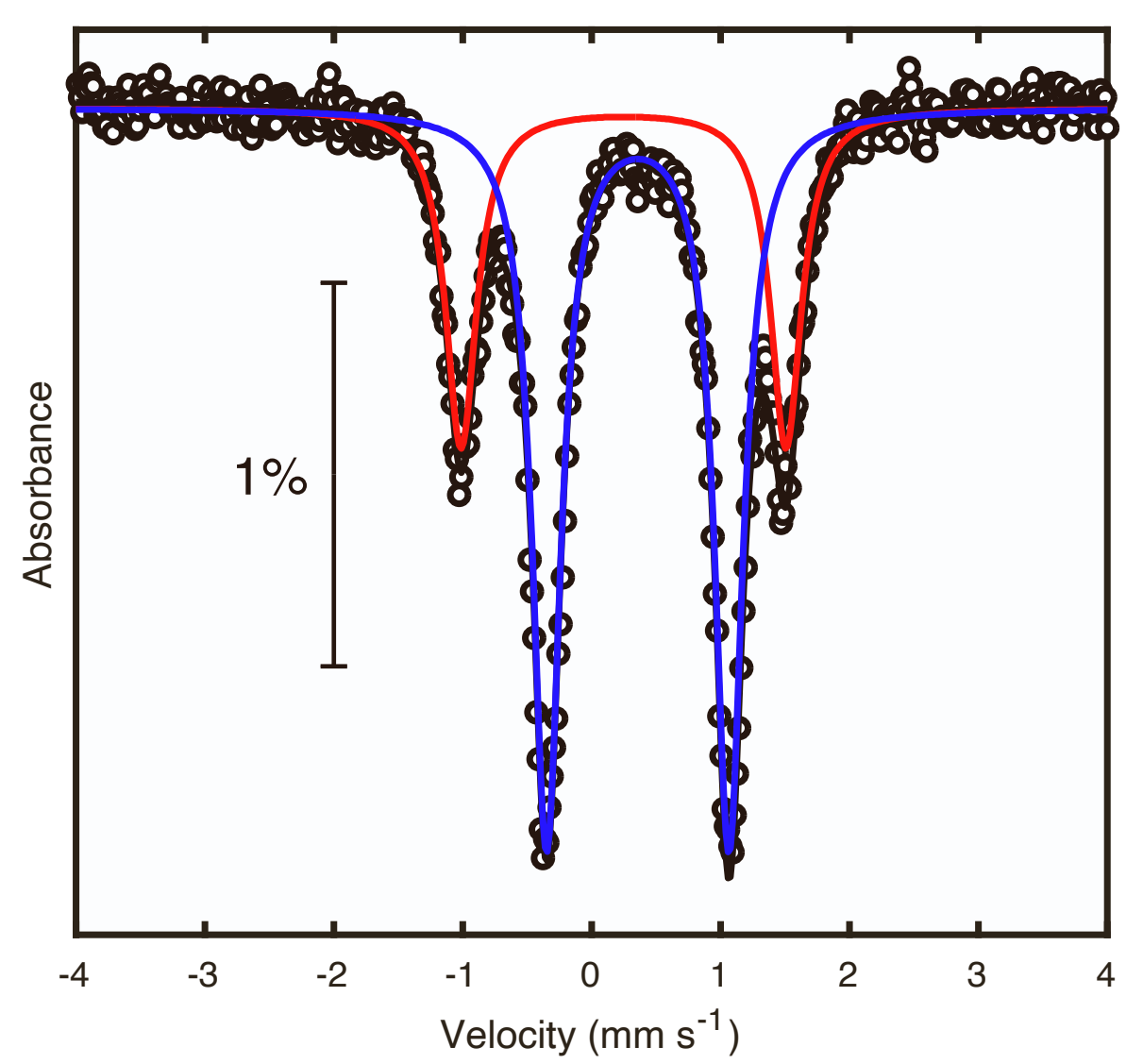

Figure S25: Mössbauer spectrum of [**1**]$^+$ at 80 K (dots) and total simulation using the parameters in table S1 (black). The Fe–CO site is shown in red and the sum of the two Fe–NHC sites is shown in blue.

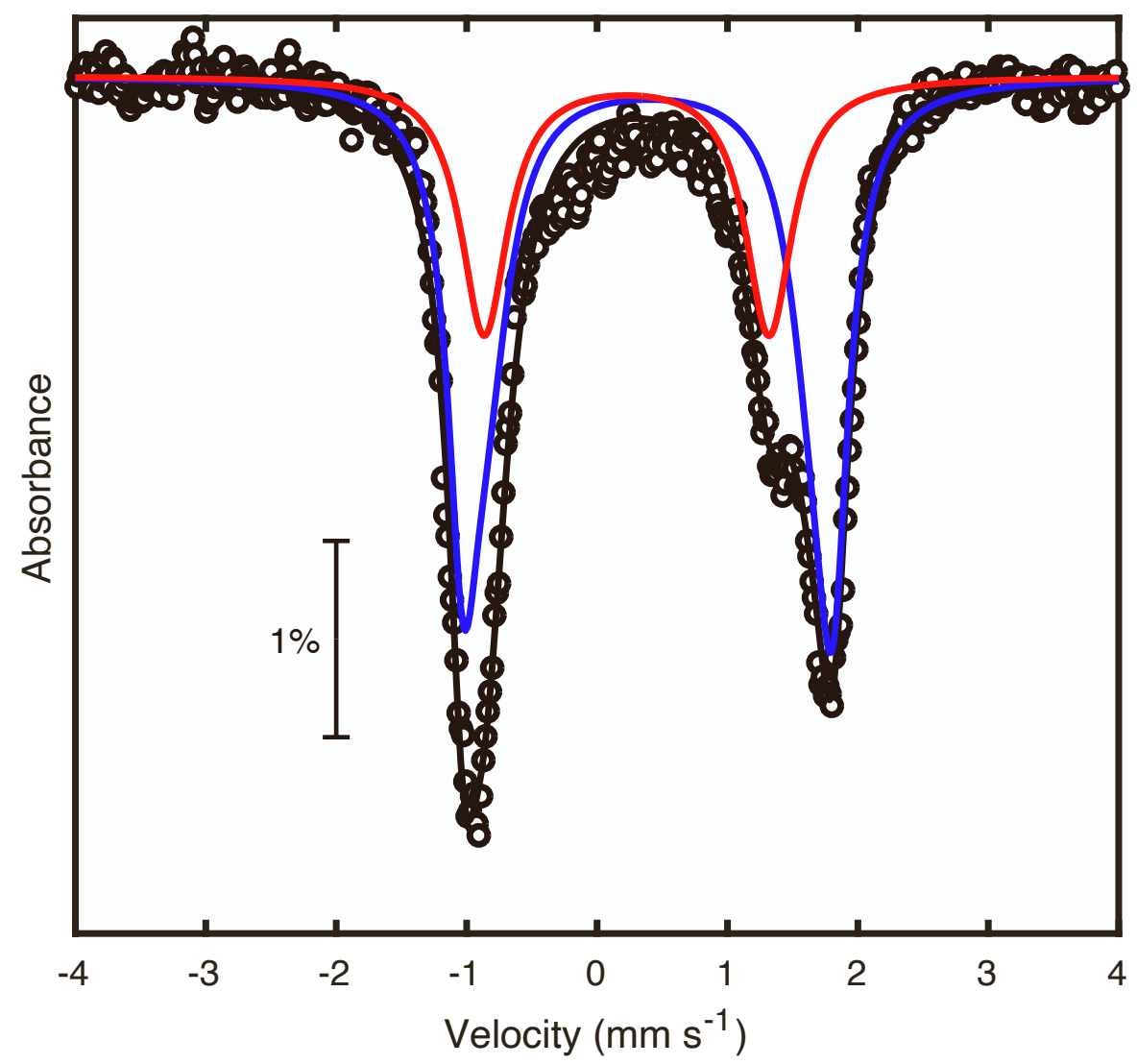

Figure S26: Mössbauer spectrum of [**1**]$^{–}$ at 80 K (dots) and total simulation using the parameters in table S1 (black). The Fe–CO site is shown in red and the sum of the two Fe–NHC sites is shown in blue.

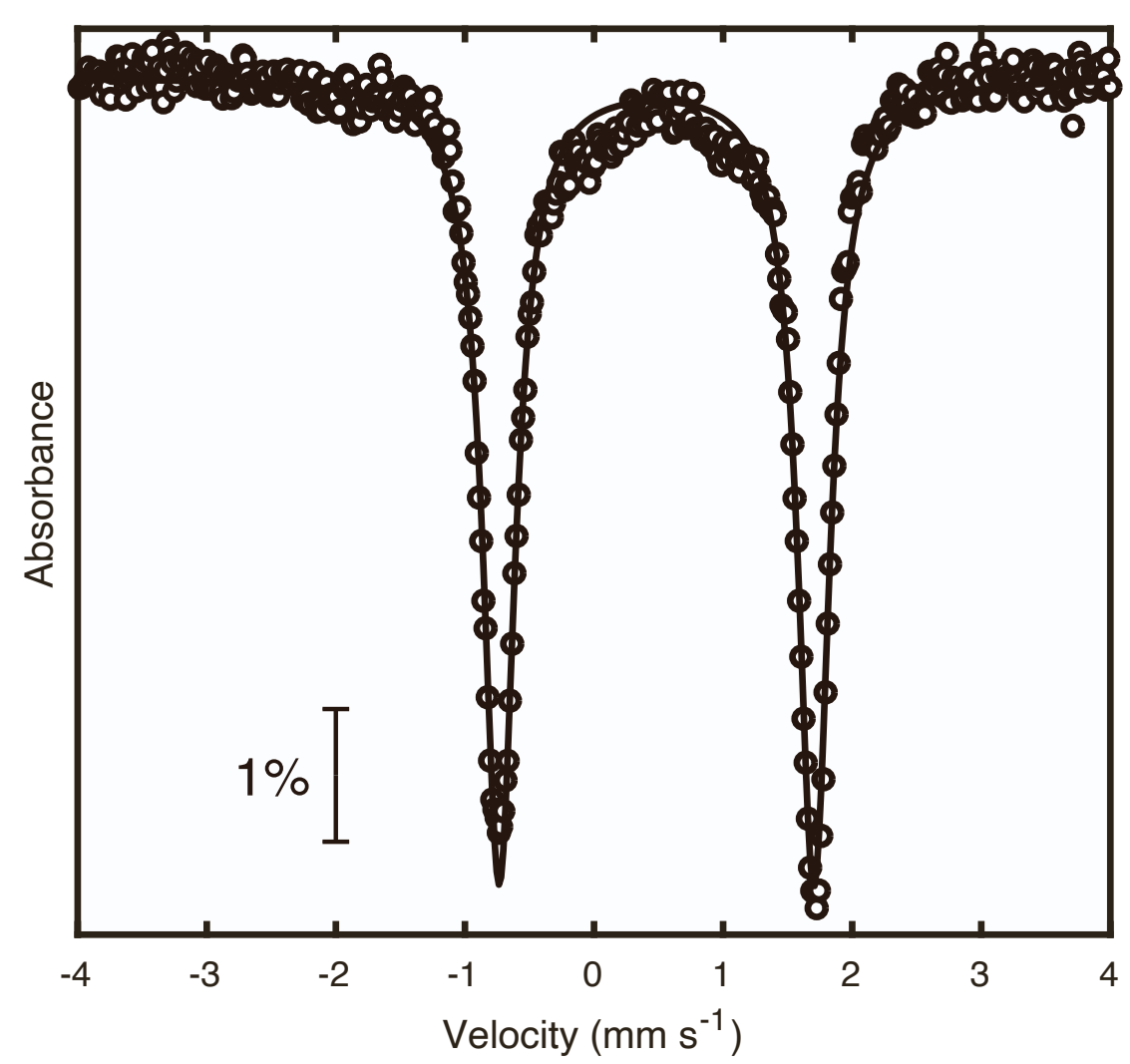

Figure S27: Mössbauer spectrum of **2** at 80 K (dots) and total simulation using the parameters in table S1 (black). All three Fe–NHC sites are simulated with one quadrupole doublet.

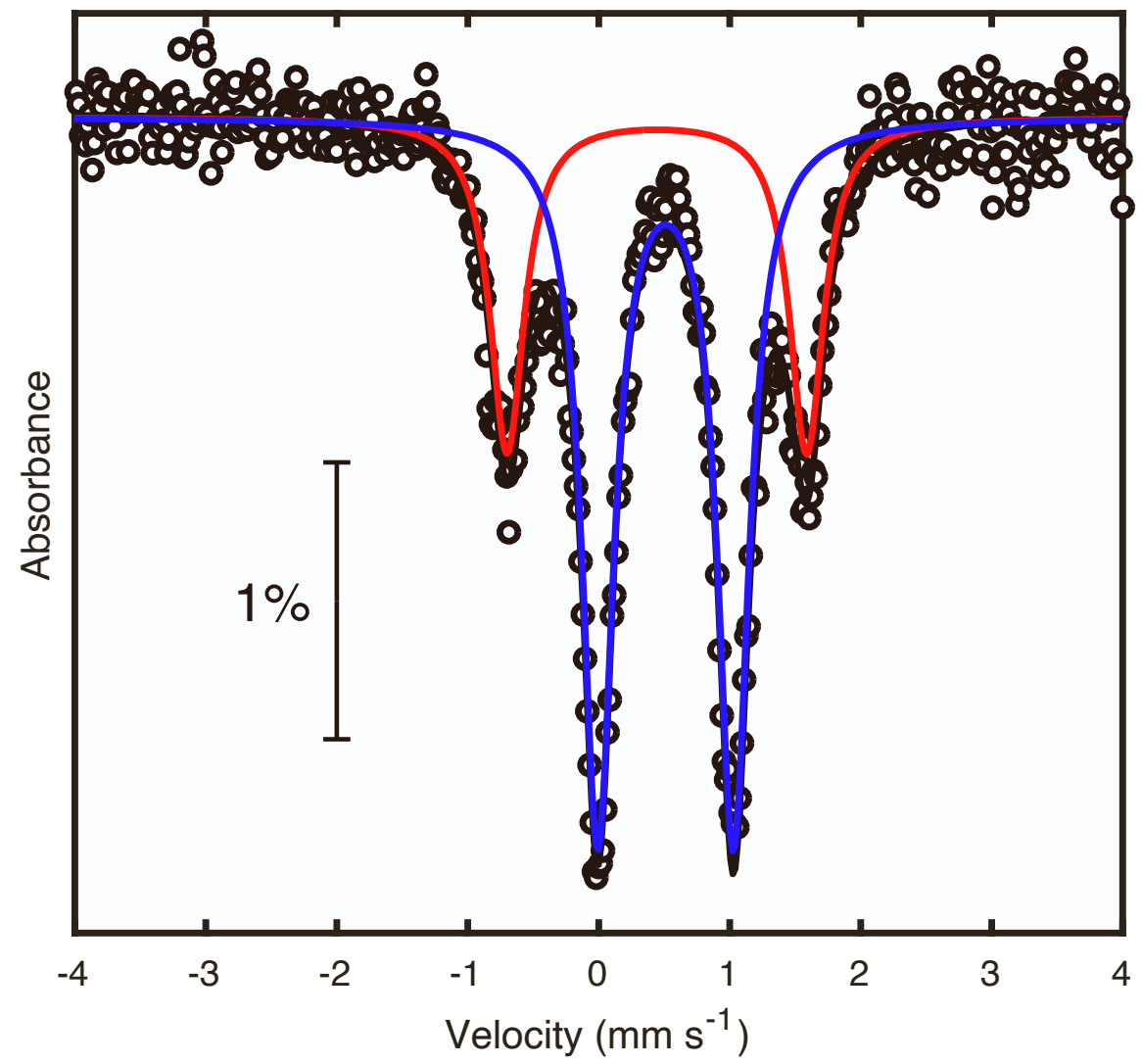

Figure S28: Mössbauer spectrum of [**2**]$^{+}$ at 80 K (dots) and total simulation using the parameters in table S1 (black). Two NHC sites are simulated with small $|\Delta E_Q|$ (blue) and one Fe–NHC site with large $|\Delta E_Q|$ (red).

Table S1: Fit parameters for Mössbauer spectrum of [**1**]$^{–/0/+}$ and [**2**]$^{0/+}$

		δ (mm·s^{-1})	$\lvert\Delta E_Q\rvert$ (mm·s^{-1})	Γ (mm·s^{-1})	Rel. Area
**1**	Fe–CO	0.246	2.406	0.257	1
	Fe–NHC (1)	0.402	1.665	0.444	1
	Fe–NHC (2)	0.429	2.357	0.435	1
[**1**]$^{+}$	Fe–CO	0.247	2.520	0.292	1
	Fe–NHC	0.358	1.414	0.268	2
[**1**]$^{–}$	Fe–CO	0.227	2.183	0.437	1
	Fe–NHC (1)	0.391	2.835	0.284	1
	Fe–NHC (2)	0.407	2.515	0.464	1
**2**	Fe–NHC	0.484	2.438	0.302	1
[**2**]$^{+}$	Fe–NHC (1)	0.444	2.287	0.317	1
	Fe–NHC (2)	0.510	1.035	0.295	2

H. Cyclic Voltammetry

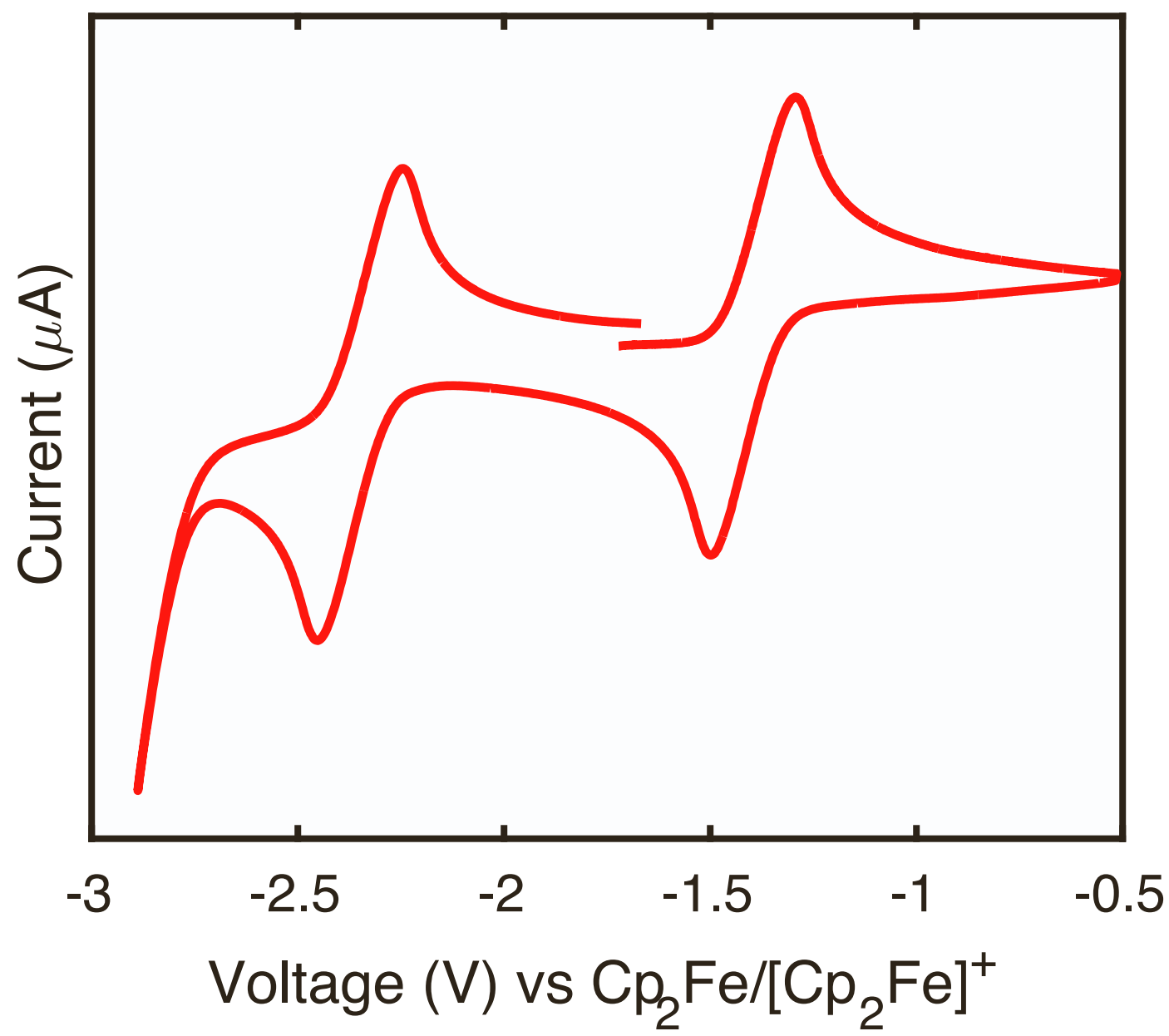

Figure S29: Cyclic voltammogram of **1** in DFB with 0.2 M TPABAr$^F{}_4$ using a glassy carbon working electrode, a silver wire counter electrode, and a silver wire reference electrode at a scan rate of 200 mv/s. Compound **1** undergoes a reduction to [**1**]$^-$ at –2.35 V and an oxidation to [**1**]$^+$ at –1.39 V vs Fc/Fc$^+$.

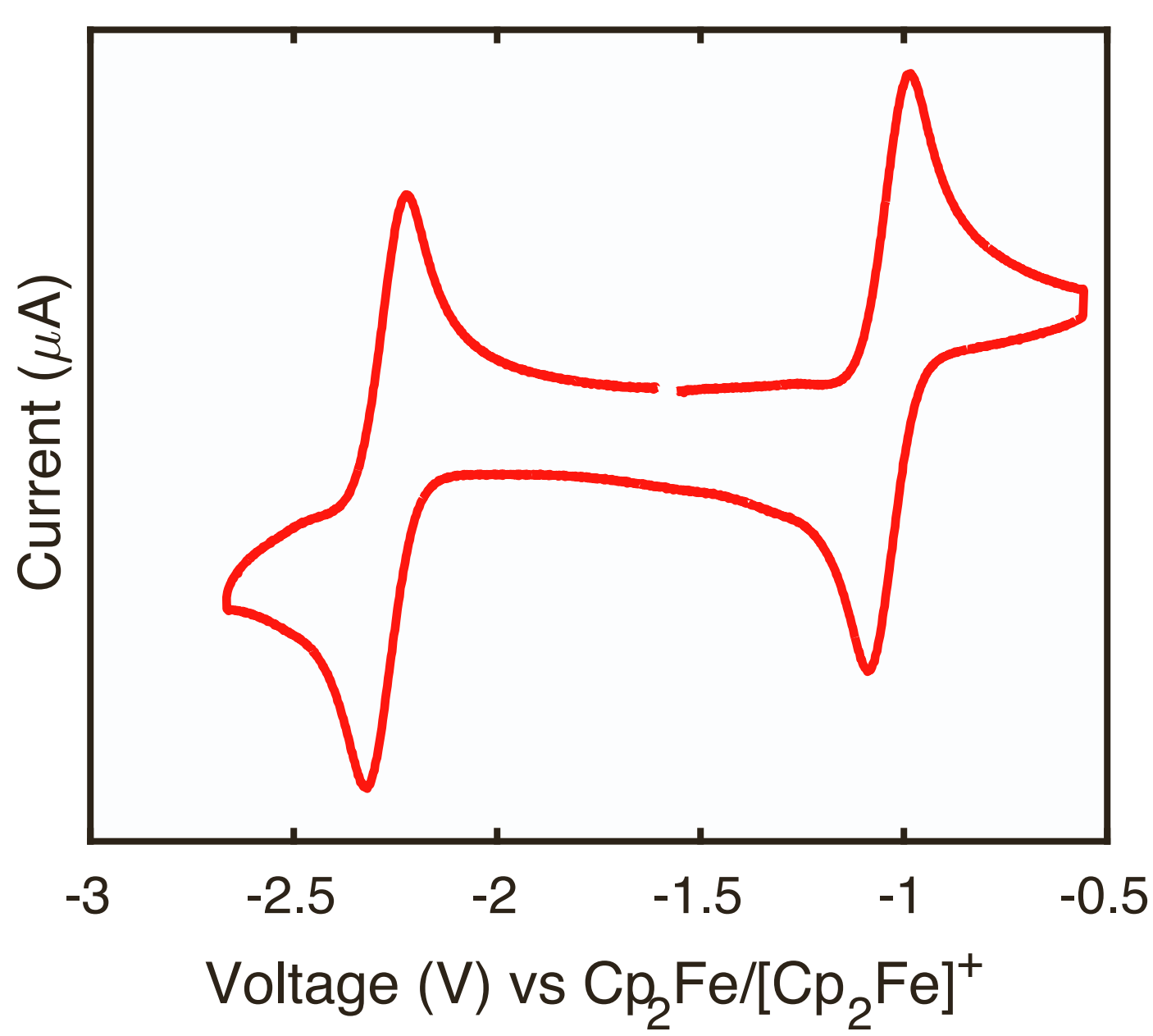

Figure S30: Cyclic voltammogram of **2** in DFB with 0.2 M TPABAr$^F{}_4$ using a glassy carbon working electrode, a silver wire counter electrode, and a silver wire reference electrode at a scan rate of 200 mv/s. Compound **2** undergoes a reduction at –2.28 V and an oxidation to [**2**]$^+$ at –1.04 V vs Fc/Fc$^+$.

Table S2: Tabulated electrochemical data for **1, 2**, **3,** and **4**. Redox couples are listed according to overall molecular charge and reported in volts vs. Fc/Fc^{+}

Compound	–1/0	0/+
**1**	–2.35	–1.34
**2**	–2.28	–1.04
**3**	–	–1.54
**4**	–	–1.91

Reduction of **1** to [**1**]$^{-}$ occurs at slightly more negative potentials than does reduction of **2**. Similarly, the oxidation of **1** to [**1**]$^{+}$ occurs at more negative potentials than does oxidation of **2** to [**2**]$^{+}$.

The opposite trend is observed for the Fe-only cluster series, **3** and **4**, where the oxidation potential is more negative for **4** than for **3**.

Drawing conclusions as to the origin of these trends is challenging because the orbitals involved in these redox events may be different.

I. Crystallographic Details

1: Crystallizes in $P2_1/m$ from THF and pentane with pentane in the lattice. The cluster resides on a mirror plane; the Cp* ring was refined as disordered 50/50 over this mirror plane. The lattice solvent was severely disordered and so solvent contributions to the diffraction pattern were removed with SQUEEZE.[9]

[**1**]$^+$: Crystallizes in P1 from DFB and pentane with four molecules in the asymmetric unit and with minor inversion twinning that was taken into account with the TWIN command and refined to a BASF value of 0.06. An additional non-merohedral twin component could be identified, but this component was minor and the structural refinement was determined to be better without inclusion of this component. Disorder in the CF_3 groups in the [BArF] anions was modeled using 1-2 and 1-3 distance restraints on the fluorine atom positions.

[**1**]$^–$: Crystallizes in P–1 from THF and pentane with pentane in the lattice. The lattice solvent was severely disordered and so solvent contributions to the diffraction pattern were removed with SQUEEZE.[9]

2: Crystallizes in P–3c1 from THF and pentane. The cluster lies on a 3-fold rotational axis and is generally well-ordered on this axis except for the Cp* ring, which was modelled in part -1. The lattice solvent was severely disordered and so solvent contributions to the diffraction pattern were removed with SQUEEZE.[9]

[**2**]$^{+}$: Crystallizes in $P2_1/n$ from DFB and HMSO with DFB in the lattice. The lattice solvent, the [BAr^F_4] anion, the Cp* ring, one IMes ligand, and one cluster Fe and 2 S atoms were disordered; The disorder in the lattice solvent, [BAr^F_4] anion, Cp* ring, and IMes ligand was modeled using similarity restraints on 1-2 and 1-3 distances and displacement parameters and rigid bond restraints. The disorder in the Fe and S positions was modeled using only similarity restraints on the displacement parameters and rigid bond restraints.

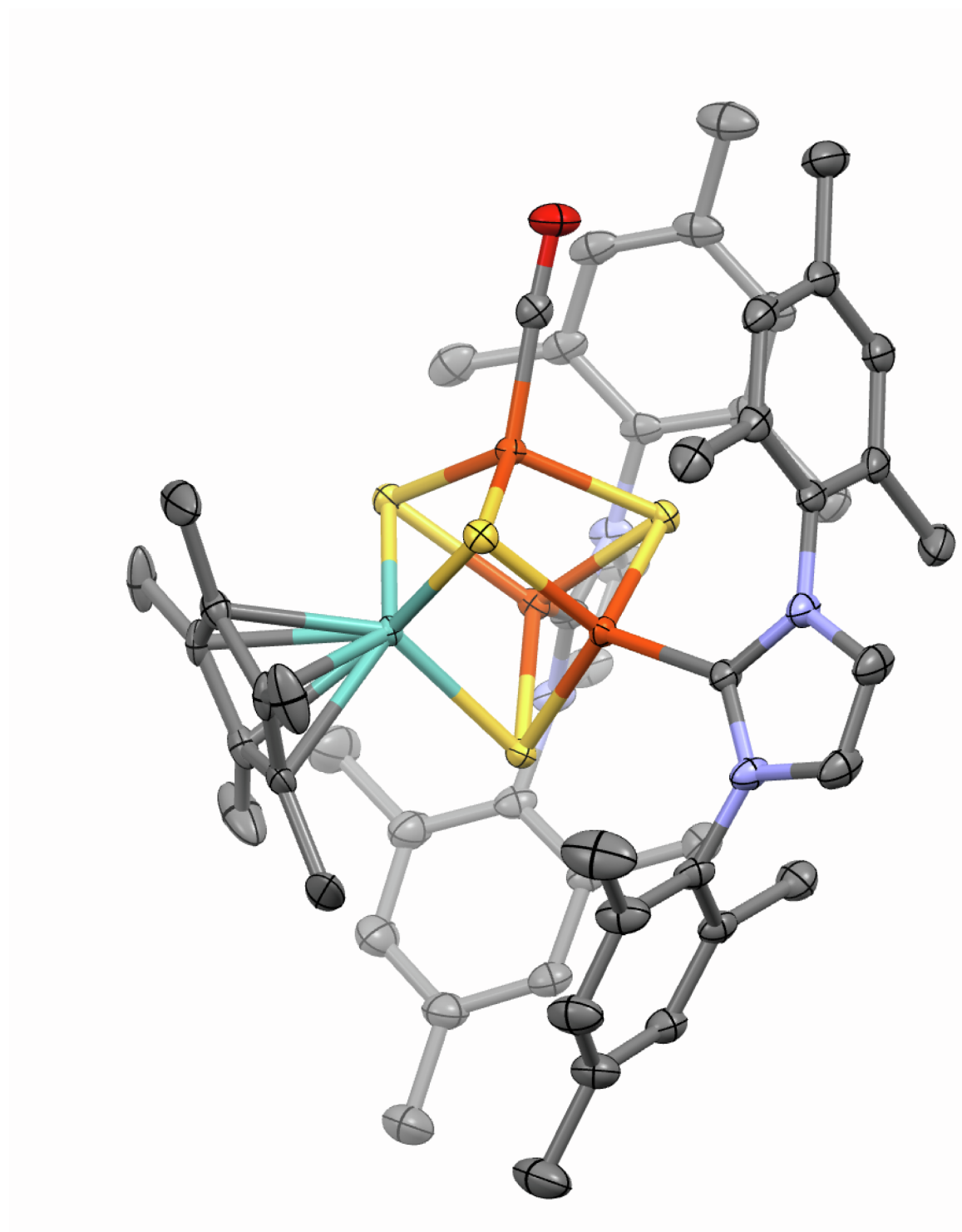

Figure S31: Crystallographic structure of **1** with ellipsoids at the 50% probability level. Carbon (gray), nitrogen (blue), iron (orange), sulfur (yellow), molybdenum (teal) and oxygen (red).

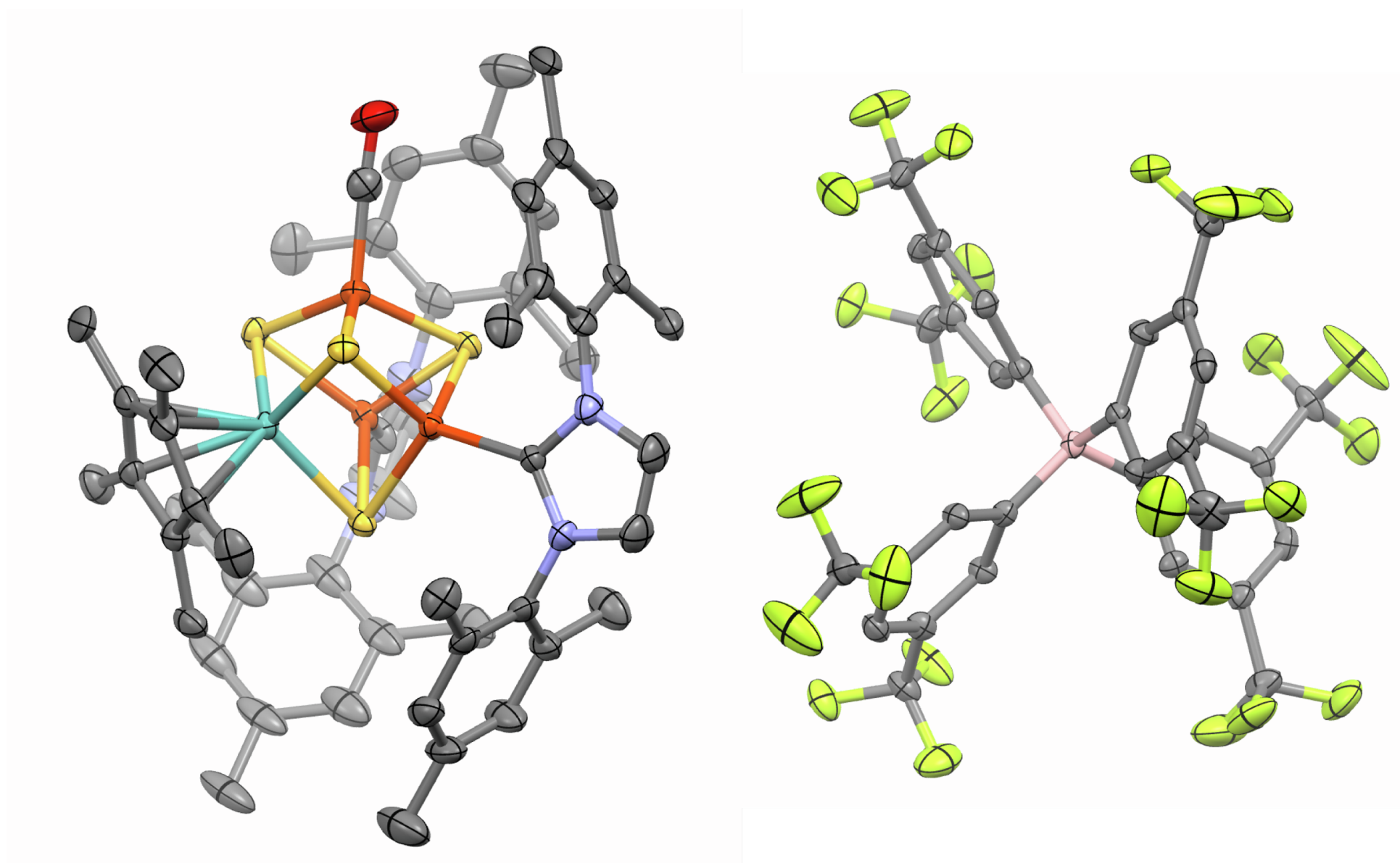

Figure S32: Crystallographic structure of $[\mathbf{1}]^{+}$ with ellipsoids at the 50% probability level. Carbon (gray), nitrogen (blue), iron (orange), sulfur (yellow), molybdenum (teal), oxygen (red), boron (pink), and fluorine (green).

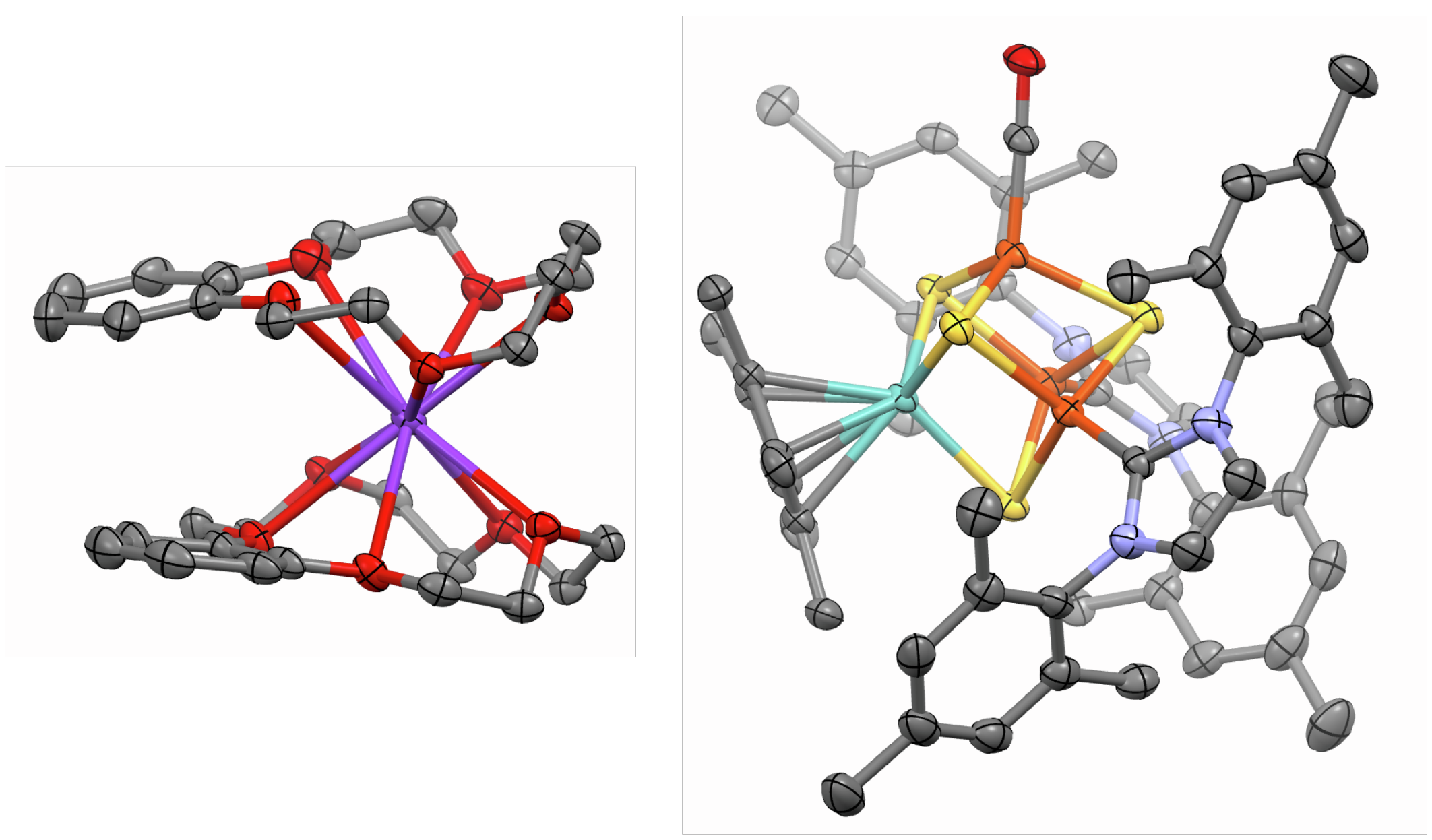

Figure S33: Crystallographic structure of $[\mathbf{1}]^{-}$ with ellipsoids at the 50% probability level. Carbon (gray), nitrogen (blue), iron (orange), sulfur (yellow), molybdenum (teal), oxygen (red), and potassium (purple).

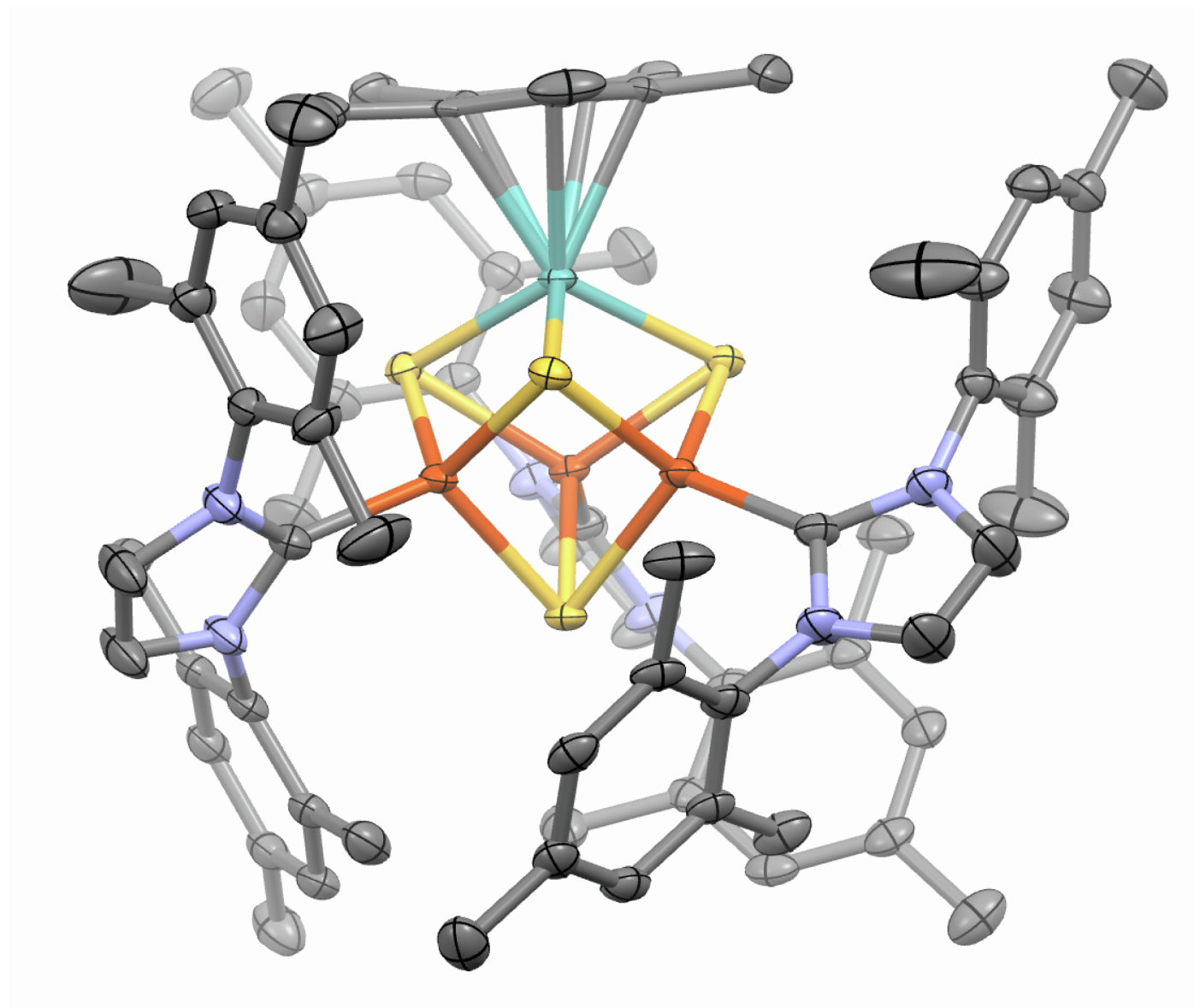

Figure S34: Crystallographic structure of **2** with ellipsoids at the 50% probability level. Carbon (gray), nitrogen (blue), iron (orange), sulfur (yellow), molybdenum (teal).

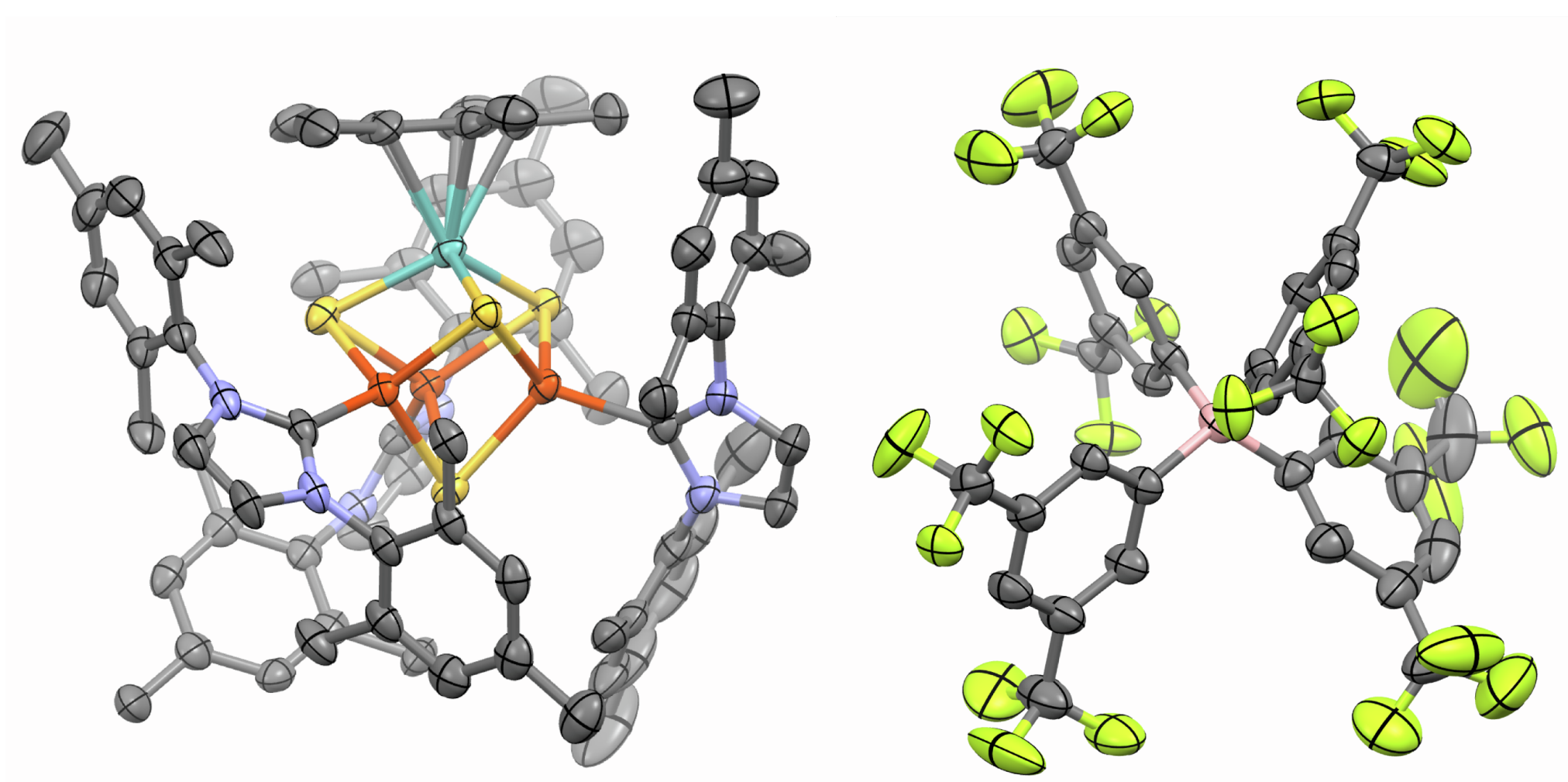

Figure S35: Crystallographic structure of [**2**][$BAr^F{}_4$] with ellipsoids at the 50% probability level. Carbon (gray), nitrogen (blue), iron (orange), sulfur (yellow), molybdenum (teal), boron (pink), and fluorine (green).

Table S3: Structural parameters for previously reported [$MoFe_3S_4$] redox series

Compound	[$MoFe_3S_4$] Charge	Fe–S (avg.)	Fe–L (avg)	Mo–L (avg.)	Mo–S (avg.)
[$TpMoFe_3S_4Cl_3$]$^{-}$	3+	2.264(2)	2.189(2)	2.209(4)	2.343(2)
[$TpMoFe_3S_4Cl_3$]$^{2-}$	2+	2.282(2)	2.254(2)	2.236(5)	2.368(1)
Δ		0.018	0.065	0.027	0.025
[$TpMoFe_3S_4CN_3$]$^{2-}$	2+	2.26(4)	2.02(1)	2.23(1)	2.36(1)
[$TpMoFe_3S_4CN_3$]$^{3-}$	1+	2.27(3)	2.00(3)	2.255(7)	2.37(1)
Δ		0.01	0.02	0.03	0.01

J. References

1. Smallcombe, S.H., Patt, S.L., and Keifer, P.A. (1995). WET Solvent Suppression and Its Applications to LC NMR and High-Resolution NMR Spectroscopy. J. Magn. Reson. Ser. A *117*, 295–303. 10.1006/jmra.1995.0759.

2. Stoll, S., and Schweiger, A. (2006). EasySpin, a comprehensive software package for spectral simulation and analysis in EPR. J. Magn. Reson. *178*, 42–55. 10.1016/j.jmr.2005.08.013.

3. Prisecaru, I. WMOSS4 Mössbauer Spectral Analysis Software, www.wmoss.org.

4. Hübschle, C.B., Sheldrick, G.M., and Dittrich, B. (2011). ShelXle: A Qt graphical user interface for SHELXL. J. Appl. Crystallogr. *44*, 1281–1284. 10.1107/S0021889811043202.

5. Sridharan, A., Brown, A.C., and Suess, D.L.M. (2021). A Terminal Imido Complex of an Iron–Sulfur Cluster. Angew. Chem. Int. Ed. *60*, 12802–12806. 10.1002/anie.202102603.

6. McSkimming, A., and Suess, D.L.M. (2021). Dinitrogen binding and activation at a molybdenum–iron–sulfur cluster. Nat. Chem. *13*, 666–670. 10.1038/s41557-021-00701-6.

7. Brown, A.C., and Suess, D.L.M. (2023). Valence Localization in Alkyne and Alkene Adducts of Synthetic $[Fe_4S_4]^+$ Clusters. Inorg. Chem. *62*, 1911–1918. 10.1021/acs.inorgchem.2c01353.

8. Kuveke, R.E.H., Barwise, L., Van Ingen, Y., Vashisth, K., Roberts, N., Chitnis, S.S., Dutton, J.L., Martin, C.D., and Melen, R.L. (2022). An International Study Evaluating Elemental Analysis. ACS Cent. Sci. *8*, 855–863. 10.1021/acscentsci.2c00325.

9. Spek, A.L. (2015). PLATON SQUEEZE: A tool for the calculation of the disordered solvent contribution to the calculated structure factors. Acta Crystallogr. Sect. C Struct. Chem. *71*, 9–18. 10.1107/S2053229614024929.
